# Synthesis of Chiral TFA-Protected α-Amino Aryl-Ketone Derivatives with Friedel–Crafts Acylation of α-Amino Acid *N*-Hydroxysuccinimide Ester

**DOI:** 10.3390/molecules22101748

**Published:** 2017-10-17

**Authors:** Zetryana Puteri Tachrim, Kazuhiro Oida, Haruka Ikemoto, Fumina Ohashi, Natsumi Kurokawa, Kento Hayashi, Mami Shikanai, Yasuko Sakihama, Yasuyuki Hashidoko, Makoto Hashimoto

**Affiliations:** Division of Applied Bioscience, Graduate School of Agriculture, Hokkaido University, Kita 9, Nishi 9, Kita-ku, Sapporo 060-8589, Japan; z317_style@live.com (Z.P.T.); 643.get.two.oidia6@gmail.com (K.O.); i.h.cooking4989@gmail.com (H.I.); fumina28ohsei@gmail.com (F.O.); natsumi.k0420@gmail.com (N.K.); auland0225@gmail.com (K.H.); mami-s@ec.hokudai.ac.jp (M.S.); sakihama@abs.agr.hokudai.ac.jp (Y.S.); yasu-h@abs.agr.hokudai.ac.jp (Y.H.)

**Keywords:** *N*-hydroxysuccinimide ester, Friedel–Crafts acylation, α-amino acid, α-amino aryl-ketone, racemization

## Abstract

Chiral *N*-protected α-amino aryl-ketones are one of the useful precursors used in the synthesis of various biologically active compounds and can be constructed via Friedel–Crafts acylation of *N*-protected α-amino acids. One of the drawbacks of this reaction is the utilization of toxic, corrosive and moisture-sensitive acylating reagents. In peptide construction via amide bond formation, *N*-hydroxysuccinimide ester (OSu), which has high storage stability, can react rapidly with amino components and produces fewer side reactions, including racemization. This study reports the first synthesis and utilization of *N*-trifluoroacetyl (TFA)-protected α-amino acid-OSu as a potential acyl donor for Friedel–Crafts acylation into various arenes. The TFA-protected isoleucine derivative and its diastereomer TFA-protected *allo*-isoleucine derivative were investigated to check the retention of α-proton chirality in the Friedel–Crafts reaction. Further utilization of OSu in other branched-chain and unbranched-chain amino acids results in an adequate yield of TFA-protected α-amino aryl-ketone without loss of optical purity.

## 1. Introduction

Chiral *N*-protected α-amino aryl-ketone are usually used as a precursor in the synthesis of various biologically active compounds [1,2]. The most favorable strategy to synthesize α-amino aryl-ketone is by Friedel–Crafts acylation [3,4] of arenes, which is known as a reliable method that results in satisfactory product yields [5] and can utilize convenient optically pure α-amino acid derivatives as skeletons [6]. The α-amino acid chloride [1,2,7,8] is widely used as an acyl donor to undergo Friedel–Crafts acylation due to its reactivity. However, this acyl donor is unstable, sensitive to moisture and difficult to handle [9]. Therefore, it should be used instantly and cannot be stored. In some case, α-amino acid chloride utilization also cannot prevent the chirality [1,9]. Moreover, α-amino acid anhydride can be employed as an acylating agent and can be used to obtain the α-amino aryl-ketone without the *N*-protecting group. Unfortunately, α-amino acid anhydride only shows reactivity for electron-rich arene acyl acceptor [10] and needed to be prepared by using a toxic gas, such as phosgene or triphosgene [11]. Since α-amino acid anhydride consists of two possible α-amino acids, one of the unacylated molecules will be wasted during the reaction. Recently, *N*-protected *N*-(α-aminoacyl)benzotriazole has been reported to acylate benzene by using Lewis acid [12]. Despite this *N*-acylbenzotriazole being more convenient to handle compared to α-amino acid chloride, only a moderate yield of α-aminoacyl phenyl-ketone can be synthesized. However, the preparation of *N*-protected *N*-(α-aminoacyl)benzotriazole [12,13,14] was conducted under in situ conditions, which indicated that its isolation takes more effort.

*N*-hydroxysuccinimide ester (OSu) derivatives of α-amino acids have high storage stability [15] and have enough reactivity with amino components under moderate conditions. OSu modified in α-amino acid derivatives are adequately commercially available, which indicate that it would be a convenient reagent in peptide synthesis. The OSu modified in α-amino acid is also known to facilitate peptide synthesis as a good alkoxy-leaving group in the early 1950s [16]. This utility indicated that OSu derivatives may act as an acyl donor for the Friedel–Crafts reaction. In peptide construction, it is known to produce fewer side reactions, including racemization. The Friedel–Crafts acylation of electron-rich arene (ferrocene and pyrene) with *N*-hydroxysuccinimidyl of benzoic or *p*-methoxybenzoic acid is previously reported, which is activated by superacidic trifluoromethanesulfonic acid [17]. Until now, no study has reported the use of α-amino acid-OSu as a representative skeleton for direct Friedel–Crafts acylation. In this work, the synthesis and properties of a potential acyl donor for Friedel–Crafts acylation, namely *N*-trifluoroacetyl (TFA)-protected α-amino acid-OSu, are described. We demonstrated the utility of optically active isoleucine, which has two chiral centers in the molecules, and its diastereomer *allo*-isoleucine to identify chilarity of the Friedel–Crafts acylation product’s α-proton by nuclear magnetic resonance (NMR). The application of α-amino acid-OSu derivatives for synthesis of TFA-protected α-amino aryl-ketone is assumed to retard its chirality which might act as a potential acyl donor for the Friedel–Crafts acylation.

## 2. Results and Discussion

**Figure 1:**
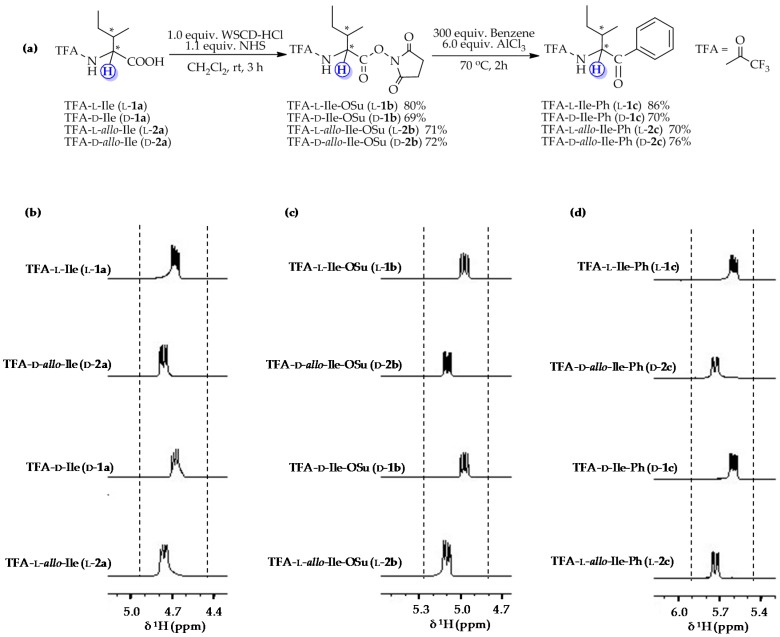
Synthesis and application of TFA-l-/d-Ile-OSu (l-/d-**1b**) and TFA-l-/d-*allo*-Ile-OSu (l-/d-**2b**). (**a**) Synthesis of l-/d-**1b** and l-/d-**2b** and its application for acylation of benzene by conventional Friedel–Crafts conditions; (**b**) Selected ^1^H-NMR of TFA-l-/d-Ile (l-/d-**1a**) and TFA-l-/d-*allo*-Ile (l-/d-**2a**); (**c**) Selected ^1^H-NMR of l-/d-**1b** and l-/d-**2b**; (**d**) Selected ^1^H-NMR of TFA-l-/d-Ile-Ph (l-/d-**1c**) and TFA-l-/d-*allo*-Ile-Ph (l-/d-**2c**). (Full NMR data was available in Supplementary Materials Scheme SM-2).

l-Isoleucine is known to have the ability to undergo epimerization at the α-position to produce d-*allo*-isoleucine. As for chiral *N*-protected α-amino aryl-ketone synthesis, especially the reaction involving direct acylation of α-amino acids, the product’s chirality is commonly determined by complexation with a chiral shift reagent [7]. This racemic α-amino aryl-ketone can be detected by the appearance of two separated proton signals by NMR. Up to now, there is no reported study that has checked the chirality of the TFA-protected α-amino aryl-ketone based on isoleucine and its diastereomer *allo*-isoleucine. Since the asymmetric carbon at α-position within these stereoisomers can be detected by ^1^H-NMR [18], utilization within isoleucine and its diastereomer *allo*-isoleucine (four types of stereoisomers; Figure 1) might be useful in describing a change in configuration of their chiral center during the chemical modification.

Initially in our study, the corresponding amino group of optical active l-/d-isoleucine and l-/d-*allo*-isoleucine underwent TFA protection by using ethyl trifluoroacetate in the presence of triethylamine in methanol [19,20] to generate TFA-l-/d-Ile (l-/d-**1a**) and TFA-l-/d-*allo*-Ile (l-/d-**2a**; Figure 1a; see Supplementary Materials Scheme SM-1 and Table SM-1). The optically active l-/d-**1a** and l-/d-**2a** show nonequivalence signals arising from epidemically related α-proton (Figure 1b). The comparison between l-/d-**1a** (δ = 4.68 ppm) and its diastereomer l-/d-**2a** (δ = 4.76 ppm) show the chemical shift differences of α-proton appearance at ^1^H-NMR, which is suggested as a sufficient method for checking the epimerization during the reaction. In this study, the protection by TFA is preferred due to its stability in the presence of Lewis acid, which is an essential reagent for conventional Friedel–Crafts acylation, compared with most carbamate-based protecting groups, such as *t*-butyl (BOC), benzyl (Cbz) or fluorenylmethyloxycarbonyl (Fmoc). The optically active l-/d-**1a** and l-/d-**2a** was transformed into TFA-l-/d-Ile-OSu (l-/d-**1b**) and TFA-l-/d-*allo*-Ile-OSu (l-/d-**2b**) within 3 h at room temperature by utilization of 1.1 equiv. NHS (*N*-hydroxysuccinimide) and 1.0 equiv. of WSCD-HCl (water soluble carbodiimide hydrochloride) in CH_2_Cl_2_ (Figure 1a). The characterization of l-/d-**1b** and l-/d-**2b** by ^1^H-NMR can serve the purpose for checking the chirality preservation [18] during the modification or reaction process, of which only the α-proton signal of l-/d-**1b** in ^1^H-NMR (δ = 4.97 ppm, Figure 1c) can be observed. No trace of l-/d-**2b** (δ = 5.10 ppm) was detected (Figure 1c).

The l-/d-**1b** and l-/d-**2b** are stable for storage at −20 °C for more than six months and can be readily used as an acyl donor to undergo Friedel–Crafts acylation into benzene. The preliminary study for the variance of the proportion of AlCl_3_ utilized for the acylation of l-**1b** into benzene is shown at 70 °C (Table 1, Entries 1–3). Less than 3 equiv. of AlCl_3_ did not result in any Friedel–Crafts product (Table 1, Entries 1–2). Therefore, utilization of 6 equiv. AlCl_3_ is suitable for acylation in which only Friedel–Crafts product of TFA-l-Ile-Ph (l-**1c**) was obtained with a good yield within 2 h (Table 1, Entry 3). Based on ^1^H-NMR (Figure 1d), the chiral center at α-position of l-/d-**1c** at δ = 5.60 ppm clearly shows no appearance of any nonequivalence signals, which contribute to d-**2c** (δ = 5.74 ppm). This indicates that either l-/d-**1c** or its diastereomer is different in chiral center only at the α-position of l-/d-**2c** and it shows no epimerization after the acylation. Compared with the acylation of aryl *N*-hydroxysuccinimide ester derivatives and electron-rich arenes, such as ferrocene and pyrene [17], l-/d-**1b** and l-/d-**2b** are reactive in relatively electron-poor acyl acceptors of benzene when conventional AlCl_3_ is used. Thus, the activation is not necessary under harsh conditions, such as utilization of super acidic trifluoromethanesulfonic acid [5,6]. Moreover, l-/d-**1b** and l-/d-**2b** can dissolve well in benzene and thus, no solvent is needed for the reaction system. In comparison, the reported *N*-protected *N*-(α-aminoacyl)benzotriazole, which was previously used as an acyl donor for the Friedel–Crafts reaction, showed low solubility for the selected acyl acceptor and utilized CH_2_Cl_2_ as the solvent [12]. However, when isoleucine derivative and its diastereomer *allo*-isoleucine derivative are utilized for checking the chiral center by ^1^H-NMR, it can offer a simple analysis that is able to comprehensively examine all the processes of TFA-protected α-amino acid-OSu synthesis as the acyl donor of Friedel–Crafts acylation. To support our understanding, the optical rotation of all corresponding l-/d-α-amino acid derivatives was also conducted for checking the retention of α-proton chirality during the process. Moreover, based on our finding of α-protons in ^1^H-NMR (Figure 1d), it appears that the acylium ion formation of the common Friedel–Crafts intermediate successfully retarded the α-proton chirality.

The screening of Lewis acid utilization for acylation of l-**1b** into benzene has been examined. When the conventional catalyst AlCl_3_ (Table 1, Entry 3) was replaced with other common Lewis acids (Table 1, Entries 4–7) under the same reaction conditions (6 equiv. Lewis acid was used at 70 °C), instead of desired product formation, we found that hydrolysis occurred and l-**1a** was detected after quenching the reaction mixture. The other metal halide of GaCl_3_ contributed to the acylation of the result in l-**1c** (Table 1, Entry 8). Compared with GaCl_3_, InCl_3_ showed the lowest reactivity due to the starting material of l-**1b** being recovered after the reaction mixture was quenched (Table 1, Entry 9). However, although the l-**1c** was produced from acylation utilized by GaCl_3_ and showed retention of α-proton chirality, the chemical yield of the desired α-amino phenyl-ketone is far less than that produced with the reaction utilizing AlCl_3_. If excess AlCl_3_ were used, the Lewis acid apparently will coordinate to the most basic site [21] (the carbonyl oxygen atoms of OSu and *N*-acyl groups) and Friedel–Crafts acylation can take place to result in a high yield of the desired product. Therefore, AlCl_3_ is preferred to undergo the reaction due to its high reactivity and also the fact that it has a considerably lower cost that brings economic benefits.

**Scheme 1:**
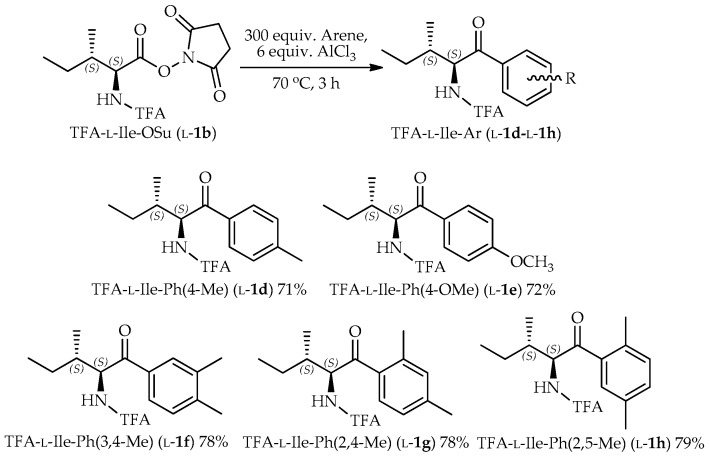
Friedel–Crafts reaction of TFA-l-Ile-OSu (l-**1b**) into various arenes that were catalyzed by AlCl_3_ to result in TFA-l-Ile-Ar (l-**1d**–l-**1h**).

For further applications of α-amino acid-OSu as a potential acyl donor that can be easily activated by the conventional Friedel–Craft catalyst of AlCl_3_, we tried to use l-**1b** in acylation reactions to convert it into various arenes (Scheme 1). l-**1b** reacted with arenes when 6 equiv. AlCl_3_ was added for 3 h. TFA-l-Ile-Ar (l-**1d**–l-**1h**) was produced with a high yield. The acylation into electron-donating arenes, such as toluene, anisole, and *m*-xylene mainly occurs at a less hindered position (ratio of *p*- and *o*-position for compound l-**1d**, l-**1e**, l-**1g** is 5:1, 18:1 and 11:1, respectively). From ^1^H-NMR (see Supplementary Materials Scheme SM-2), only the α-proton signal of desired l-**1d**–l-**1h** can be observed which implies that there is no other diastereomer showing the α-proton chirality retention of TFA-protected α-amino aryl-ketone. Unlike TFA-protected α-amino acid-OSu that can utilize various commercially available acyl acceptors, Weinreb amides (*N*-methoxy-*N*-methylamides) [22] (popular methods for the synthesis of α-amino aryl-ketones [23]) are considerably less efficient for direct acylation due to limitation of Grignard or organolithium reagents, which sometimes need to be synthesized before use. Moreover, the limited study of TFA-protected α-amino aryl-ketone synthesis during the last decade is due to the extremely basic condition of the Weinreb amides system, in which carbamate-based protected α-amino acid is preferred to undergo the reaction.

**Scheme 2:**
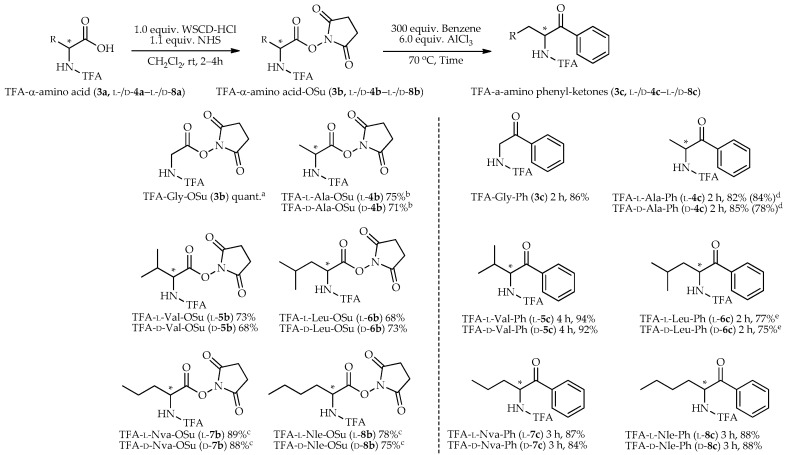
Synthesis and application of TFA-α-Amino Acid-OSu (**3b**, l-/d-**4b**–l-/d-**8b**). ^a^ Utilization of dimethylformamide as solvent and 1.0 equiv. *N*-hydroxysuccinimide (NHS). The reaction was conducted at room temperature for 1 h. ^b^ WSCD-HCl was directly added into the reaction and the reaction mixture was directly washed by sat. NaCl to purify the product. ^c^ Utilization of 1.1 equiv. WSCD-HCl at 0 °C. ^d^ Reaction at room temperature for 36 h. ^e^ Calculated from ^1^H-NMR.

Other natural and unnatural α-amino acids are subjected to Friedel–Crafts reactions (Scheme 2). glycine and optically pure alanine, valine, and leucine, norvaline, and norleucine were protected by TFA to result in TFA-protected α-amino acid (**3a**, l-/d-**4a**–l-/d-**8a**) and directly converted into TFA-protected α-amino acid-OSu (**3b**, l-/d-**4b**–l-/d-**8b**; see Supplementary Materials Scheme SM-1). Next, conventional Friedel–Craft conditions are applied for the acylation of TFA-protected α-amino acid-OSu (**3b**, l-/d-**4b**–l-/d-**8b**) with benzene to produce TFA-protected α-amino acid-Ph (**3c**, l-/d-**4c**–l-/d-**8c**, Scheme 2). In the previous study, TFA *N*-(α-aminoacetyl)benzotriazole was reported to be converted to acylate benzene when using AlCl_3_ with CH_2_Cl_2_ as a solvent at 20 °C for 3 h, and produced a moderate yield of α-aminoacetyl phenyl-ketone (63% yield) [12]. In comparison, TFA-l-/d-Ala-OSu (l-/d-**4b**) utilization can result in a high yield of TFA-l-/d-Ala-Ph (l-/d-**4c**, 82–85%, Scheme 2) at 70 °C for 2 h. Unlike reaction acylation with TFA *N*-(α-aminoacyl)benzotriazole that needs CH_2_Cl_2_ as a solvent, l-/d-**4b** can be easily dissolved in excess benzene. Thus, it implies that the reaction is more efficient and produces considerably less organic solvent waste. Moreover, l-/d-**4b** can also cause the acylation of benzene at room temperature for 36 h to result in a high yield (l-/d-**4c** 78–84%, Scheme 2), which ensured its reactivity as a potential acyl donor for this reaction.

In this study, the introduction of two pure enantiomers of l- and d-α-amino acids showed identical optical rotation with the opposite sign after being modified into TFA-protected α-amino acid-OSu and utilized as potential acyl donors to synthesize TFA-protected α-amino aryl-ketone. Hence, the construction of either TFA-protected α-amino acid-OSu or its application for acylation under Friedel–Craft conditions shows the retention of α-proton chirality. In line with the utilization of l-/d-isoleucine and its diastereomer l-/d-*allo*-isoleucine that showed no epimerization during the modification, TFA-protected α-amino acid-OSu is applicable for various α-amino acids and can broadly be used for the various syntheses of important biologically active intermediates for vast applications.

## 3. Materials and Methods

### 3.1. General Procedures

All reagents used were of analytical grade. FTIR spectra were recorded on a FT-IR 4100 spectrometer (JASCO, Tokyo, Japan). NMR spectra were measured by an EX 270 spectrometer (JEOL, Tokyo, Japan). Optical rotations were measured at 23 °C on a JASCO DIP370 polarimeter (JASCO, Tokyo, Japan). HRMS-ESI spectra were obtained with a Waters UPLC ESI-TOF mass spectrometer (Waters, Milford, CT, USA).

### 3.2. General Procedure for the Preparation of TFA-α-Amino Acid

The procedure was based on that of previous studies [19,20] with slight modifications. The corresponding amino groups of α-amino acid underwent TFA protection by using ethyl trifluoroacetate in the presence of triethylamine in methanol to generate TFA-α-amino acid (l-/d-**1a**–l-/d-**2a**, **3a**, l-/d-**4a**–l-/d-**8a**; see Supplementary Materials Scheme SM-1).

### 3.3. General Procedure for the Preparation of TFA-α-Amino Acid N-Hydroxysuccinimide Ester

NHS (*N*-hydroxysuccinimide, 1.1 equiv.) was added to a solution of TFA-α-amino acid (l-/d-**1a**–l-/d-**2a**, **3a**, l-/d-**4a**–l-/d-**8a**, 1.0 mmol) in pre-cooled CH_2_Cl_2_ (10 mL). The suspension of WSCD-HCl (water soluble carbodiimide hydrochloride, 1-ethyl-3-(3-dimethylaminopropyl)carbodiimide monohydrochloride, 1.0 equiv.) in CH_2_Cl_2_ (10 mL) was added drop-wise at 0 °C and the reaction was stirred for 2–4 h. The solvent was removed by rotary evaporation. The residue that remained was dissolved in ethyl acetate; washed with water, sat. NaHCO_3_, sat. NaCl, dried over MgSO_4_, and then evaporated. The product was solidified by washing with hexane to be used for further reaction.

*(2S,3S)-2,5-Dioxopyrrolidin-1-yl 3-methyl-2-(2,2,2-trifluoroacetamido)pentanoate* (**TFA-l-Ile-OSu**, **l-1b**) [24]: Colorless amorphous mass. [α]_D_ = −4.0 (*c* 1.0, CHCl_3_). IR (neat) *ν*: 3265, 2965, 1785, 1740 cm^−1^. ^1^H-NMR (270 MHz, CDCl_3_) δ: 7.41 (d, *J* = 8.6 Hz, 1H, N*H*), 4.97 (dd, *J* = 8.6, 5.3 Hz, 1H, C*H*NH), 2.89 (s, 4H, 2 × CH_2_), 2.23–2.08 (m, 1H, C*H*CH_3_), 1.75–1.60 (1H, m, C*H*_2_CH_3_), 1.45–1.29 (1H, m, CH_2_CH_3_), 1.11 (d, *J* = 6.9 Hz, 3H, CHC*H*_3_), 1.02 (t, *J* = 7.4 Hz, 3H, CH_2_C*H*_3_) ppm. ^13^C-NMR (67.5 MHz, CDCl_3_) δ: 168.5 (2 × CO), 166.3, 156.9 (q, ^2^*J_CF_* = 38.4 Hz), 115.5 (q, ^1^*J_CF_* = 287.9 Hz), 55.3, 38.0, 25.5 (2 × CH_2_), 24.7, 14.8, 11.3 ppm. HRMS-ESI (*m*/*z*) [M + Na]^+^ calcd for C_12_H_15_F_3_N_2_O_5_Na 347.0831, found 347.0822.

*(2R,3R)-2,5-Dioxopyrrolidin-1-yl 3-methyl-2-(2,2,2-trifluoroacetamido)pentanoate* (**TFA**-**d**-**Ile**-**OSu**, **d-1b**): Colorless amorphous mass. [α]_D_ = +4.0 (*c* 1.0, CHCl_3_). IR (neat) *ν*: 3285, 2968, 1789, 1731 cm^−1^. ^1^H-NMR (270 MHz, CDCl_3_) δ: 6.97 (d, *J* = 8.2 Hz, 1H, N*H*), 4.97 (dd, *J* = 8.6, 4.9 Hz, 1H, C*H*NH), 2.86 (s, 4H, 2 × CH_2_) 2.20–2.05 (m, 1H, C*H*CH_3_), 1.71–1.56 (m, 1H, C*H*_2_CH_3_), 1.41–1.21 (m, 1H, C*H*_2_CH_3_), 1.07 (d, *J* = 6.9 Hz, 3H, CHC*H*_3_), 1.00 (t, *J* = 7.4 Hz, 3H, CH_2_C*H*_3_) ppm. ^13^C NMR (67.5 MHz, CDCl_3_) δ: 168.7 (2 × CO), 166.2, 156.9 (q, ^2^*J_CF_* = 38.2 Hz), 115.5 (q, ^1^*J_CF_* = 287.5 Hz), 55.3, 37.7, 25.4 (2 × CH_2_), 24.6, 14.7, 11.1 ppm. HRMS-ESI (*m*/*z*) [M + Na]^+^ calcd for C_12_H_15_F_3_N_2_O_5_Na 347.0831, found 347.0833.

*(2S,3R)-2,5-Dioxopyrrolidin-1-yl 3-methyl-2-(2,2,2-trifluoroacetamido)pentanoate* (**TFA**-**l**-***allo***-**Ile**-**OSu**, **l-2b**): Colorless amorphous mass. [α]_D_ = −4.0 (*c* 1.0, CHCl_3_). IR (neat) *ν*: 3316, 2929, 1788, 1752 cm^−1^. ^1^H-NMR (270 MHz, CDCl_3_) δ: 7.06 (d, *J* = 9.2 Hz, 1H, N*H*), 5.10 (dd, *J* = 9.2, 4.0 Hz, 1H, C*H*NH), 2.86 (s, 4H, 2 × CH_2_), 2.31–2.15 (m, 1H, C*H*CH_3_), 1.54–1.41 (m, 1H, C*H*_2_CH_3_), 1.37–1.23 (m, 1H, C*H*_2_CH_3_), 1.05 (d, *J* = 6.9 Hz, 3H, CHC*H*_3_), 1.00 (t, *J* = 7.4 Hz, 3H, CH_2_C*H*_3_) ppm. ^13^C-NMR (67.5 MHz, CDCl_3_) δ: 168.4 (2 × CO), 166.8, 157.0 (q, ^2^*J_CF_* = 38.2 Hz), 115.5 (q, ^1^*J_CF_* = 287.7 Hz), 54.1, 38.2, 25.9, 25.5 (2 × CH_2_), 14.1, 11.6 ppm. HRMS-ESI (*m*/*z*) [M + H]^+^ calcd for C_12_H_16_F_3_N_2_O_5_ 325.1011, found 325.1013.

*(2R,3S)-2,5-Dioxopyrrolidin-1-yl 3-methyl-2-(2,2,2-trifluoroacetamido)pentanoate* (**TFA**-**d**-***allo***-**Ile**-**OSu**, **d**-**2b**): Colorless amorphous mass. [α]_D_ = +4.0 (*c* 1.0, CHCl_3_). IR (neat) *ν*: 3327, 2971, 1752 cm^−1^. ^1^H-NMR (270 MHz, CDCl_3_) δ: 7.07 (d, *J* = 9.2 Hz, 1H, N*H*), 5.10 (dd, *J* = 9.1, 4.1 Hz, 1H, C*H*NH), 2.86 (s, 4H, 2 × CH_2_), 2.28–2.17 (m, 1H, C*H*CH_3_), 1.57–1.39 (m, 1H, C*H*_2_CH_3_), 1.37–1.22 (m, 1H, C*H*_2_CH_3_), 1.05 (d, *J* = 6.9 Hz, 3H, CHC*H*_3_), 1.00 (t, *J* = 7.4 Hz, 3H, CH_2_C*H*_3_) ppm. ^13^C-NMR (67.5 MHz, CDCl_3_) δ: 168.7 (2 × CO), 166.7, 157.1 (q, ^2^*J_CF_* = 38.2 Hz), 115.5 (q, ^1^*J_CF_* = 287.3 Hz), 54.1, 37.9, 25.8, 25.4 (2 × CH_2_), 14.0, 11.4 ppm. HRMS-ESI (*m*/*z*) [M + H]^+^ calcd for C_12_H_16_F_3_N_2_O_5_ 325.1011, found 325.1014.

*2,5-Dioxocyclopentyl 2-(2,2,2-trifluoroacetamido)acetate* (**TFA**-**Gly**-**OSu**, **3b**) [25]: Colorless amorphous mass. IR (neat) *ν*: 3313, 2998, 1690 cm^−1^. ^1^H-NMR (270 MHz, CD_3_OD) δ: 4.44 (s, 2H, C*H*_2_NH), 2.84 (s, 4H, 2 × CH_2_) ppm. ^13^C NMR (67.5 MHz, ACETONE-*d*_6_) δ: 170.1 (2 × CO), 165.7 158.2 (q, ^2^*J_CF_* = 37.4 Hz), 116.8 (q, ^1^*J_CF_* = 287.0 Hz), 39.5, 26.2 (2 × CH_2_) ppm. HRMS-ESI (*m*/*z*) [M + H]^+^ calcd for C_8_H_7_F_3_N_2_O_5_Na 291.0205, found 291.0208.

*(S)-2,5-Dioxopyrrolidin-1-yl 2-(2,2,2-trifluoroacetamido)propanoate* (**TFA**-**l**-**Ala**-**OSu**, **l-4b**): Colorless amorphous mass. [α]_D_ = −46 (*c* 1.0, CHCl_3_). IR (neat) *ν*: 3332, 2999, 1793, 1734 cm^−1^. ^1^H-NMR (270 MHz, CDCl_3_) δ: 7.39 (d, *J* = 7.3 Hz, 1H, N*H*), 5.08–4.97 (m, 1H, C*H*CH_3_), 2.86 (s, 4H, 2 × C*H*_2_), 1.68 (d, *J* = 7.3 Hz, 3H, CHC*H*_3_) ppm. ^13^C NMR (67.5 MHz, CDCl_3_) δ: 168.8 (2 × CO), 167.4, 156.8 (q, ^2^*J_CF_* = 38.4 Hz), 115.6 (q, ^1^*J_CF_* = 287.2 Hz), 46.7, 25.5 (2 × CH_2_), 17.6 ppm. HRMS-ESI (*m*/*z*) [M + H]^+^ calcd for C_9_H_10_F_3_N_2_O_5_ 283.0542, found 283.0555.

*(R)-2,5-Dioxopyrrolidin-1-yl 2-(2,2,2-trifluoroacetamido)propanoate* (**TFA**-**d**-**Ala**-**OSu**, **d**-**4b**): Colorless amorphous mass. [α]_D_ = +46 (*c* 1.0, CHCl_3_). IR (neat) *ν*: 3350, 2999, 1798, 1726 cm^−1^. ^1^H-NMR (270 MHz, CDCl_3_) δ: 7.73 (d, *J* = 7.6 Hz, 1H, N*H*), 5.05–4.94 (m, 1H, C*H*CH_3_), 2.85 (s, 4H, 2 × C*H*_2_), 1.66 (d, *J* = 7.3 Hz, 3H, CHC*H*_3_) ppm.^13^C NMR (67.5 MHz, CDCl_3_) δ: 169.0 (2 × CO), 167.1, 156.8 (q, ^2^*J_CF_* = 38.2 Hz), 115.4 (q, ^1^*J_CF_* = 287.3 Hz), 46.7, 25.4 (2 × CH_2_), 17.1 ppm. HRMS-ESI (*m*/*z*) [M + H]^+^ calcd for C_9_H_10_F_3_N_2_O_5_ 283.0542, found 283.0553.

*(S)-2,5-Dioxopyrrolidin-1-yl 3-methyl-2-(2,2,2-trifluoroacetamido)butanoate* (**TFA**-**l**-**Val**-**OSu**, **l-5b**) [24]: Colorless amorphous mass. [α]_D_ = −22 (*c* 1.0, CHCl_3_). IR (neat) *ν*: 3310, 2979, 1796, 1725 cm^−1^. ^1^H-NMR (270 MHz, CD_3_Cl_3_) δ: 7.49 (d, *J* = 8.9 Hz, 1H, N*H*), 4.88 (dd, *J* = 8.7, 5.4 Hz, 1H, C*H*NH), 2.84 (s, 4H, 2 × CH_2_), 2.47–2.34 (m, 1H, C*H*CH_3_), 1.08 (d, *J* = 6.9 Hz, 6H, 2 × CH_3_) ppm. ^13^C-NMR (67.5 MHz, CDCl_3_) δ: 168.9 (2 × CO), 166.1, 157.1 (q, ^2^*J_CF_* = 38.2 Hz), 115.5 (q, ^1^*J_CF_* = 287.5 H), 55.9, 31.2, 25.4 (2 × CH_2_), 18.3, 17.2 ppm. HRMS-ESI (*m*/*z*) [M + H]^+^ calcd for C_11_H_14_F_3_N_2_O_5_ 311.0855, found 311.0858.

*(R)-2,5-Dioxopyrrolidin-1-yl 3-methyl-2-(2,2,2-trifluoroacetamido)butanoate* (**TFA**-**d**-**Val**-**OSu**, **d**-**5b**): Colorless amorphous mass. [α]_D_ = +22 (*c* 1.0, CHCl_3_). IR (neat) *ν*: 3298, 2975, 1725 cm^−1^. ^1^H-NMR (270 MHz, CD_3_Cl_3_) δ: 6.93 (d, *J* = 8.6 Hz, 1H, N*H*), 4.95 (dd, *J* = 8.9, 4.9 Hz, 1H, C*H*NH), 2.87 (s, 4H, 2 × CH_2_), 2.53–2.35 (m, 1H, C*H*CH_3_), 1.09 (d, *J* = 6.9 Hz, 6H, 2 × CH_3_) ppm. ^13^C-NMR (67.5 MHz, CDCl_3_) δ: 168.4 (2 × CO), 166.4, 157.0 (q, ^2^*J_CF_* = 38.0 Hz), 115.5 (q, ^1^*J_CF_* = 287.7 Hz), 55.9, 31.7, 25.5 (2 × CH_2_), 18.4, 17.2 ppm. HRMS-ESI (*m*/*z*) [M + H]^+^ calcd for C_11_H_14_F_3_N_2_O_5_ 311.0855, found 311.0857.

*(S)-2,5-Dioxopyrrolidin-1-yl 4-methyl-2-(2,2,2-trifluoroacetamido)pentanoate* (**TFA**-**l**-**Leu**-**OSu**, **l-6b**) [24]: Colorless amorphous mass. [α]_D_ = −42 (*c* 1.0, CHCl_3_). IR (neat) *ν*: 3295, 2967, 1734, 1712 cm^−1^. ^1^H-NMR (270 MHz, CD_3_Cl_3_) δ: 6.86 (d, *J* = 8.6 Hz, 1H, N*H*), 5.04 (td, *J* = 8.9, 4.9 Hz, 1H, C*H*NH), 2.86 (s, 4H 2 × CH_2_), 2.00–1.72 (m, 3H, C*H*_2_C*H*), 1.02 (d, *J* = 2.3 Hz, 3H, CH_3_), 1.00 (d, *J* = 2.3 Hz, 3H, CH_3_) ppm. ^13^C-NMR (67.5 MHz, CDCl_3_) δ 168.6 (2 × CO), 167.2, 156.9 (q, ^2^*J_CF_* = 38.2 Hz), 115.5 (q, ^1^*J_CF_* = 287.7 Hz), 49.3, 41.0, 25.5 (2 × CH_2_), 24.7, 22.6, 21.5 ppm. HRMS-ESI (*m*/*z*) [M + Na]^+^ calcd for C_12_H_15_F_3_N_2_O_5_Na 347.0831, found 347.0832.

*(R)-2,5-Dioxopyrrolidin-1-yl 4-methyl-2-(2,2,2-trifluoroacetamido)pentanoate* (**TFA**-**d**-**Leu**-**OSu**, **d**-**6b**): Colorless amorphous mass. [α]_D_ = +42 (*c* 1.0, CHCl_3_). IR (neat) *ν*: 3305, 2965, 1733, 1719 cm^−1^. ^1^H-NMR (270 MHz, CD_3_Cl_3_) δ: 6.97 (br s, 1H, N*H*), 5.05 (td, *J* = 8.7, 4.9 Hz, 1H), 2.86 (s, 4H, 2 × CH_2_), 2.00–1.74 (m, 3H, C*H*_2_C*H*), 1.03–0.98 (m, 6H, 2 × CH_3_) ppm. ^13^C-NMR (67.5 MHz, CDCl_3_) δ: 168.6 (2 × CO), 167.2, 156.9 (q, ^2^*J_CF_* = 38.2 Hz), 115.5 (q, ^1^*J_CF_* = 287.7 Hz), 49.3, 41.0, 25.5 (2 × CH_2_), 24.7, 22.6, 21.5 ppm. HRMS-ESI (*m*/*z*) [M + Na]^+^ calcd for C_12_H_15_F_3_N_2_O_5_Na 347.0831, found 347.0830.

*(S)-2,5-Dioxopyrrolidin-1-yl 2-(2,2,2-trifluoroacetamido)pentanoate* (**TFA**-**l**-**Nva**-**OSu**, **l-7b**): Colorless amorphous mass. [α]_D_ = −27 (*c* 1.0, CHCl_3_). IR (neat) *ν*: 3325, 2962, 1744, 1711 cm^−1^. ^1^H-NMR (270 MHz, CDCl_3_) δ: 6.83 (d, *J* = 7.3 Hz, 1H, N*H*), 5.03 (td, *J* = 8.0, 5.5 Hz, 1H, C*H*NH), 2.87 (s, 4H, 2 × CH_2_), 2.17–2.02 (m, 1H, CHC*H*_2_), 1.99–1.85 (m, 1H, CHC*H*_2_), 1.59–1.40 (m, 2H, C*H*_2_CH_3_), 1.01 (t, *J* = 7.3 Hz, 3H, CH_2_C*H*_3_) ppm. ^13^C-NMR (67.5 MHz, CDCl_3_) δ: 168.6 (2 × CO), 166.9, 156.9 (q, ^2^*J_CF_* = 38.0 Hz), 115.5 (q, ^1^*J_CF_* = 287.7 Hz), 50.7, 34.0, 25.5 (2 × CH_2_), 18.2, 13.3 ppm. HRMS-ESI (*m*/*z*) [M + H]^+^ calcd for C_11_H_14_F_3_N_2_O_5_ 311.0855, found 311.0856.

*(R)-2,5-Dioxopyrrolidin-1-yl 2-(2,2,2-trifluoroacetamido)pentanoate* (**TFA**-**d**-**Nva**-**OSu**, **d**-**7b**): Colorless amorphous mass. [α]_D_ = +27 (*c* 1.0, CHCl_3_). IR (neat) *ν*: 3322, 2962, 1744, 1711 cm^−1^. ^1^H-NMR (270 MHz, CDCl_3_) δ: 7.11 (d, *J* = 8.2 Hz, 1H, N*H*), 5.02 (td, *J* = 8.1, 5.4 Hz, 1H, C*H*NH), 2.86 (s, 4H, 2 × CH_2_), 2.15–2.01 (m, 1H, CHC*H*_2_), 1.98–1.84 (m, 1H, CHC*H*_2_), 1.60–1.43 (m, 2H, C*H*_2_CH_3_), 1.00 (t, *J* = 7.3 Hz, 3H, CH_2_C*H*_3_) ppm. ^13^C-NMR (67.5 MHz, CDCl_3_) δ: 168.6 (2 × CO), 166.9, 156.9 (q, ^2^*J_CF_* = 38.2 Hz), 115.5 (q, ^1^*J_CF_* = 287.9 Hz), 50.7, 34.0, 25.5 (2 × CH_2_), 18.2, 13.3 ppm. HRMS-ESI (*m*/*z*) [M + H]^+^ calcd for C_11_H_14_F_3_N_2_O_5_ 311.0855, found 311.0861.

*(S)-2,5-Dioxopyrrolidin-1-yl 2-(2,2,2-trifluoroacetamido)hexanoate* (**TFA**-**l**-**Nle**-**OSu**, **l-8b**): Colorless amorphous mass. [α]_D_ = −18 (*c* 1.0, CHCl_3_). IR (neat) *ν*: 3332, 2958, 1721, 1703 cm^−1^. ^1^H-NMR (270 MHz, CDCl_3_) δ: 7.34 (d, *J* = 8.2 Hz, 1H, N*H*), 4.98 (td, *J* = 8.2, 5.3 Hz, 1H, C*H*NH), 2.85 (s, 4H, 2 × CH_2_), 2.16–2.03 (m, 1H, CHC*H*_2_), 1.99–1.85 (m, 1H, CHC*H*_2_), 1.52–1.32 (m, 4H, 2 × CH_2_), 0.93 (t, *J* = 7.1 Hz, 3H, CH_2_C*H*_3_) ppm. ^13^C-NMR (67.5 MHz, CDCl_3_) δ: 168.8 (2 × CO), 166.8, 156.9 (q, ^2^*J_CF_* = 38.2 Hz), 115.5 (q, ^1^*J_CF_* = 287.7 Hz), 50.8, 31.5, 26.9, 25.4 (2 × CH_2_), 21.9, 13.5 ppm. HRMS-ESI (*m*/*z*) [M + H]^+^ calcd for C_12_H_16_F_3_N_2_O_5_ 325.1011, found 325.1021.

*(R)-2,5-Dioxopyrrolidin-1-yl 2-(2,2,2-trifluoroacetamido)hexanoate* (**TFA**-**d**-**Nle**-**OSu**, **d**-**8b**): Colorless amorphous mass. [α]_D_ = +18 (*c* 1.0, CHCl_3_). IR (neat) *ν*: 3324, 2958, 1724, 1708 cm^−1^. ^1^H-NMR (270 MHz, CDCl_3_) δ: 6.89 (br s, 1H, N*H*), 5.01 (td, *J* = 7.8, 5.5 Hz, 1H, C*H*NH), 2.87 (s, 4H, 2 × CH_2_), 2.18–2.04 (m, 1H, CHC*H*_2_), 2.00–1.86 (m, 1H, CHC*H*_2_), 1.52–1.36 (m, 4H, 2 × C*H*_2_), 0.94 (t, *J* = 7.1 Hz, 3H, CH_2_C*H*_3_) ppm. ^13^C-NMR (67.5 MHz, CDCl_3_) δ: 168.6 (2 × CO), 166.9, 156.9 (q, ^2^*J_CF_* = 38.4 Hz), 115.5 (q, ^1^*J_CF_* = 287.3 Hz), 50.8, 31.7, 26.8, 25.5 (2 × CH_2_), 21.9, 13.6 ppm. HRMS-ESI (*m*/*z*) [M + H]^+^ calcd for C_12_H_16_F_3_N_2_O_5_ 325.1011, found 325.1016.

### 3.4. General Procedure for the Preparation of TFA-Protected α-Amino Aryl-Ketones

TFA-α-amino acid-OSu (l-/d-**1b**–l-/d-**2b**, **3b**, l-/d-**4b**–l-/d-**8b**, 0.9–1.6 mmol) was suspended in arene. Into the suspension, pulverized AlCl_3_ (6 equiv.) was added and then stirred at a temperature of 70 °C. The reaction was monitored by the consumption of starting material on TLC. Then, the mixture was poured into an ethyl acetate-H_2_O two-phase system to quench the reaction. The organic layer was washed with H_2_O, sat. NaCl, dried over MgSO_4_, and then evaporated. The crude product was purified by silica column chromatography (ethyl acetate/hexane 1:3 l-/d-**1c**, l-/d-**2c**, **3c**, and l-/d-**4b**–l-/d-**8b**; and diethyl ether/hexane 1:6 l-/d-**1d**–l-/d-**1h**).

*2,2,2-Trifluoro-N-((2S,3S)-3-methyl-1-oxo-1-phenylpentan-2-yl)acetamide* (**TFA-l-Ile-Ph**, **l-1c**): Colorless needles. [α]_D_ = +70 (*c* 2.0, CHCl_3_). IR (neat) *ν*: 3317, 3073, 2972, 1722, 1694 cm^−1^. ^1^H-NMR (270 MHz, CDCl_3_) δ: 7.97 (d, *J* = 7.4 Hz, 2H, Ar-H), 7.66 (t, *J* = 7.4 Hz, 1H, Ar-H), 7.53 (t, *J* = 7.4 Hz, 2H, Ar-H), 5.60 (dd, *J* = 8.6, 4.3 Hz, 1H, C*H*NH), 2.10–1.95 (m, 1H, C*H*CH_3_), 11.40–1.25 (m, 1H, C*H*_2_CH_3_), 1.12–0.95 (m, 4H, overlap C*H*_2_CH_3_ and CHC*H*_3_), 0.82 (t, *J* = 7.3 Hz, 3H, CH_2_C*H*_3_) ppm. ^13^C-NMR (67.5 MHz, CDCl_3_) δ: 197.5, 157.1 (q, ^2^*J_CF_* = 37.4 Hz), 134.7, 134.3, 129.0 (2 × CH), 128.7 (2 × CH), 115.9 (q, ^1^*J_CF_* = 288.1 Hz), 58.4, 38.7, 23.7, 16.2, 11.4 ppm. HRMS-ESI (*m*/*z*) [M + H]^+^ calcd for C_14_H_17_F_3_NO_2_ 288.1211, found 288.1212.

*2,2,2-Trifluoro-N-((2R,3R)-3-methyl-1-oxo-1-phenylpentan-2-yl)acetamide* (**TFA**-**d**-**Ile**-**Ph**, **d**-**1c**): Colorless needles. [α]_D_ = −70 (*c* 2.0, CHCl_3_). IR (neat) *ν*: 3337, 3069, 2969, 1721, 1699 cm^−1^. ^1^H-NMR (270 MHz, CDCl_3_) δ: 7.98 (d, *J* = 7.6 Hz, 2H, Ar-H), 7.66 (t, *J* = 7.6 Hz, 1H, Ar-H), 7.53 (t, *J* = 7.6 Hz, 2H, Ar-H), 5.60 (dd, *J* = 8.6, 4.0 Hz, 1H, C*H*NH), 2.10–1.95 (m, 1H, C*H*CH_3_), 1.40–1.23 (m, 1H, C*H*_2_CH_3_), 1.12–0.95 (m, 4H, overlap C*H*_2_CH_3_ and CHC*H*_3_), 0.82 (t, *J* = 7.3 Hz, 3H, CH_2_C*H*_3_) ppm.^13^C-NMR (67.5 MHz, CDCl_3_) δ: 197.5, 157.1 (q, ^2^*J_CF_* = 37.2 Hz), 134.7, 134.3, 129.0 (2 × CH), 128.7 (2 × CH), 115.9 (q, ^1^*J_CF_* = 288.3 Hz), 58.4, 38.7, 23.7, 16.2, 11.4 ppm. HRMS-ESI (*m*/*z*) [M + H]^+^ calcd for C_14_H_17_F_3_NO_2_ 288.1211, found 288.1215.

*2,2,2-Trifluoro-N-((2S,3S)-3-methyl-1-oxo-1-(p-tolyl)pentan-2-yl)acetamide* (**TFA**-**l**-**Ile**-**Ph(4**-**Me)**, **l-1d**): Colorless needles. [α]_D_ = +83 (*c* 1.0, CHCl_3_). IR (neat) *ν*: 302, 3023, 2925, 1700 cm^−1^. ^1^H-NMR (270 MHz, CDCl_3_) δ: 7.87 (d, *J* = 8.2 Hz, 2H, Ar-H), 7.32 (d, *J* = 8.6 Hz, 2H, Ar-H), 5.56 (dd, *J* = 8.6, 4.3 Hz, 1H, C*H*NH), 2.45 (s, 3H, C*H*_3_), 2.06–1.95 (m, 1H, C*H*CH_3_), 1.39–1.26 (m, 1H, C*H*_2_CH_3_), 1.11–0.94 (m, 4H, overlap C*H*_2_CH_3_ and CHC*H*_3_), 0.82 (t, *J* = 7.3 Hz, 3H, CH_2_C*H*_3_) ppm. ^13^C-NMR (67.5 MHz, CDCl_3_) δ: 197.0, 157.1 (q, ^2^*J_CF_* = 37.2 Hz), 145.5, 132.1 (2 × CH), 129.7 (2 × CH), 128.8, 115.9 (q, ^1^*J_CF_* = 287.9 Hz), 58.3, 38.9, 23.7, 21.8, 16.2, 11.4 ppm. HRMS-ESI (*m*/*z*) [M + H]^+^ calcd for C_15_H_19_F_3_NO_2_ 302.1368 found 302.1353.

*2,2,2-Trifluoro-N-((2S,3S)-1-(4-methoxyphenyl)-3-methyl-1-oxopentan-2-yl)acetamide* (**TFA**-**l**-**Ile**-**Ph**(**4**-**OMe**), **l**-**1e**): Colorless oil. [α]_D_ = +66 (*c* 1.0, CHCl_3_). IR (neat) *ν*: 3320, 3079, 2934, 1726, 1675 cm^−1^. ^1^H-NMR (270 MHz, CDCl_3_) δ: 7.96 (d, *J* = 8.9 Hz, 2H, Ar-H), 6.99 (d, *J* = 8.9 Hz, 2H, Ar-H), 5.53 (dd, *J* = 8.7, 4.5 Hz, 1H, C*H*NH), 3.90 (s, 3H, OC*H*_3_), 2.08–1.93 (m, 1H, C*H*CH_3_), 1.42-1.20 (m, 1H, C*H*_2_CH_3_), 1.10–0.95 (m, 4H, overlap C*H*_2_CH_3_ and CHC*H*_3_), 0.82 (t, *J* = 7.4 Hz, 3H, CH_2_C*H*_3_) ppm. ^13^C-NMR (67.5 MHz, CDCl_3_) δ: 195.6, 164.5, 157.0 (q, ^2^*J_CF_* = 37.2 Hz), 131.1 (2 × CH), 127.5, 115.9 (q, ^1^*J_CF_* = 288.6 Hz), 114.2 (2 × CH), 58.0, 55.6, 39.0, 23.7, 16.2, 11.4 ppm. HRMS-ESI (*m*/*z*) [M + H]^+^ calcd for C_15_H_18_F_3_NO_3_Na 340.1136 found 340.1145.

*N-((2S,3S)-1-(3,4-Dimethylphenyl)-3-methyl-1-oxopentan-2-yl)-2,2,2-trifluoroacetamide* (**TFA**-**l**-**Ile**-**Ph(3,4**-**Me)**, **l**-**1f**): Colorless needles. [α]_D_ = +82 (*c* 1.0, CHCl_3_). IR (neat) *ν*: 3343, 3075, 2979, 1742, 1691 cm^−1^. ^1^H-NMR (270 MHz, CDCl_3_) δ: 7.71 (d, *J* = 11.2 Hz, 2H, Ar-H), 7.29 (s, 1H, Ar-H), 5.56 (dd, *J* = 8.7, 4.1 Hz, 1H, C*H*NH), 2.35 (s, 6H, 2 × C*H*_3_), 2.06–1.95 (m, 1H, C*H*CH_3_), 1.40–1.26 (m, 1H, C*H*_2_CH_3_), 1.08–0.93 (m, 4H, overlap C*H*_2_CH_3_ and CHC*H*_3_), 0.81 (t, *J* = 7.4 Hz, 3H, CH_2_C*H*_3_) ppm. ^13^C-NMR (67.5 MHz, CDCl_3_) δ: 197.2, 157.1 (q, ^2^*J_CF_* = 37.2 Hz), 144.3, 137.6, 132.5, 130.2, 129.7, 126.5, 115.9 (q, ^1^*J_CF_* = 287.9 Hz), 58.3, 38.9, 23.7, 20.1, 19.8, 16.2, 11.4 ppm. HRMS-ESI (*m*/*z*) [M + H]^+^ calcd for C_16_H_21_F_3_NO_2_ 316.1524, found 316.1506.

*N-((2S,3S)-1-(2,4-Dimethylphenyl)-3-methyl-1-oxopentan-2-yl)-2,2,2-trifluoroacetamide* (**TFA**-**l**-**Ile**-**Ph(2,4**-**Me)**, **l**-**1g**): Colorless needles. [α]_D_ = +49 (*c* 0.25, CHCl_3_). IR (neat) *ν*: 3294, 3097, 2969, 1714, 1685 cm^−1^. ^1^H-NMR (270 MHz, CDCl_3_) δ: 7.63 (d, *J* = 8.6 Hz, 1H, Ar-H), 7.14-7.02 (m, 2H, Ar-H), 5.49 (dd, *J* = 8.4, 4.1 Hz, 1H, C*H*NH), 2.49 (s, 3H, C*H*_3_), 2.38 (s, 3H, C*H*_3_), 1.99–1.87 (m, 1H, C*H*CH_3_), 1.32–1.19 (m, 1H, C*H*_2_CH_3_), 1.07–0.87 (m, 4H, overlap C*H*_2_CH_3_ and CHC*H*_3_), 0.81 (t, *J* = 7.3 Hz, 3H, CH_2_C*H*_3_) ppm. ^13^C-NMR (67.5 MHz, CDCl_3_) δ: 199.9, 157.1 (q, ^2^*J_CF_* = 37.4 Hz), 143.2, 139.5, 133.3, 132.2, 129.4, 126.6, 115.9 (q, ^1^*J_CF_* = 288.3 Hz), 59.8, 38.5, 24.1, 21.2, 21.0, 15.9, 11.2 ppm. HRMS-ESI (*m*/*z*) [M + H]^+^ calcd for C_16_H_21_F_3_NO_2_ 316.1524, found 316.1526.

*N-((2S,3S)-1-(2,5-Dimethylphenyl)-3-methyl-1-oxopentan-2-yl)-2,2,2-trifluoroacetamide* (**TFA**-**l**-**Ile**-**Ph(2,5**-**Me)**, **l**-**1h**): Colorless needles. [α]_D_ = +68 (*c* 1.0, CHCl_3_). IR (neat) *ν*: 3302, 3024, 2934, 1714, 1686, 1567 cm^−1^. ^1^H-NMR (270 MHz, CDCl_3_) δ: 7.5 (s, 1H, Ar-H), 7.3 (d, *J* = 7.6 Hz, 1H, Ar-H), 7.2 (d, *J* = 7.6 Hz, 1H, Ar-H), 5.5 (dd, *J* = 8.6, 4.0 Hz, 1H, C*H*NH), 2.4 (s, 3H, C*H*_3_), 2.4 (s, 3H, C*H*_3_), 2.0–1.8 (m, 1H, C*H*CH_3_), 1.4–1.2 (m, 1H, C*H*_2_CH_3_), 1.1–0.9 (m, 3H overlap C*H*_2_CH_3_ and CHC*H*_3_), 0.8 (t, *J* = 7.3 Hz, 3H, CH_2_C*H*_3_) ppm. ^13^C-NMR (67.5 MHz, CDCl_3_) δ: 200.7, 157.1 (q, ^2^*J_CF_* = 37.2 Hz), 135.9, 135.6, 135.0, 133.3, 132.3, 129.3, 115.9 (q, ^1^*J_CF_* = 287.9 Hz), 60.1, 38.4, 24.1, 20.7, 20.3, 15.9, 11.3 ppm. HRMS-ESI (*m*/*z*) [M + H]^+^ calcd for C_16_H_21_F_3_NO_2_ 316.1524, found 316.1530.

*2,2,2-Trifluoro-N-((2S,3R)-3-methyl-1-oxo-1-phenylpentan-2-yl)acetamide* (**TFA**-**l**-***allo***-**Ile**-**Ph**, **l**-**2c**): Colorless needles. [α]_D_ = +79 (*c* 2.0, CHCl_3_). IR (neat) *ν*: 3335, 3068, 2971, 1741, 1693 cm^−1^. ^1^H-NMR (270 MHz, CDCl_3_) δ: 7.98 (d, *J* = 7.6 Hz, 2H, Ar-H), 7.65 (t, *J* = 7.6 Hz, 1H, Ar-H), 7.53 (t, *J* = 7.6 Hz, 2H, Ar-H), 5.74 (dd, *J* = 8.9, 3.0 Hz, 1H, C*H*NH), 2.09–1.94 (m, 1H, C*H*CH_3_), 1.62–1.49 (m, 1H, C*H*_2_CH_3_), 1.38–1.21 (m, 1H, C*H*_2_CH_3_), 1.06 (t, *J* = 7.3 Hz, 3H, CH_2_C*H*_3_), 0.76 (d, *J* = 6.9 Hz, 3H, CHC*H*_3_) ppm. ^13^C NMR (67.5 MHz, CDCl_3_) δ: 197.1, 157.3 (q, ^2^*J_CF_* = 37.1 Hz), 134.4, 134.0, 129.1 (2 × CH), 128.7 (2 × CH), 115.9 (q, ^1^*J_CF_* = 287.7 Hz), 57.1, 38.7, 27.3, 13.4, 12.0 ppm. HRMS-ESI (*m*/*z*) [M + Na]^+^ calcd for C_14_H_16_F_3_NO_2_Na 310.1031, found 310.1039.

*2,2,2-Trifluoro-N-((2R,3S)-3-methyl-1-oxo-1-phenylpentan-2-yl)acetamide* (**TFA**-**d**-***allo***-**Ile**-**Ph**, **d**-**2c**): Colorless needles. [α]_D_ = −79 (*c* 2.0, CHCl_3_). IR (neat) *ν*: 3331, 3067, 2969, 1738, 1692 cm^−1^. ^1^H-NMR (270 MHz, CDCl_3_) δ: 7.98 (d, *J* = 7.4 Hz, 2H, Ar-H), 7.66 (t, *J* = 7.4 Hz, 1H, Ar-H), 7.53 (t, *J* = 7.4 Hz, 2H, Ar-H), 5.74 (dd, *J* = 8.7, 2.8 Hz, 1H, C*H*NH), 2.07–1.94 (m, 1H, C*H*CH_3_), 1.66–1.48 (m, 1H, C*H*_2_CH_3_), 1.38–1.21 (m, 1H, C*H*_2_CH_3_), 1.06 (t, *J* = 7.3 Hz, 3H, CH_2_C*H*_3_), 0.76 (d, *J* = 6.9 Hz, 3H, CHC*H*_3_) ppm. ^13^C-NMR (67.5 MHz, CDCl_3_) δ: 197.1, 157.3 (q, ^2^*J_CF_* = 37.4 Hz), 134.4, 134.1, 129.1 (2 × CH), 128.7 (2 × CH), 115.9 (q, ^1^*J_CF_* = 287.9 Hz), 57.1, 38.7, 27.3, 13.4, 12.0 ppm. HRMS-ESI (*m*/*z*) [M + Na]^+^ calcd for C_14_H_16_F_3_NO_2_Na 310.1031, found 310.1040.

*2,2,2-Trifluoro-N-(2-oxo-2-phenylethyl)acetamide* (**TFA**-**Gly**-**Ph**, **3c**) [2]: Colorless oil. IR (neat) *ν*: 3327, 3103, 2927, 1733, 1703 cm^−1^. ^1^H-NMR (270 MHz, CDCl_3_) δ: 7.99 (d, *J* = 7.4 Hz, 2H, Ar-H), 7.68 (t, *J* = 7.4 Hz, 1H, Ar-H), 7.54 (t, *J* = 7.4 Hz, 2H, Ar-H), 4.83 (d, *J* = 4.3 Hz, 2H, C*H*_2_NH) ppm. ^13^C NMR (67.5 MHz, CDCl_3_) δ: 192.1, 157.1 (q, ^2^*J_CF_* = 37.6 Hz), 134.6, 133.6, 129.1 (2 × CH), 127.9 (2 × CH), 115.7 (q, ^1^*J_CF_* = 287.3 Hz), 46.1 ppm. HRMS-ESI (*m*/*z*) [M + H]^+^ calcd for C_10_H_9_F_3_NO_2_ 232.0585, found 232.0595.

*(S)-2,2,2-trifluoro-N-(1-oxo-1-phenylpropan-2-yl)acetamide* (**TFA**-**l**-**Ala**-**Ph**, **l**-**4c**) [7,12,26]: Colorless oil. = −7.0 (*c* 1.0, CHCl_3_). Lit. [26] [α]_D_ = −8.6 (*c* 0.17, CHCl_3_). IR (neat) *ν*: 3331, 3070, 2991, 1738, 1701 cm^−1^. ^1^H-NMR (270 MHz, CDCl_3_) δ: 7.99 (d, *J* = 7.4 Hz, 2H, Ar-H), 7.67 (t, *J* = 7.4 Hz, 1H, Ar-H), 7.54 (t, *J* = 7.4 Hz, 2H, Ar-H), 5.60–5.50 (m, 1H, C*H*CH_3_), 1.53 (d, *J* = 7.3 Hz, 3H, CHC*H*_3_) ppm.^13^C NMR (67.5 MHz, CDCl_3_) δ: 197.0, 156.5 (q, ^2^*J_CF_* = 37.6 Hz), 134.5, 132.9, 129.1 (2 × CH), 128.8 (2 × CH), 115.7 (q, ^1^*J_CF_* = 287.3 Hz), 50.8, 19.2 ppm. HRMS-ESI (*m*/*z*) [M + H]^+^ calcd for C_11_H_11_F_3_NO_2_ 246.0742, found 246.0748.

*(R)-2,2,2-Trifluoro-N-(1-oxo-1-phenylpropan-2-yl)acetamide* (**TFA**-**d**-**Ala**-**Ph**, **d**-**4c**): Colorless oil. [α]_D_ = +7.0 (*c* 1.0, CHCl_3_). IR (neat) *ν*: 3337, 3091, 2948, 1725, 1700 cm^−1^. ^1^H-NMR (270 MHz, CDCl_3_) δ: 7.99 (d, *J* = 7.3 Hz, 2H, Ar-H), 7.67 (t, *J* = 7.4 Hz, 1H, Ar-H), 7.54 (t, *J* = 7.4 Hz, 2H, Ar-H), 5.60–5.49 (m, 1H, C*H*CH_3_), 1.53 (d, *J* = 6.9 Hz, 3H, CHC*H*_3_) ppm. ^13^C NMR (67.5 MHz, CDCl_3_) δ: 197.0, 156.5 (q, ^2^*J_CF_* = 37.6 Hz), 134.5, 132.9, 129.1 (2 × CH), 128.8 (2 × CH), 115.7 (q, ^1^*J_CF_* = 287.7 Hz), 50.8, 19.2 ppm. HRMS-ESI (*m*/*z*) [M + H]^+^ calcd for C_11_H_11_F_3_NO_2_ 246.0742, found 246.0750.

*(S)-2,2,2-Trifluoro-N-(3-methyl-1-oxo-1-phenylbutan-2-yl)acetamide* (**TFA**-**l**-**Val**-**Ph**, **l**-**5c**) [7]: Colorless oil. [α]_D_ = +83 (*c* 1.0, CHCl_3_). IR (neat) *ν*: 3347, 3071, 2972, 1728, 1679 cm^−1^. (270 MHz, CDCl_3_) δ: 7.99 (d, *J* = 7.7 Hz, 2H, Ar-H), 7.66 (t, *J* = 7.7 Hz, 1H, Ar-H), 7.53 (t, *J* = 7.7 Hz, 2H, Ar-H), 7.32 (br s, 1H, N*H*), 5.61 (dd, *J* = 8.6, 4.0 Hz, 1H, C*H*NH), 2.39–2.23 (m, 1H, C*H*CH_3_), 1.07 (d, *J* = 6.9 Hz, 3H, CHC*H*_3_), 0.80 (d, *J* = 6.9 Hz, 3H, CHC*H*_3_) ppm. ^13^C-NMR (67.5 MHz, CDCl_3_) δ: 197.2, 157.3 (q, ^2^*J_CF_* = 37.6 Hz), 134.4, 129.1 (2 × CH), 128.7 (2 × CH), 115.9 (q, ^1^*J_CF_* = 287.7 Hz), 58.6, 32.2, 20.0, 16.4 ppm. HRMS-ESI (*m*/*z*) [M + H]^+^ calcd for C_13_H_15_F_3_NO_2_ 274.1055, found 274.1057.

*(R)-2,2,2-Trifluoro-N-(3-methyl-1-oxo-1-phenylbutan-2-yl)acetamide* (**TFA**-**d**-**Val**-**Ph**, **d**-**5c**): Colorless oil. [α]_D_ = −83 (*c* 1.0, CHCl_3_). IR (neat) *ν*: 3344, 3070, 2972, 1720, 1672 cm^−1^. ^1^H-NMR (270 MHz, CDCl_3_) δ: 7.99 (d, *J* = 7.3 Hz, 2H, Ar-H), 7.66 (t, *J* = 7.3 Hz, 1H, Ar-H), 7.53 (t, *J* = 7.3 Hz, 2H, Ar-H), 7.30 (br s, 1H, N*H*), 5.61 (dd, *J* = 8.7, 3.8 Hz, 1H, C*H*NH), 2.39–2.22 (m, 1H, C*H*CH_3_), 1.07 (d, *J* = 6.9 Hz, 3H, CH*CH*_3_), 0.79 (d, *J* = 6.9 Hz, 3H, CHC*H*_3_) ppm. ^13^C-NMR (67.5 MHz, CDCl_3_) δ: 197.2, 157.3 (q, ^2^*J_CF_* = 36.7 Hz), 134.4, 129.1 (2 × CH), 128.7 (2 × CH), 115.9 (q, ^1^*J_CF_* = 287.7 Hz), 58.6, 32.1, 19.9, 16.3 ppm. HRMS-ESI (*m*/*z*) [M + H]^+^ calcd for C_13_H_15_F_3_NO_2_ 274.1055, found 274.1057.

*(S)-2,2,2-Trifluoro-N-(4-methyl-1-oxo-1-phenylpentan-2-yl)acetamide* (**TFA**-**l**-**Leu**-**Ph**, **l**-**6c**): Colorless oil. [α]_D_ = +26 (*c* 2.0, CHCl_3_). IR (neat) *ν*: 3334, 3092, 2963, 1726, 1683 cm^−1^. ^1^H-NMR (270 MHz, CDCl_3_) δ: 7.98 (d, *J* = 7.3 Hz, 2H, Ar-H), 7.66 (t, *J* = 7.4 Hz, 1H, Ar-H), 7.54 (t, *J* = 7.6 Hz, 2H, Ar-H), 5.68 (td, *J* = 8.9, 2.6 Hz, 1H, C*H*NH), 1.79–1.49 (m, 3H, C*H*_2_C*H*), 1.10 (d, *J* = 5.9 Hz, 3H, CHC*H*_3_), 0.89 (d, *J* = 6.3 Hz, 3H, CHC*H*_3_) ppm. ^13^C-NMR (67.5 MHz, CDCl_3_) δ: 197.4, 156.9 (q, ^2^*J_CF_* = 37.4 Hz), 134.4, 133.6, 129.1 (2 × CH), 128.7 (2 × CH), 115.8 (q, ^1^*J_CF_* = 287.7 Hz), 52.9, 42.7, 25.1, 23.3, 21.7 ppm. HRMS-ESI (*m*/*z*) [M + H]^+^ calcd for C_14_H_17_F_3_NO_2_ 288.1211, found 288.1214.

*(R)-2,2,2-Trifluoro-N-(4-methyl-1-oxo-1-phenylpentan-2-yl)acetamide* (**TFA**-**d**-**Leu**-**Ph**, **d**-**6c**): Colorless oil. [α]_D_ = −26 (*c* 2.0, CHCl_3_). IR (neat) *ν*: 3335, 3094, 2931, 1731, 1685 cm^−1^. ^1^H-NMR (270 MHz, CDCl_3_) δ: 7.98 (d, *J* = 7.3 Hz, 2H, Ar-H), 7.66 (t, *J* = 7.3 Hz, 1H, Ar-H), 7.54 (t, *J* = 7.3 Hz, 2H, Ar-H), 5.68 (td, *J* = 9.1, 2.5 Hz, 1H, C*H*NH), 1.79–1.53 (m, 3H, C*H*_2_C*H*), 1.10 (d, *J* = 5.9 Hz, 3H, CHC*H*_3_), 0.89 (d, *J* = 5.9 Hz, 3H, CHC*H*_3_) ppm. ^13^C-NMR (67.5 MHz, CDCl_3_) δ: 197.4, 156.9 (q, ^2^*J_CF_* =37.4 Hz), 134.4, 133.6, 129.1 129.1 (2 × CH), 128.7 129.1 (2 × CH), 115.8 (q, ^1^*J_CF_* = 287.7 Hz), 52.9, 42.6, 25.1, 23.2, 21.7 ppm. HRMS-ESI (*m*/*z*) [M + H]^+^ calcd for C_14_H_17_F_3_NO_2_ 288.1211, found 288.1220.

*(S)-2,2,2-Trifluoro-N-(1-oxo-1-phenylpentan-2-yl)acetamide* (**TFA**-**l**-**Nva**-**Ph**, **l**-**7c**): Colorless oil. [α]_D_ = +46 (*c* 1.0, CHCl_3_). IR (neat) *ν*: 3341, 3074, 2979, 1733 cm^−1^. ^1^H-NMR (270 MHz, CDCl_3_) δ: 7.98 (d, *J* = 7.3 Hz, 2H, Ar-H), 7.67 (t, *J* = 7.3 Hz, 1H, Ar-H), 7.54 (t, *J* = 7.3 Hz, 2H, Ar-H), 7.44 (br s, 1H, N*H*), 5.62 (td, *J* = 7.4, 4.5 Hz, 1H, C*H*NH), 2.08–1.95 (m, 1H, CHC*H*_2_), 1.76–1.62 (m, 1H, CHC*H*_2_), 1.48–1.17 (m, 2H, C*H*_2_CH_3_) 0.90 (t, *J* = 7.3 Hz, 3H, CH_2_C*H*_3_) ppm. ^13^C-NMR (67.5 MHz, CDCl_3_) δ: 197.0, 156.8 (q, ^2^*J_CF_* = 37.1 Hz), 134.5, 133.6, 129.1 (2 × CH), 128.7 (2 × CH), 115.8 (q, ^1^*J_CF_* = 286.8 Hz), 54.4, 35.2, 18.0, 13.7 ppm. HRMS-ESI (*m*/*z*) [M + H]^+^ calcd for C_13_H_15_F_3_NO_2_ 274.1055, found 274.1064.

*(R)-2,2,2-Trifluoro-N-(1-oxo-1-phenylpentan-2-yl)acetamide* (**TFA**-**d**-**Nva**-**Ph**, **d**-**7c**): Colorless oil. [α]_D_ = −46 (*c* 1.0, CHCl_3_). IR (neat) *ν*: 3339, 3073, 2977, 1732 cm^−1^. ^1^H-NMR (270 MHz, CDCl_3_) δ: 7.99 (d, *J* = 7.4 Hz, 2H, Ar-H), 7.67 (t, *J* = 7.4 Hz, 1H, Ar-H), 7.54 (t, *J* = 7.4 Hz, 2H, Ar-H), 7.45 (br s, 1H, N*H*), 5.62 (td, *J* = 7.3, 4.4 Hz, 1H, C*H*NH), 2.08–1.95 (m, 1H, CHC*H*_2_), 1.76–1.62 (m, 1H, CHC*H*_2_), 1.48–1.13 (m, 2H, C*H*_2_CH_3_), 0.90 (t, *J* = 7.3 Hz, 3H, CH_2_C*H*_3_) ppm. ^13^C-NMR (67.5 MHz, CDCl_3_) δ: 197.0, 156.8 (q, ^2^*J_CF_* = 37.4 Hz), 134.5, 133.6, 129.1 (2 × CH), 128.7 (2 × CH), 115.8 (q, ^1^*J_CF_* = 288.3 Hz), 54.4, 35.2, 18.1, 13.7 ppm. HRMS-ESI (*m*/*z*) [M + H]^+^ calcd for C_13_H_15_F_3_NO_2_ 274.1055, found 274.1058.

*(S)-2,2,2-Trifluoro-N-(1-oxo-1-phenylhexan-2-yl)acetamide* (**TFA**-**l**-**Nle**-**Ph**, **l**-**8c**): Colorless oil. [α]_D_ = +60 (*c* 0.5, CHCl_3_). IR (neat) *ν*: 3326, 3068, 2960, 1728, 1687 cm^−1^. ^1^H-NMR (270 MHz, CDCl_3_) δ: 7.98 (d, *J* = 7.4 Hz, 2H, Ar-H), 7.66 (t, *J* = 7.4 Hz, 1H, Ar-H), 7.53 (t, *J* = 7.4 Hz, 2H, Ar-H), 7.46 (br s, 1H, N*H*), 5.61 (td, *J* = 7.3, 4.6 Hz, 1H, C*H*NH), 2.12–1.98 (m, 1H, CHC*H*_2_), 1.77–1.63 (m, 1H, CHC*H*_2_), 1.41–1.15 (m, 4H, 2 × CH_2_), 0.83 (t, *J* = 6.9 Hz, 3H, CH_2_C*H*_3_) ppm. ^13^C-NMR (67.5 MHz, CDCl_3_) δ: 197.0, 156.8 (q, ^2^*J_CF_* = 37.4 Hz), 134.4, 133.6, 129.0 (2 × CH), 128.6 (2 × CH), 115.8 (q, ^1^*J_CF_* = 287.7 Hz), 54.5, 32.7, 26.7, 22.2, 13.6 ppm. HRMS-ESI (*m*/*z*) [M + H]^+^ calcd for C_14_H_17_F_3_NO_2_ 288.1211, found 288.1224.

*(R)-2,2,2-Trifluoro-N-(1-oxo-1-phenylhexan-2-yl)acetamide* (**TFA**-**d**-**Nle**-**Ph**, **d**-**8c**): Colorless oil. [α]_D_ = −60 (*c* 0.5, CHCl_3_). IR (neat) *ν*: 3327, 3069, 2961, 1732, 1691 cm^−1^. ^1^H-NMR (270 MHz, CDCl_3_) δ: 7.99 (d, *J* = 7.3 Hz, 2H, Ar-H), 7.67 (t, *J* = 7.3 Hz, 1H, Ar-H), 7.54 (t, *J* = 7.3 Hz, 2H, Ar-H), 7.47 (br s, 1H, N*H*), 5.61 (td, *J* = 7.3, 4.6 Hz, 1H, C*H*NH), 2.12–1.98 (m, 1H, CHC*H*_2_), 1.78–1.63 (m, 1H, CHC*H*_2_), 1.39–1.14 (m, 4H, 2 × CH_2_), 0.83 (t, *J* = 6.9 Hz, 3H, CH_2_C*H*_3_) ppm. ^13^C-NMR (67.5 MHz, CDCl_3_) δ: 197.0, 156.8 (q, ^2^*J_CF_* = 37.4 Hz), 134.4, 133.6, 129.1 (2 × CH), 128.6 (2 × CH), 115.8 (q, ^1^*J_CF_* = 287.7 Hz), 54.5, 32.7, 26.7, 22.2, 13.6 ppm. HRMS-ESI (*m*/*z*) [M + H]^+^ calcd for C_14_H_17_F_3_NO_2_ 288.1211, found 288.1216.

## 4. Conclusions

Through the reaction of TFA-protected α-amino acid-OSu (l-/d-**1b**–l-/d-**2b**, **3b**, l-/d-**4b**–l-/d-**8b**) and arenes under conventional Friedel–Crafts conditions, it is more convenient to synthesize chiral TFA-protected α-amino aryl-ketone (l-/d-**1c**–l-/d-**2c**, **3c**, l-/d-**4c**–l-/d-**8b**) as its skeleton can be synthesized from optically active material. The TFA-protected α-amino acid-OSu (l-/d-**1b**–l-/d-**2b**, **3b**, l-/d-**4b**–l-/d-**8b**) showed high reactivity and is easier to handle during the reaction. The introduction of TFA-protected α-amino acid-OSu as a representative acyl donor can broaden the utilization of various materials for the Friedel–Crafts reaction. These materials contribute to new synthetic pathways and can be part of the comprehensive future study into their exploitation in bio-organic synthesis. 

## Figures and Tables

**Table 1:**
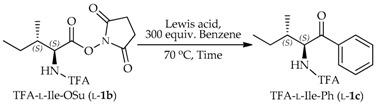
Friedel–Craft reaction of TFA-L-Ile-OSu (l-**1b**) and benzene with various Lewis acids.

Entry	Lewis acid (equiv.)	Time	TFA-L-Ile-Ph (L-1c, % Yield) ^a^
1	AlCl_3_ (1.5)	3 day	N.R. ^b^
2	AlCl_3_ (3)	9 h	N.R. ^c^
3	AlCl_3_ (6)	2 h	86
4	SnCl_2_ (6)	7 day ≤	N.R. ^d^
5	ZnCl_2_ (6)	7 day ≤	N.R. ^d^
6	FeCl_3_ (6)	2 day	N.R. ^d^
7	TiCl_4_ (6)	1 day	N.R. ^d^
8	GaCl_3_ (6)	2 h	4 ^c,e^
9	InCl_3_ (6)	2 h	N.R. ^b^

^a^ N.R. stands for no reaction. ^b^ Starting material of TFA-l-Ile-OSu (l-**1b**) was retained. ^c^ A complex mixture was recovered. ^d^ Hydrolysis is preferred rather than acylation. TFA-l-Ile (l-**1a**) was recovered. ^e^ Calculated from ^1^H-NMR.

## Supplementary Materials

Analytical data for TFA-α-amino acids, optimization for synthesis of TFA-α-amino acid-OSu, ^1^H- and ^13^C-NMR for all synthetic compounds are available online.
