# Supplementary material for: Synthesis of Chiral TFA-Protected α-Amino Aryl-Ketone Derivatives with Friedel–Crafts Acylation of α-Amino Acid N-Hydroxysuccinimide Ester
Source: Molecules. 2017 Oct 17;22(10):1748. doi: 10.3390/molecules22101748 (PMC6151496; doi:10.3390/molecules22101748)

Supplementary Materials

Synthesis of Chiral α-Amino Aryl-Ketone Derivatives with Friedel–Crafts Acylation of α-Amino Acid *N*-Hydroxysuccinimide Ester

Zetryana Puteri Tachrim,[1] Kazuhiro Oida,[1] Haruka Ikemoto,[1] Fumina Ohashi,[1] Natsumi Kurokawa,[1] Kento Hayashi,[1] Mami Shikanai,[1] Yasuko Sakihama,[1] Yasuyuki Hashidoko[1] and Makoto Hashimoto[1,*]

[1]*Division of Applied Science, Graduate School of Agriculture, Hokkaido University, Sapporo, Japan*

*Corresponding author. Email: hasimoto@abs.agr.hokudai.ac.jp

Contents

Scheme SM-1. Preparation of *N*-TFA α-Amino Acid. SI-2

Table SM-1 .Optimization of *N*-TFA α-Amino Acid *N*-Hydroxysuccinimide Ester **3b**–L-/D-**4b** or L-/D-**7b**–L-/D-**8b** Synthesis and optical rotation of previoys study SI-6

Scheme SM-2. NMR Spectrum SI-8

Scheme SM-1 Preparation of *N*-TFA α-Amino Acid.

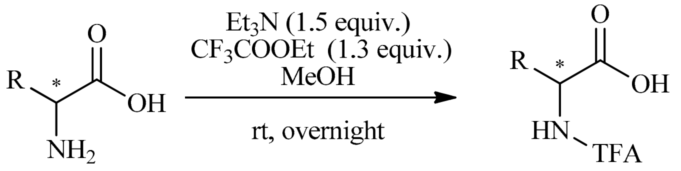

1a–8a

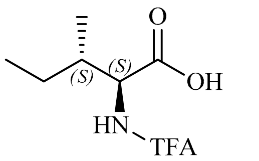

TFA-L-Ile (L-**1a**) 97%

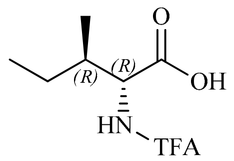

TFA-D-Ile (D-**1a**) 96%

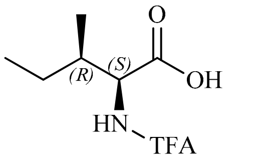

TFA-L-*allo*-Ile (L-**2a**) 99%

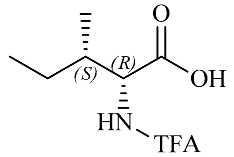

TFA-D-*allo*-Ile (D-**2a**) 89%

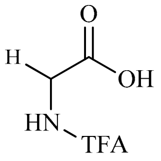

TFA-Gly (**3a**) 85%

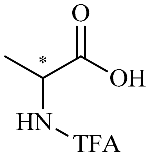

TFA-L-Ala (L-**4a**) 92%
TFA-D-Ala (D-**4a**) 95%

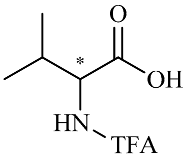

TFA-L-Val (L-**5a**) 92%
TFA-D-Val (D-**5a**) 90%

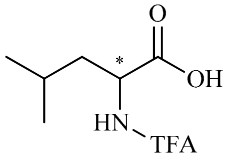

TFA-L-Leu (L-**6a**) 75%
TFA-D-Leu (D-**6a**) 85%

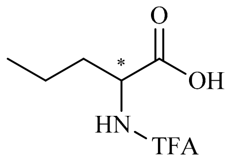

TFA-L-Nva (L-**7a**) 99%
TFA-D-Nva (D-**7a**) 99%

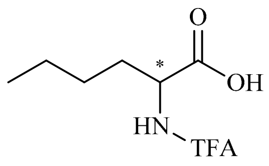

TFA-L-Nle (L-**8a**) quant.
TFA-D-Nle (D-**8a**) 95%

General procedure for the preparation of TFA-α-amino acid.
The TFA-α-amino acid was prepared with reported procedure [1,2] with slightly modification. Triethylamine (33 mmol, 1.5 equiv.) was added to a solution of α-amino acid (22 mmol) in MeOH (22 mL). After 5 min, ethyl trifluoroacetate (29 mmol, 1.3 equiv.) was added and the reaction was allowed to stir for 24 h. The solvent was removed by rotary evaporation and the residue that remained was dissolved in H_2O (35 mL) and acidified with concentrated HCl (4 mL). After stirring for 15 min, the mixture was extracted with ethyl acetate and the organic layers were combined and washed with brine, dried by $MgSO_4$, filtered, and concentrated by rotary evaporation. Further subjection into high vacuum for overnight, if needed to solidify the product (L-/D-**1a**–L-/D-**2a, 3a,** L-/D-**4a**–L-/D-**8a**).

(2*S*,3*S*)-3-Methyl-2-(2,2,2-trifluoroacetamido)pentanoic acid (TFA-L-Ile, L-1a) [1,2]. Colorless amorphous mass. $[\alpha]_D$ = +55 (*c* 1.0, $CHCl_3$). IR (neat) *ν*: 3294, 2968, 1740, 1694 cm^{-1}. ^{1}H-NMR (270 MHz, $CDCl_3$) δ: 10.58 (br s, 1H, COO*H*), 6.86 (d, *J* = 8.2 Hz, 1H, N*H*), 4.68 (dd, *J* = 8.4, 4.5 Hz, 1H, C*H*NH), 2.13–1.98 (m, 1H, C*H*CH_3), 1.60–1.44 (m, 1H, C$H_2$$CH_3$), 1.36–1.19 (m, 1H, C$H_2$$CH_3$), 1.01–0.94 (m, 6H, 2 x CH_3) ppm. ^{13}C-NMR (67.5 MHz, $CDCl_3$) δ: 175.4, 157.2 (q, $^2J_{CF}$ = 38.0 Hz), 115.6 (q, $^1J_{CF}$ = 287.7 Hz), 56.8, 37.6, 24.9, 15.2, 11.4 ppm. HRMS-ESI (*m/z*) $[M + H]^+$ calcd for $C_8H_{13}F_3NO_3$ 228.0848, found 228.0858.
(2*R*,3*R*)-3-Methyl-2-(2,2,2-trifluoroacetamido)pentanoic acid (TFA-D-Ile, D-1a). Colorless amorphous mass. $[\alpha]_D$ = −55 (*c* 1.0, $CHCl_3$). IR (neat) *ν*: 3293, 2973, 1740, 1699 cm^{-1}. ^{1}H-NMR (270 MHz, $CDCl_3$) δ: 9.47 (br s, 1H, COO*H*), 6.79 (d, *J* = 7.9 Hz, 1H, N*H*), 4.68 (dd, *J* = 8.4, 4.5 Hz, 1H, C*H*NH), 2.12–1.99 (m, 1H, C*H*CH_3), 1.61–1.44 (m, 1H, C$H_2$$CH_3$), 1.36–1.19 (m, 1H, C$H_2$$CH_3$), 1.01–0.95 (m, 6H, 2 x CH_3) ppm. ^{13}C-NMR (67.5 MHz, $CDCl_3$) δ: 175.3, 157.3 (q, $^2J_{CF}$ = 37.8 Hz), 115.6 (q, $^1J_{CF}$ = 287.5 Hz), 56.8, 37.6, 24.9, 15.1, 11.3 ppm. HRMS-ESI (*m/z*) $[M + H]^+$ calcd for $C_8H_{13}F_3NO_3$ 228.0848, found 228.0850.
(2*S*,3*R*)-3-Methyl-2-(2,2,2-trifluoroacetamido)pentanoic acid (TFA-L-*allo*-Ile, L-2a). Colorless amorphous mass. $[\alpha]_D$ = +24 (*c* 1.0, $CHCl_3$). IR (neat) *ν*: 3287, 2971, 1719 cm^{-1}. ^{1}H-NMR (270 MHz, $CDCl_3$) δ: 8.70 (br s, 1H, CHCOO*H*), 6.77 (d, *J* = 8.2 Hz, 1H, N*H*), 4.76 (dd, *J* = 8.6, 3.6 Hz, 1H, C*H*NH), 2.17–2.05 (m, 1H, C*H*CH_3), 1.53–1.38 (m, 1H, C$H_2$$CH_3$), 1.33–1.17 (m, 1H, C$H_2$$CH_3$), 1.01–0.90 (m, 6H, 2 x CH_3) ppm. ^{13}C-NMR (67.5 MHz, $CDCl_3$) δ: 175.6, 157.4 (q, $^2J_{CF}$ = 37.8 Hz), 115.7 (q, $^1J_{CF}$ = 287.5 Hz), 55.8, 37.6, 26.1, 14.3, 11.5 ppm. HRMS-ESI (*m/z*) $[M + H]^+$ calcd for $C_8H_{13}F_3NO_3$ 228.0848, found 228.0852.
(2*R*,3*S*)-3-Methyl-2-(2,2,2-trifluoroacetamido)pentanoic acid (TFA-D-*allo*-Ile, D-2a). Colorless amorphous mass. $[\alpha]_D$ = –24 (*c* 1.0, $CHCl_3$). IR (neat) *ν*: 3302, 2971, 1708 cm^{-1}. ^{1}H-NMR (270 MHz, $CDCl_3$) δ: 9.12 (br s, 1H, CHCOO*H*), 6.93 (d, *J* = 8.6 Hz, 1H, N*H*), 4.76 (dd, *J* = 8.9, 3.6 Hz, 1H, C*H*NH), 2.18–2.04 (m, 1H, C*H*CH_3), 1.53–1.37 (m, 1H, C$H_2$$CH_3$), 1.33–1.17 (m, 1H, C$H_2$$CH_3$), 1.01–0.94 (m, 6H, 2 x CH_3) ppm. ^{13}C-NMR (67.5 MHz, $CDCl_3$) δ: 175.7, 157.4 (q, $^2J_{CF}$ = 38.0 Hz), 115.7 (q, $^1J_{CF}$ = 287.7 Hz), 55.8, 37.5, 26.1, 14.3, 11.5 ppm. HRMS-ESI (*m/z*) $[M + H]^+$ calcd for $C_8H_{13}F_3NO_3$ 228.0848, found 228.0851.
2-(2,2,2-Trifluoroacetamido)acetic acid (TFA-Gly, 3a) [2,4,6]. Colorless amorphous mass. IR (neat) *ν*: 3299, 2992, 1682 cm^{-1}. ^{1}H-NMR (270 MHz, CD_3OD) δ: 4.01 (s, 2H, CH_2NH) ppm. ^{13}C NMR (67.5 MHz, CD_3OD) δ: 171.5, 159.4 (q, $^2J_{CF}$ = 37.4 Hz), 117.4 (q, $^1J_{CF}$ = 286.2 Hz), 41.7 ppm. HRMS-ESI (*m/z*) $[M + H]^+$ calcd for $C_4H_5F_3NO_3$ 172.0222, found 172.0241.
(*S*)-2-(2,2,2-Trifluoroacetamido)propanoic acid (TFA-L-Ala, L-4a) [2,3,7]. Colorless amorphous mass. $[\alpha]_D$ = +38 (*c* 1.0, $CHCl_3$). IR (neat) *ν*: 3330, 2952, 1752 cm^{-1}. ^{1}H-NMR (270 MHz, $CDCl_3$) δ: 9.23 (br s, 1H, COO*H*), 7.00 (br s, 1H, N*H*), 4.72–4.62 (m, 1H, C*H*CH_3), 1.58

(d, J = 7.3 Hz, 3H, CHCH_3) ppm. ^{13}C NMR (67.5 MHz, $CDCl_3$) δ: 176.3, 156.9 (q, $^2J_{CF}$ = 38.2 Hz), 115.5 (q, $^1J_{CF}$ = 287.9 Hz), 48.5, 17.6 ppm. HRMS-ESI (m/z) [M + H]$^+$ calcd for $C_5H_7F_3NO_3$ 186.0378, found 186.0389.

(*R*)-2-(2,2,2-Trifluoroacetamido)propanoic acid (TFA-D-Ala, D-4a) [7]. Colorless amorphous mass. $[\alpha]_D$ = –38 (c 1.0, $CHCl_3$). IR (neat) ν: 3295, 2949, 1756 cm^{-1}. ^{1}H-NMR (270 MHz, $CDCl_3$) δ: 8.96 (br s, 1H, COOH), 6.99 (br s, 1H, NH), 4.73–4.62 (m, 1H, CHCH$_3$), 1.58 (d, J = 7.3 Hz, 3H, CHCH_3) ppm. ^{13}C NMR (67.5 MHz, $CDCl_3$) δ: 176.0, 157.1 (q, $^2J_{CF}$ = 38.2 Hz), 115.5 (q, $^1J_{CF}$ = 287.2 Hz), 48.5, 17.2 ppm. HRMS-ESI (m/z) [M + H]$^+$ calcd for $C_5H_7F_3NO_3$ 186.0378, found 186.0365.

(*S*)-3-Methyl-2-(2,2,2-trifluoroacetamido)butanoic acid (TFA-L-Val, L-5a) [2–4]. Colorless amorphous mass. $[\alpha]_D$ = +53 (c 1.0, $CHCl_3$). IR (neat) ν: 3286, 2969, 1739 cm^{-1}. ^{1}H-NMR (270 MHz, CD_3Cl_3) δ: 10.35 (br s, 1H, CHCOOH), 6.81 (d, J = 7.9 Hz, 1H, NH), 4.65 (dd, J = 8.6, 4.6 Hz, 1H, CHNH), 2.41–2.29 (m, 1H, CHCH$_3$), 1.05–0.99 (m, 5H, 2 x CH_3) ppm. ^{13}C-NMR (67.5 MHz, $CDCl_3$) δ: 175.5, 157.3 (q, $^2J_{CF}$ = 36.3 Hz), 115.7 (q, $^1J_{CF}$ = 287.2 Hz), 57.4, 31.1, 18.7, 17.4 ppm. HRMS-ESI (m/z) [M + Na]$^+$ calcd for $C_7H_{10}F_3NO_3Na$ 236.0510, found 236.0520.

(*R*)-3-Methyl-2-(2,2,2-trifluoroacetamido)butanoic acid (TFA-D-Val, D-5a) [4]. Colorless amorphous mass. $[\alpha]_D$ = –53 (c 1.0, $CHCl_3$). IR (neat) ν: 3295, 2970, 1753 cm^{-1}. ^{1}H-NMR (270 MHz, CD_3Cl_3) δ: 10.99 (br s, 1H, CHCOOH), 6.89 (d, J = 8.6 Hz, 1H, NH), 4.64 (dd, J = 8.6, 4.6 Hz, 1H, CHNH), 2.41–2.26 (m, 1H, CHCH$_3$), 1.05–0.99 (m, 6H, 2 x CH_3) ppm. ^{13}C-NMR (67.5 MHz, $CDCl_3$) δ: 175.5, 157.3 (q, $^2J_{CF}$ = 37.4 Hz), 115.6 (q, $^1J_{CF}$ = 287.7 Hz), 57.4, 31.1, 18.7, 17.4 ppm. HRMS-ESI (m/z) [M + Na]$^+$ calcd for $C_7H_{10}F_3NO_3Na$ 236.0510, found 236.0518

(*S*)-4-methyl-2-(2,2,2-trifluoroacetamido)pentanoic acid (TFA-L-Leu, L-6a) [1,4,5]. Colorless amorphous mass. $[\alpha]_D$ = +24 (c 1.0, $CHCl_3$). IR (neat) ν: 3294, 2963, 1731 cm^{-1}. ^{1}H-NMR (270 MHz, CD_3Cl_3) δ: 8.90 (br s, 1H, CHCOOH), 6.78 (br s, 1H, NH), 4.74–4.65 (m, 1H, CHNH), 1.88–1.64 (m, 3H, CH_2CH), 1.00 (s, 3H, CH_3), 0.98 (s, 3H, CH_3) ppm. ^{13}C-NMR (67.5 MHz, $CDCl_3$) δ: 176.4, 157.2 (q, $^2J_{CF}$ = 38.0 Hz), 115.6 (q, $^1J_{CF}$ = 287.2 Hz), 51.1, 40.8, 24.8, 22.6, 21.6 ppm. HRMS-ESI (m/z) [M + H]$^+$ calcd for $C_8H_{13}F_3NO_3$ 228.0848, found 228.0859.

(*R*)-4-Methyl-2-(2,2,2-trifluoroacetamido)pentanoic acid (TFA-D-Leu, D-6a) [4]. Colorless amorphous mass. $[\alpha]_D$ = –24 (c 1.0, $CHCl_3$). IR (neat) ν: 3300, 2965, 1733 cm^{-1}. ^{1}H-NMR (270 MHz, CD_3Cl_3) δ: 8.54 (br s, 1H, CHCOOH), 6.73 (d, J = 7.6 Hz, 1H, NH), 4.74–4.65 (m, 1H, CHNH), 1.88–1.61 (m, 3H, CH_2CH), 1.00 (s, 3H, CH_3), 0.98 (s, 3H, CH_3) ppm. ^{13}C-NMR (67.5 MHz, $CDCl_3$) δ: 175.9, 157.8 (q, $^2J_{CF}$ = 38.0 Hz), 115.6 (q, $^1J_{CF}$ = 287.0 Hz,), 51. 2, 40. 2, 24.7, 22.4, 21.2 ppm. HRMS-ESI (m/z) [M + H]$^+$ calcd for $C_8H_{13}F_3NO_3$ 228.0848, found 228.0865.

(*S*)-2-(2,2,2-Trifluoroacetamido)pentanoic acid (TFA-L-Nva, L-7a) [4]. Colorless amorphous mass. $[\alpha]_D$ = +58 (c 1.0, $CHCl_3$). IR (neat) ν: 3292 cm^{-1}, 2967, 1732, 1696 cm^{-1}. ^{1}H-NMR (270 MHz, $CDCl_3$) δ: 6.76 (d, J = 6.9 Hz, 1H, NH), 4.69 (td, J = 7.3, 5.4 Hz, 1H, CHNH), 2.06–1.92 (m, 1H, CHCH_2), 1.88–1.74 (m, 1H, CHCH_2), 1.50–1.35 (m, 2H, CH_2CH$_3$), 0.98 (t, J = 7.3 Hz, 3H, CH$_2$CH_3) ppm. ^{13}C-NMR (67.5 MHz, $CDCl_3$) δ: 176.0, 157.3 (q, $^2J_{CF}$ = 37.8 Hz), 115.6 (q, $^1J_{CF}$ = 287.3 Hz), 52.4, 33.5, 18.4, 13.3 ppm. HRMS-ESI (m/z) [M + H]$^+$ calcd for $C_7H_{11}F_3NO_3$ 214.0691, found 214.0693.

(*R*)-2-(2,2,2-Trifluoroacetamido)pentanoic acid (TFA-D-Nva, D-7a) [4]. Colorless amorphous mass. $[\alpha]_D$ = –58 (c 1.0, $CHCl_3$). IR (neat) ν: 3319, 2969, 1745, 1695 cm^{-1}. ^{1}H-NMR (270 MHz, $CDCl_3$) δ: 6.75 (d, J = 6.6 Hz, 1H, NH), 4.69 (td, J = 7.5, 5.2 Hz, 1H, CHNH), 2.06–1.92 (m, 1H, CHCH_2), 1.88–1.74 (m, 1H, CHCH_2), 1.57–1.32 (m, 2H, CH_2CH$_3$), 0.98 (t, J = 7.3 Hz, 3H, CH$_2$CH_3) ppm. ^{13}C-NMR (67.5 MHz, $CDCl_3$) δ: 176.2, 157.2 (q, $^2J_{CF}$ = 38.0 Hz), 115.6 (q, $^1J_{CF}$

= 287.3 Hz), 52.4, 33.6, 18.4, 13.4 ppm. HRMS-ESI (*m*/*z*) $[M + H]^+$ calcd for $C_7H_{11}F_3NO_3$ 214.0691, found 214.0696.

(*S*)-2-(2,2,2-Trifluoroacetamido)hexanoic acid (TFA-L-Nle, L-8a) [4]**.** Colorless amorphous mass. $[\alpha]_D$ = +67 (*c* 1.0, $CHCl_3$). IR (neat) *ν*: 3314, 2936, 1728 cm^{-1}. ^1H-NMR (270 MHz, $CDCl_3$) δ: 6.73 (br s, 1H, N*H*), 4.67 (td, *J* = 7.4, 5.4 Hz, 1H, C*H*NH), 2.08–1.94 (m, 1H, CHCH_2), 1.89–1.75 (m, 1H, CHCH_2), 1.42–1.26 (m, 4H, 2 x CH_2), 0.92 (t, *J* = 6.9 Hz, 3H, CH_2CH_3) ppm. ^{13}C-NMR (67.5 MHz, $CDCl_3$) δ: 176.1, 157.2 (q, $^2J_{CF}$ = 38.0 Hz), 115.6 (q, $^1J_{CF}$ = 287.5 Hz), 52.6, 31.3, 27.0, 22.1, 13.6 ppm. HRMS-ESI (*m*/*z*) $[M + H]^+$ calcd for $C_8H_{13}F_3NO_3$ 228.0848, found 228.0850.

(*R*)-2-(2,2,2-Trifluoroacetamido)hexanoic acid (TFA-D-Nle, D-8a) [4]**.** Colorless amorphous mass. $[\alpha]_D$ = –67 (*c* 1.0, $CHCl_3$). IR (neat) *ν*: 3302, 2936, 1740 cm^{-1}. ^1H-NMR (270 MHz, $CDCl_3$) δ: 6.74 (br s, 1H, N*H*), 4.69 (td, *J* = 7.4, 5.4 Hz, 1H, C*H*NH), 2.06–1.94 (m, 1H, CHCH_2), 1.89–1.75 (m, 1H, CHCH_2), 1.41–1.31 (m, 4H, 2 x CH_2), 0.92 (t, *J* = 6.9 Hz, 3H, CH_2CH_3) ppm. ^{13}C-NMR (67.5 MHz, $CDCl_3$) δ: 176.2, 157.1 (q, $^2J_{CF}$ = 38.0 Hz), 115.6 (q, $^1J_{CF}$ = 287.7 Hz), 52.5, 31.4, 27.0, 22.1, 13.6 ppm. HRMS-ESI (*m*/*z*) $[M + H]^+$ calcd for $C_8H_{13}F_3NO_3$ 228.0848, found 228.0843.

[1] Curphey T J. Trifluoracetylation of amino acids and peptides by ethyl trifluoroacetate. J. Org. Chem. 1979, 44, 2805–2807.

[2] Deblander J, Van Aeken S, Jacobs J, De Kimpe N, Tehrani K A. A new synthesis of benzo[*f*]isoindole-4,9-diones by radical alkylation and bromomethylation of 1,4-naphthoquinones. Eur. J. Org. Chem. 2009, 4882–4892.

[3] Jass, P. A.; Rosso, V. W.; Racha, S.; Soundararajan, N.; Venit, J. J.; Rusowicz, A.; Swaminathan, S.; Livshitz, J.; Delaney, E. J. Use of N-trifluoroacetyl-protected amino acid chlorides in peptide coupling reactions with virtually complete preservation of stereochemistry. Tetrahedron 2003, 59, 9019–9029.

[4] Weygand, F.; Ropsch, A. N-Trifluoroacetylamino acids. XIV. N-Trifluoroacetylations of amino acids and peptides with phenyl trifluoroacetate. Chem. Ber. 1959, 92, 2095–2099.

[5] Chambers, J. J.; Kurrasch-Orbaugh, D. M.; Parker, M. A.; Nichols, D. E. Enantiospecific synthesis and pharmacological evaluation of a series of super-potent, conformationally restricted 5-HT2A/2C receptor agonists. J. Med. Chem. 2001, 44, 1003–1010.

[6] Fones, W. S.; Lee, M. Hydrolysis of the N-trifluoroacetyl derivative of several D- and L-amino acids by acylase I. J. Biol. Chem. 1954, 210, 227–238.

[7] Reay, A. J.; Williams, T. J.; Fairlamb, I. J. S. Unified mild reaction conditions for C2-selective Pd-catalysed tryptophan arylation, including tryptophan-containing peptides. Org. Biomol. Chem. 2015, 13, 8298–8309.

[8] Jagt, R. B. C.; Gómez-Biagi, R. F.; Nitz, M. Pattern-based recognition of heparin contaminants by an array of self-assembling fluorescent receptors. Angew. Chemie - Int. Ed. 2009, 48, 1995–1997.

[9] Fones, W. S. Some new N-acyl derivatives of alanine and phenylalanine. J. Org. Chem. 1952, 17, 1661–1665.

Table SM-1 Optimization of *N*-TFA α-Amino Acid *N*-Hydroxysuccinimide Ester 3b–L-/D-4b or L-/D-7b–L-/D-8b Synthesis[a]

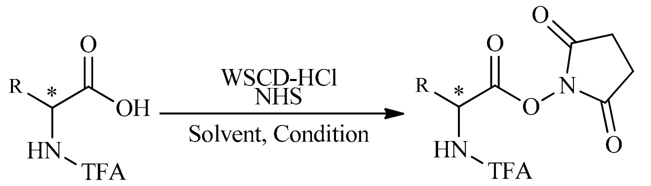

3a–L-/D-**4a** or L-/D-**7a**–L-/D-**8a** → **3b**–L-/D-**4b** or L-/D-**7b**–L-/D-**8b**

Entry	Material	NHS (equiv.)	WSCD-HCl (equiv.)	Solvent	Condition		Product	Isolated Yield (%)
					Temperature	Time		
1	**3a**	1.1	1.0	CH_2Cl_2	rt	3 h	**3b**	17[c]
2	**3a**	1.1	1.0[b]	DMF	rt	1 h	**3b**	quant.[d]
3	L-**4a**	1.3	1.3[b]	DMF	rt	3 h	L-**4b**	60[e]
4	D-**4a**	1.3	1.3[b]	DMF	rt	3 h	D-**4b**	53[e]
5	L-**4a**	1.1	1.3[b]	Acetone	rt	3 h	L-**4b**	53[e]
6	D-**4a**	1.1	1.3[b]	Acetone	rt	3 h	D-**4b**	52[e]
7	L-**4a**	1.1	1.3[b]	CH_2Cl_2	rt	3 h	L-**4b**	51[e]
8	D-**4a**	1.1	1.3[b]	CH_2Cl_2	rt	3 h	D-**4b**	56[e]
9	L-**4a**	1.1	1.0	CH_2Cl_2	rt	3 h	L-**4b**	52
10	D-**4a**	1.1	1.0	CH_2Cl_2	rt	3 h	D-**4b**	42
11	L-**4a**	1.1	1.0[b]	CH_2Cl_2	rt	3 h	L-**4b**	75[f]
12	D-**4a**	1.1	1.0[b]	CH_2Cl_2	rt	3 h	D-**4b**	71[f]
13	L-**7a**	1.1	1.0	CH_2Cl_2	0 °C	3.5 h	L-**7b**	68
14	D-**7a**	1.1	1.0	CH_2Cl_2	0 °C	3.5 h	D-**7b**	64
15	L-**7a**	1.1	1.1	CH_2Cl_2	0 °C	3.5 h	L-**7b**	89
16	D-**7a**	1.1	1.1	CH_2Cl_2	0 °C	3.5 h	D-**7b**	88
17	L-**8a**	1.1	1.0	CH_2Cl_2	0 °C	3.5 h	L-**8b**	64[g]
18	D-**8a**	1.1	1.0	CH_2Cl_2	0 °C	3.5 h	D-**8b**	71[g]
19	L-**8a**	1.1	1.1	CH_2Cl_2	0 °C	3.5 h	L-**8b**	78
20	D-**8a**	1.1	1.1	CH_2Cl_2	0 °C	3.5 h	D-**8b**	75

[a]General procedure: material (1 mmol). Reaction mixture is pre-cooled before addition of the suspense of WSCD-HCl (1 mmol, 1.0 equiv.) in dichloromethane (10 mL). Otherwise mentioned; purification is conducted by washing the reaction mixture with H_2O and sat. NaCl.
[b]WSCD-HCl is directly added into reaction.
[c]Solvent was removed under reduced pressure. Then, residue was dissolve in ethyl acetate and washed by H_2O, sat. $NaHCO_3$, and sat. NaCl.
[d]Solvent was removed under reduced pressure. Then, residue was dissolve in ethyl acetate and washed by 1M HCl
[e]Solvent was removed under reduced pressure. Then, residue was dissolve in ethyl acetate and washed by H_2O, 1M HCl, sat. $NaHCO_3$, and sat. NaCl.
[f]Reaction mixture is directly washed by sat. NaCl.
[g]Contaminated with unidentified compound, observed by appearance of other α-proton ^{1}H-NMR (ratio 1.00 : 0.06)

Optical rotation of previous study

Based on previous study [1] (optical rotations were measured at 546 nm at 20 °C), the reported optical rotation of TFA-L-Ile-OSu (L-**1b**) $[\alpha]_D = -63.6$ (c 1, CH_3OH); TFA-L-Val-OSu (L-**5b**) $[\alpha]_D = -73.3$ (c 1, CH_3OH); TFA-L-Leu-OSu (L-**6b**) $[\alpha]_D = -50.8$ (c 1, CH_3OH), respectively.

---

[1] Weygand, F.; Frauendorfer, E. *N*-(Trifluoroacetyl)amino acids. XXI. Reductive elimination of the *N*-trifluoroacetyl and *N*-trichloroacetyl groups by sodium borohydride and applications in peptide chemistry. Chem. Ber. 1970, *103*, 2437–2449.

Scheme SM-2 NMR Spectrum

(2*S*,3*S*)-3-Methyl-2-(2,2,2-trifluoroacetamido)pentanoic acid (TFA-L-Ile, L-1a)

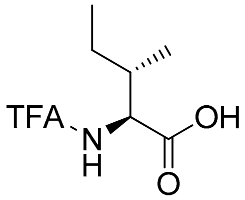

^{1}H NMR (270 MHz, $CDCl_3$)

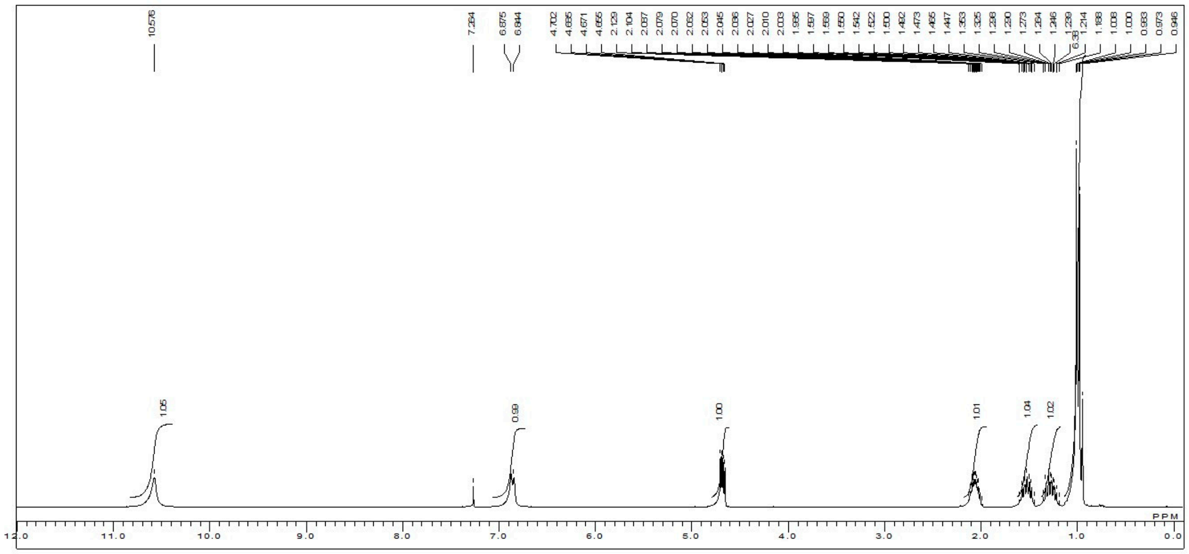

^{13}C NMR (67.5 MHz, $CDCl_3$)

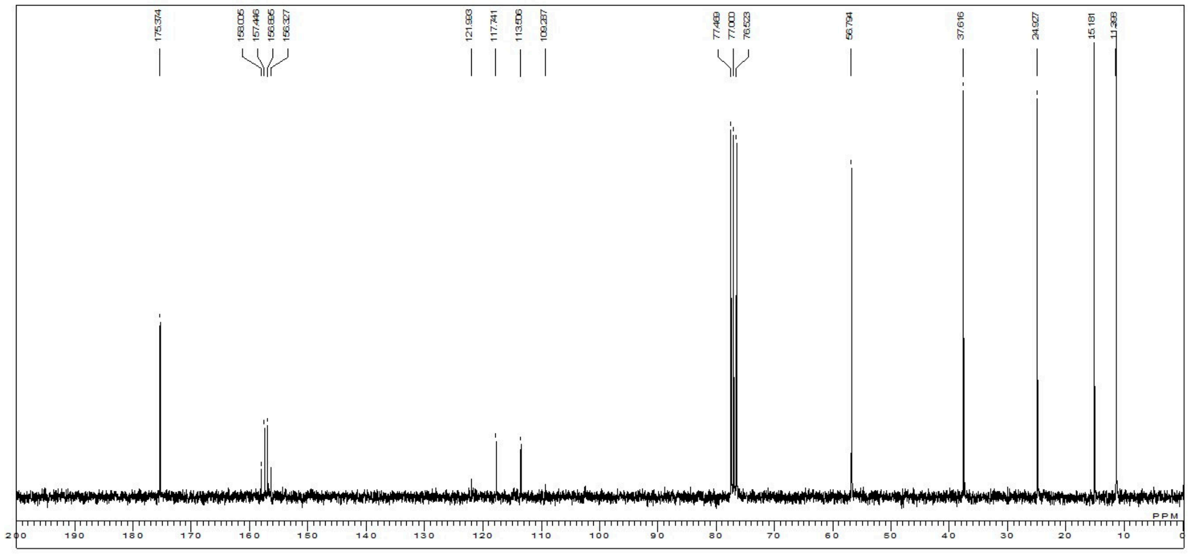

(2*R*,3*R*)-3-Methyl-2-(2,2,2-trifluoroacetamido)pentanoic acid (TFA-D-Ile, D-1a)

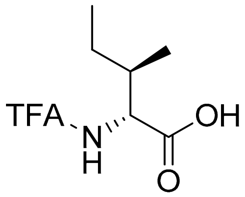

^{1}H NMR (270 MHz, $CDCl_3$)

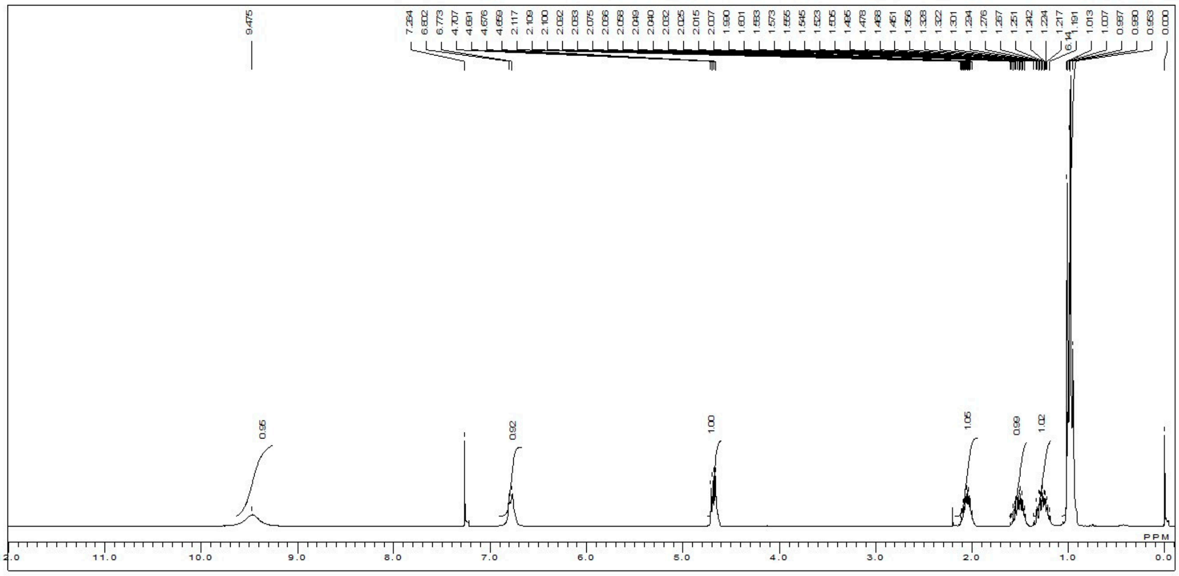

^{13}C NMR (67.5 MHz, $CDCl_3$)

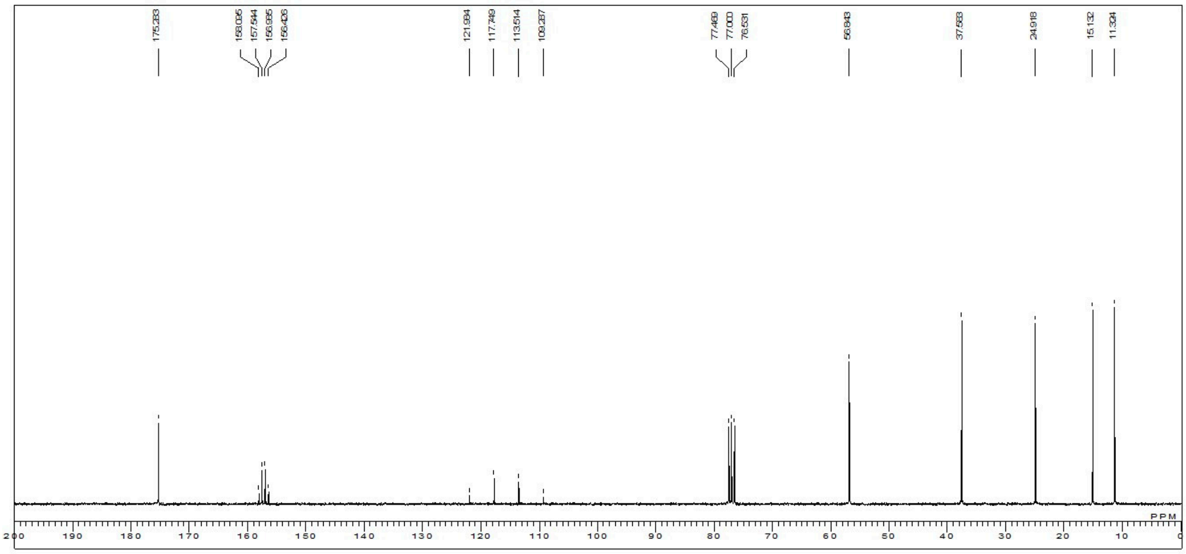

(2*S*,3*S*)-2,5-Dioxopyrrolidin-1-yl 3-methyl-2-(2,2,2-trifluoroacetamido)pentanoate (TFA-L-Ile-OSu, L-1b)

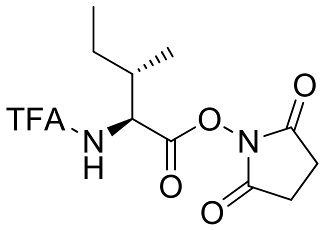

^{1}H NMR (270 MHz, CDCl$_3$)

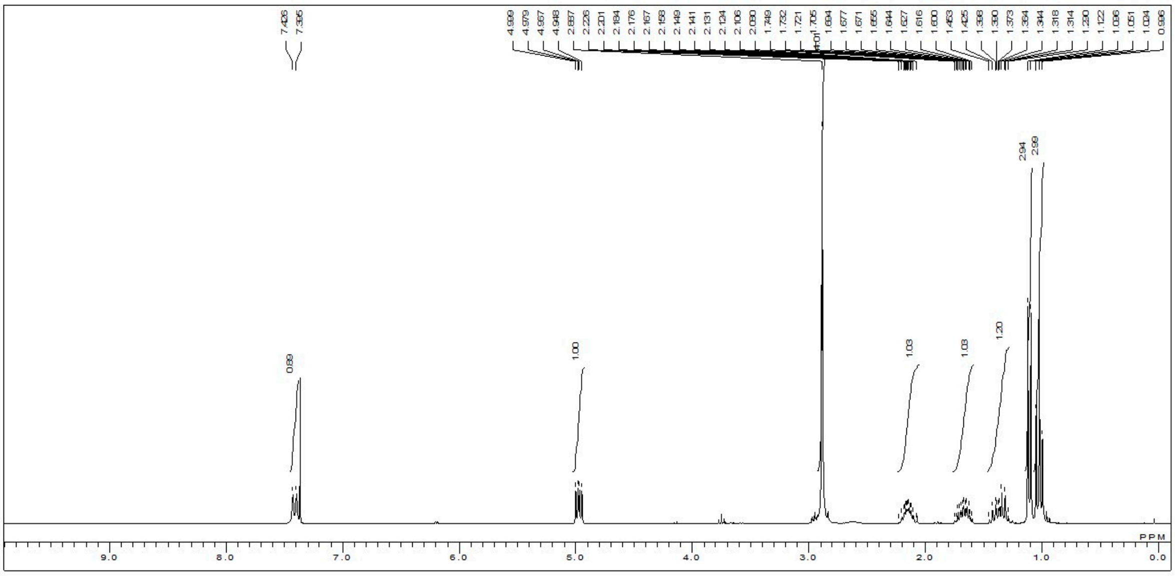

^{13}C NMR (67.5 MHz, CDCl$_3$)

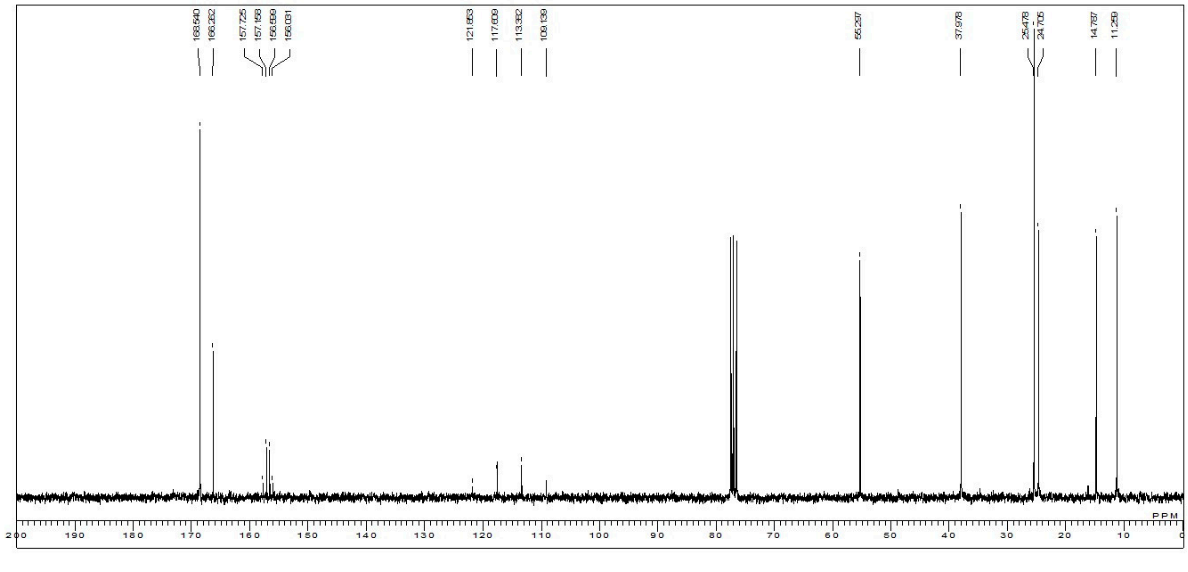

(2*R*,3*R*)-2,5-Dioxopyrrolidin-1-yl 3-methyl-2-(2,2,2-trifluoroacetamido)pentanoate (TFA-D-Ile-OSu, D-1b)

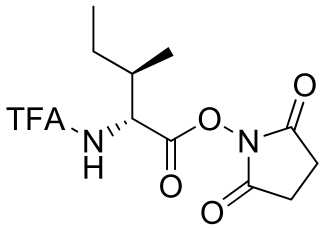

^{1}H NMR (270 MHz, $CDCl_3$)

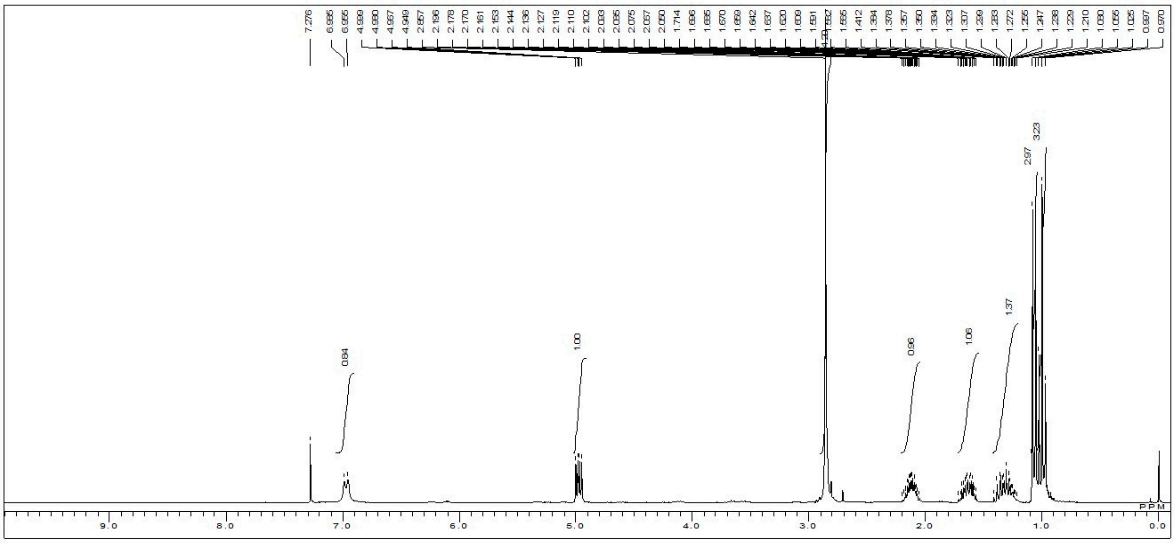

^{13}C NMR (67.5 MHz, $CDCl_3$)

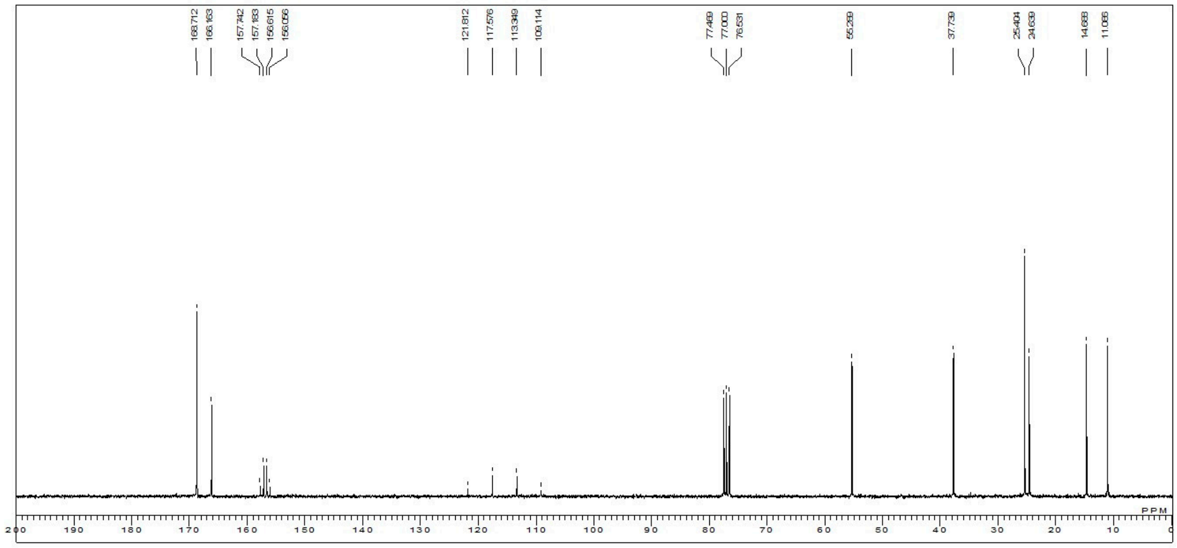

2,2,2-Trifluoro-*N*-((2*S*,3*S*)-3-methyl-1-oxo-1-phenylpentan-2-yl)acetamide (TFA-L-Ile-Ph, L-1c)

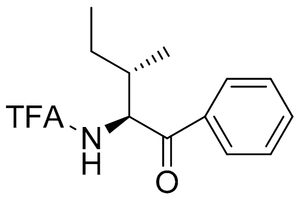

^{1}H NMR (270 MHz, $CDCl_3$)

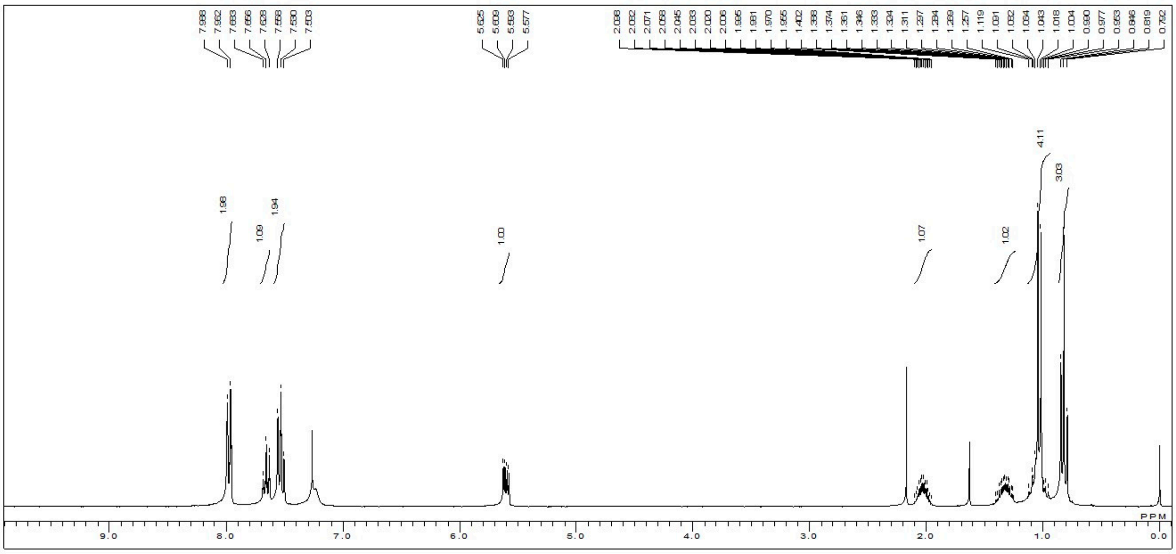

^{13}C NMR (67.5 MHz, $CDCl_3$)

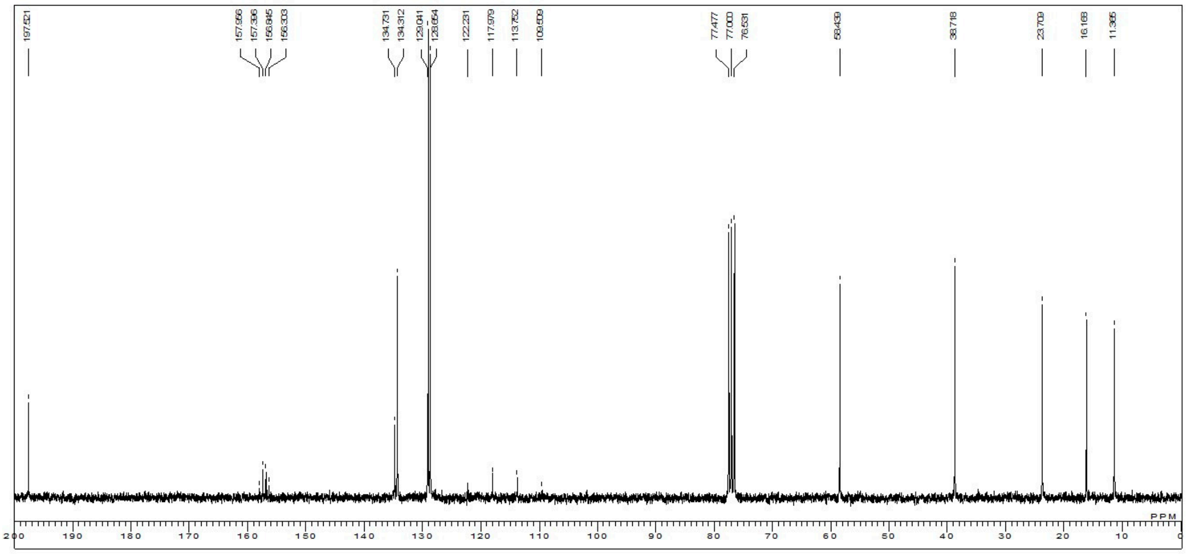

2,2,2-Trifluoro-*N*-((2R,3R)-3-methyl-1-oxo-1-phenylpentan-2-yl)acetamide (TFA-D-Ile-Ph, D-1c)

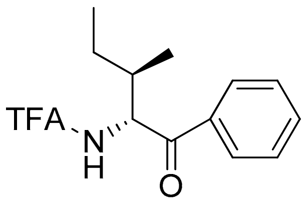

^{1}H-NMR (270 MHz, $CDCl_3$)

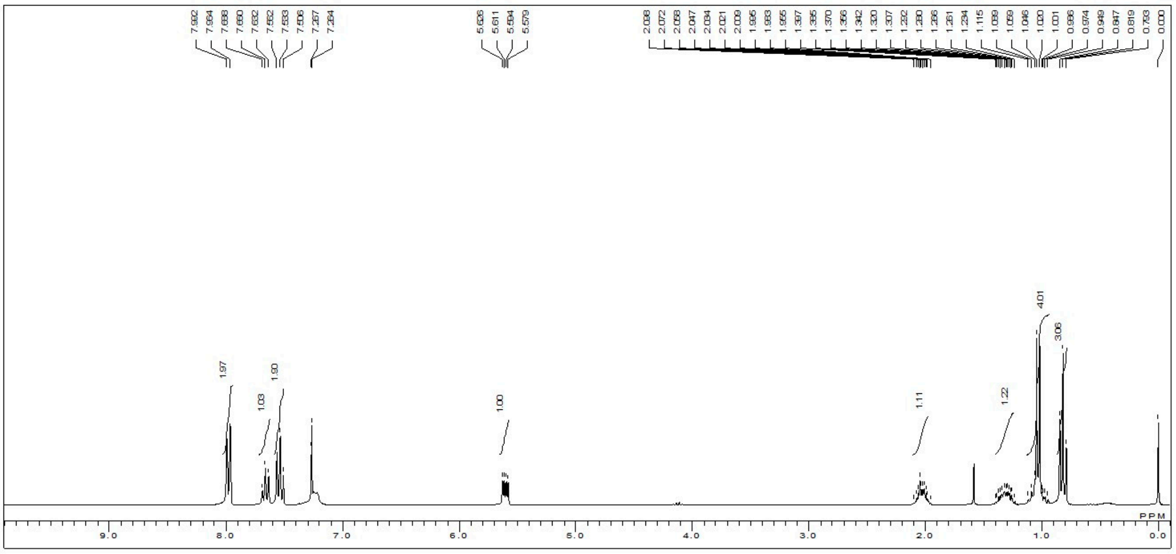

^{13}C-NMR (67.5 MHz, $CDCl_3$)

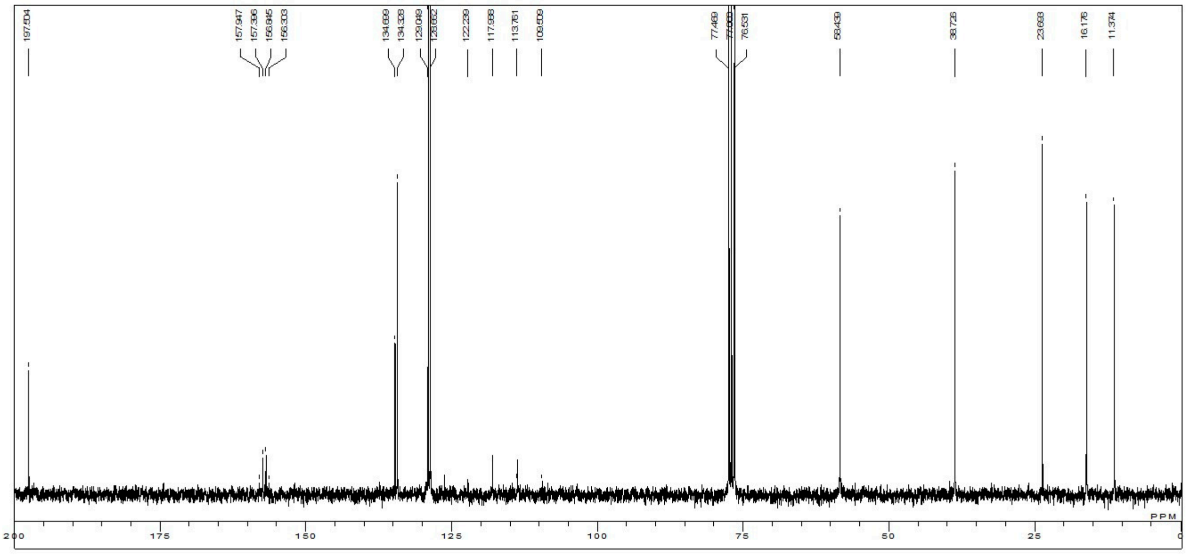

2,2,2-Trifluoro-*N*-((2*S*,3*S*)-3-methyl-1-oxo-1-(p-tolyl)pentan-2-yl)acetamide (TFA-L-Ile-Ph(4-Me), L-1d)

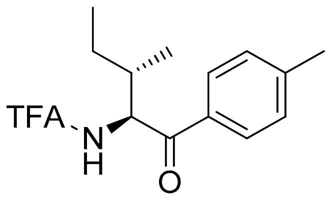

^{1}H NMR (270 MHz, $CDCl_3$)

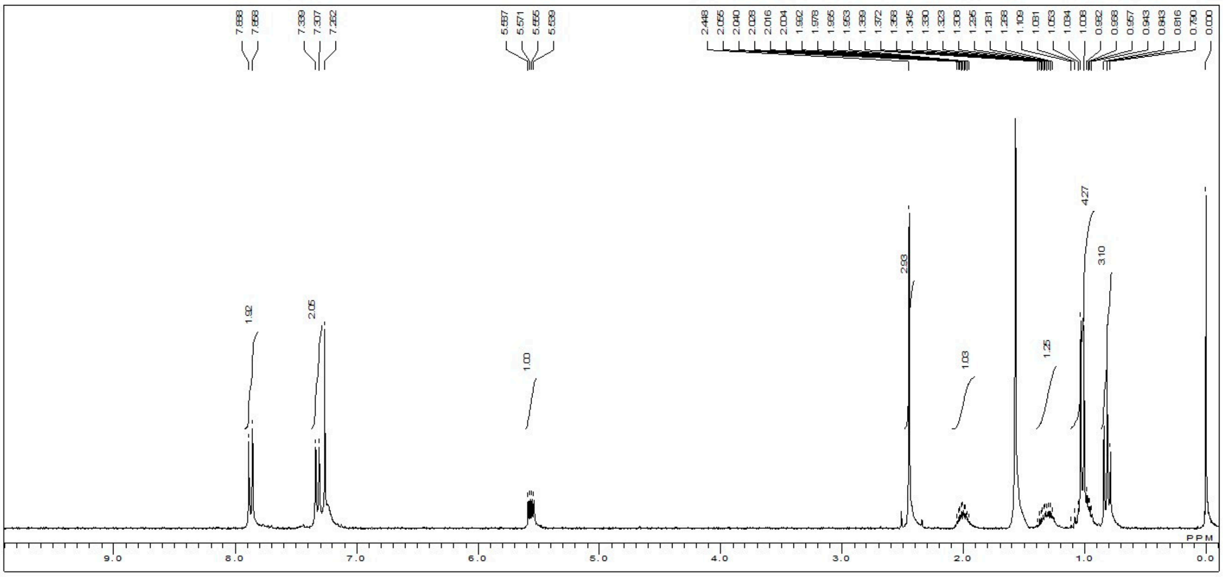

^{13}C NMR (67.5 MHz, $CDCl_3$)

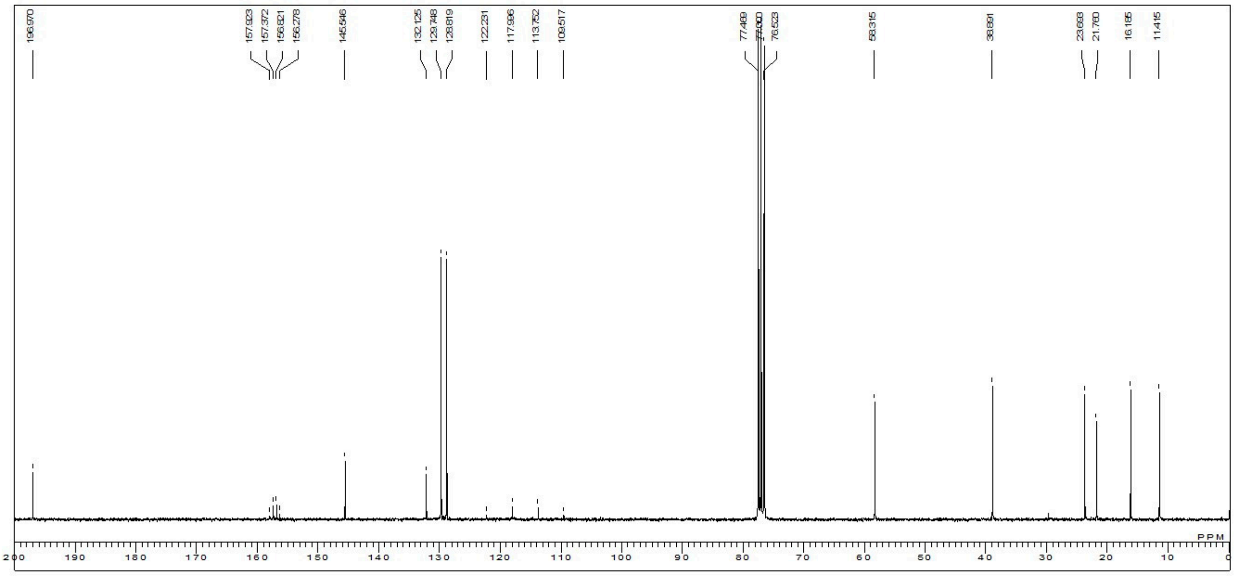

2,2,2-Trifluoro-*N*-((2S,3S)-1-(4-methoxyphenyl)-3-methyl-1-oxopentan-2-yl)acetamide (TFA-L-Ile- Ph(4-OMe), L-1e)

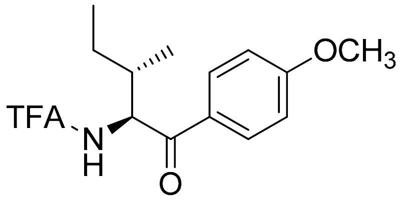

^{1}H NMR (270 MHz, $CDCl_3$)

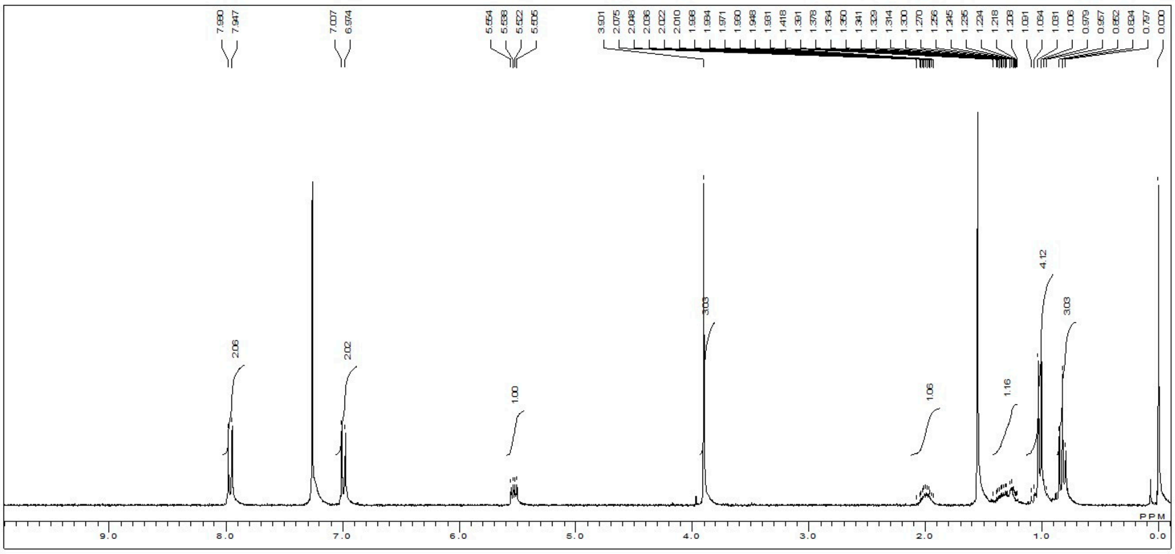

^{13}C NMR (67.5 MHz, $CDCl_3$)

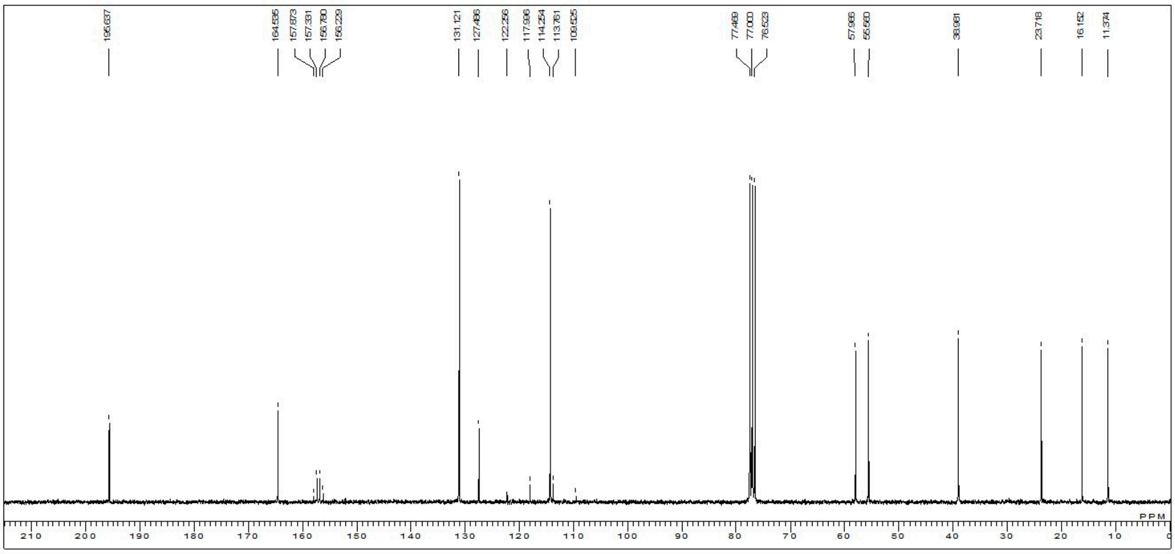

***N*-((2*S*,3*S*)-1-(3,4-Dimethylphenyl)-3-methyl-1-oxopentan-2-yl)-2,2,2-trifluoroacetamide (TFA-L-Ile-Ph(3,4-Me), L-1f)**

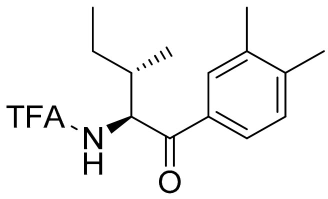

^{1}H NMR (270 MHz, $CDCl_3$)

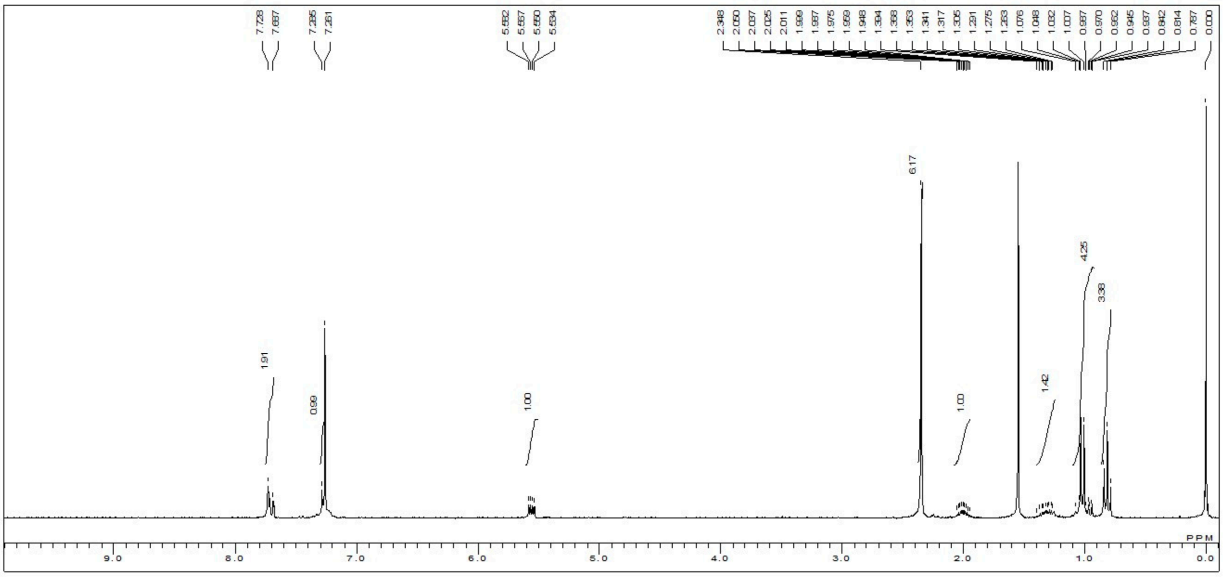

^{13}C NMR (67.5 MHz, $CDCl_3$)

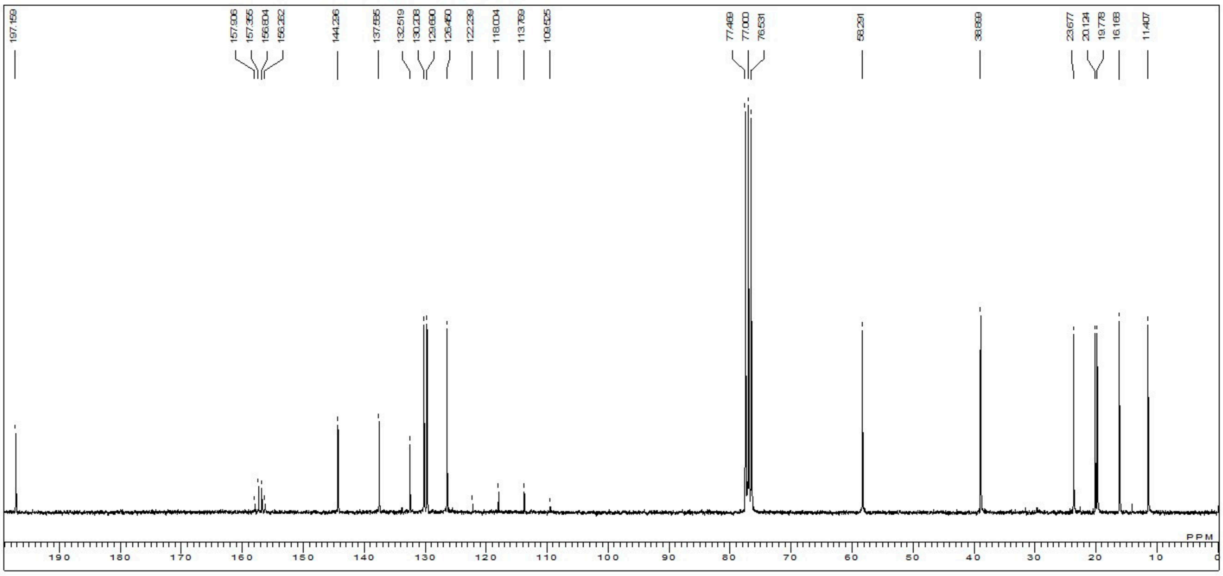

N-((2S,3S)-1-(2,4-Dimethylphenyl)-3-methyl-1-oxopentan-2-yl)-2,2,2-trifluoroacetamide (TFA-L-Ile- Ph(2,4-Me), L-1g)

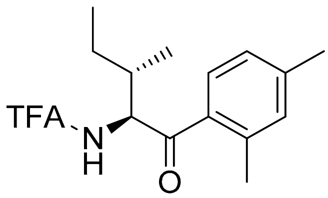

^{1}H NMR (270 MHz, $CDCl_3$)

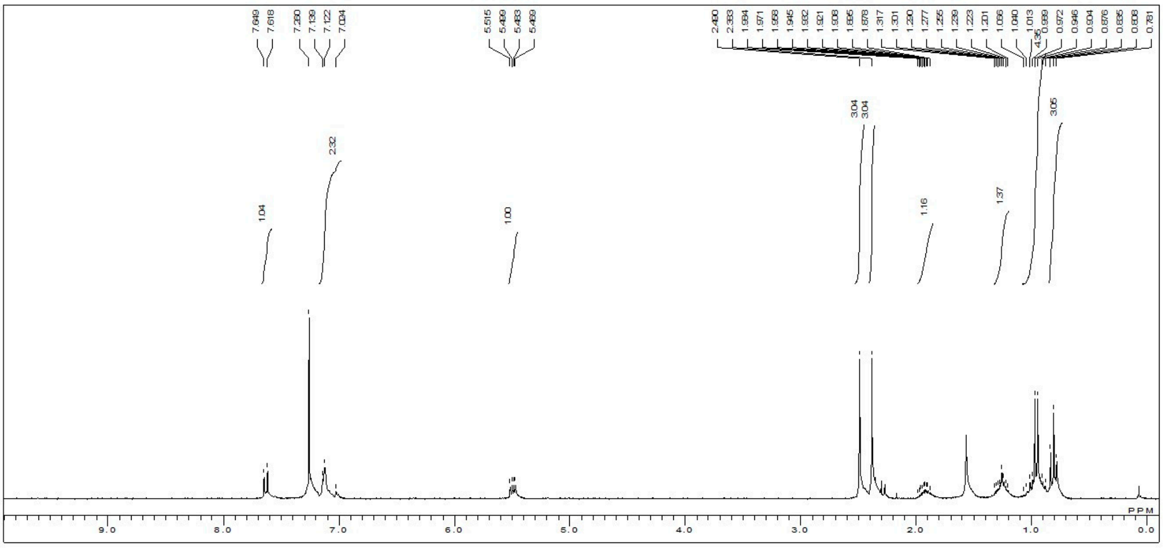

^{13}C NMR (67.5 MHz, $CDCl_3$)

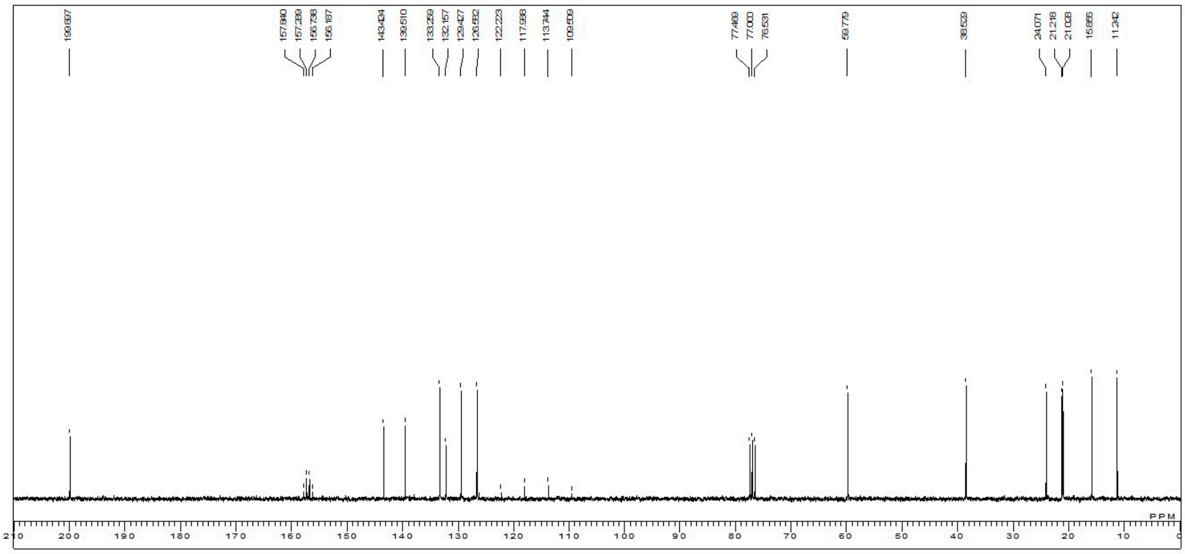

***N*-((2*S*,3*S*)-1-(2,5-Dimethylphenyl)-3-methyl-1-oxopentan-2-yl)-2,2,2-trifluoroacetamide (TFA-L-Ile- Ph(2,5-Me), L-1h)**

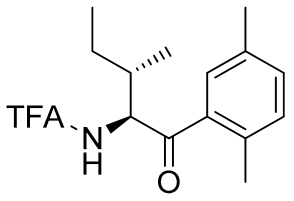

^{1}H NMR (270 MHz, $CDCl_3$)

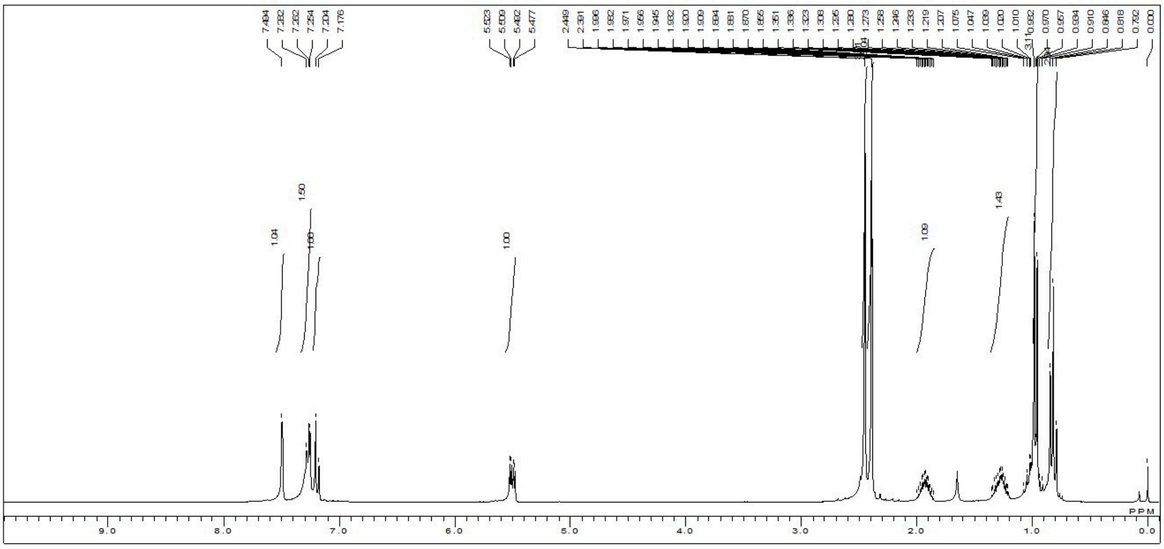

^{13}C NMR (67.5 MHz, $CDCl_3$)

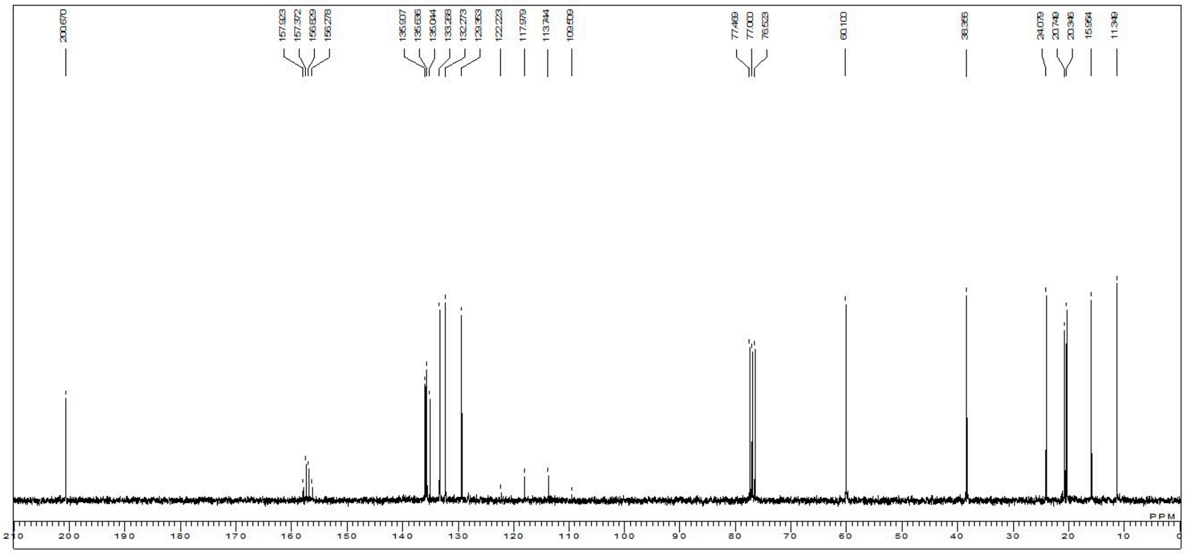

(2*S*,3*R*)-3-Methyl-2-(2,2,2-trifluoroacetamido)pentanoic acid (TFA-L-*allo*-Ile, L-2a)

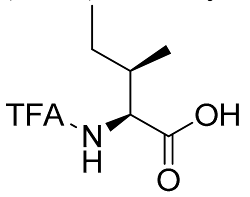

^1H NMR (270 MHz, $CDCl_3$)

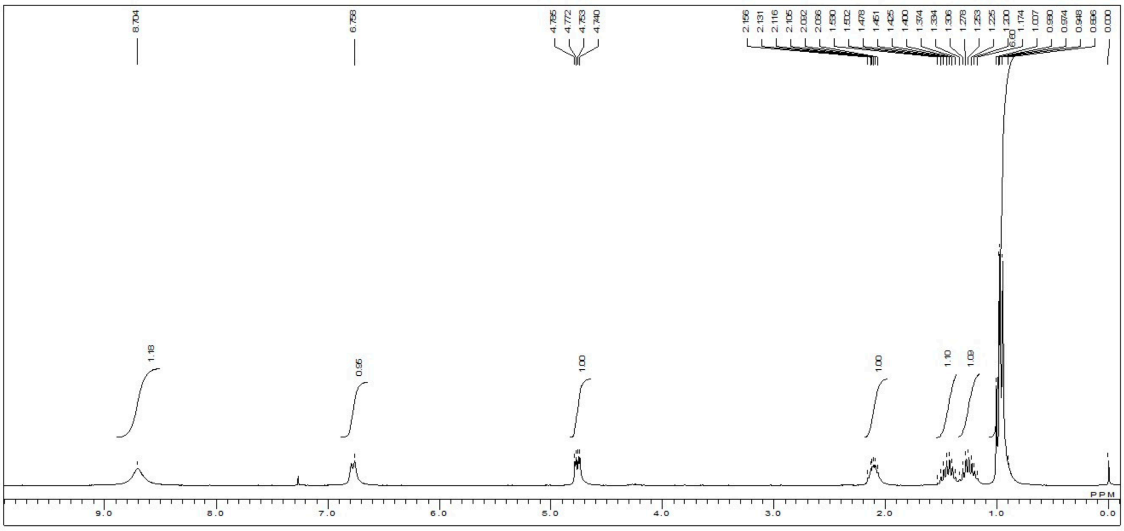

^{13}C NMR (67.5 MHz, $CDCl_3$)

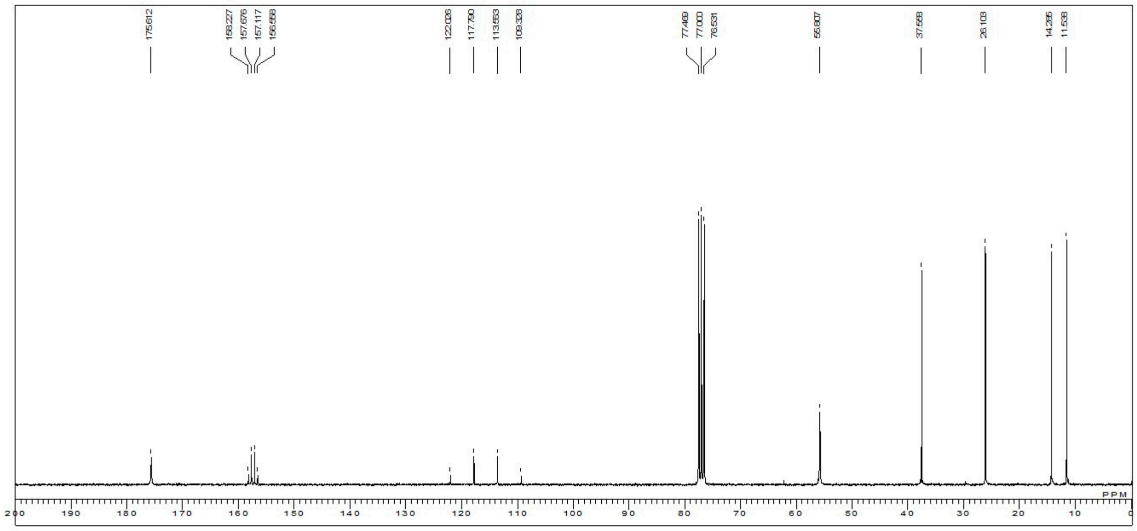

(2*R*,3*S*)-3-Methyl-2-(2,2,2-trifluoroacetamido)pentanoic acid (TFA-D-*allo*-Ile, D-2a)

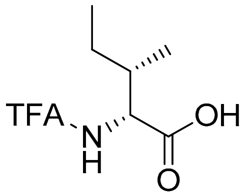

^{1}H NMR (270 MHz, $CDCl_3$)

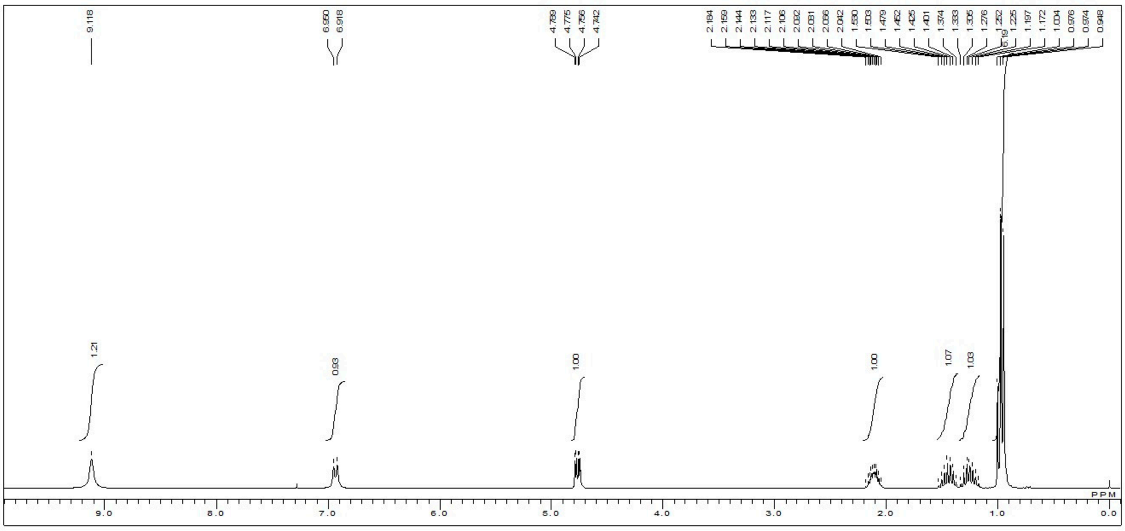

^{13}C NMR (67.5 MHz, $CDCl_3$)

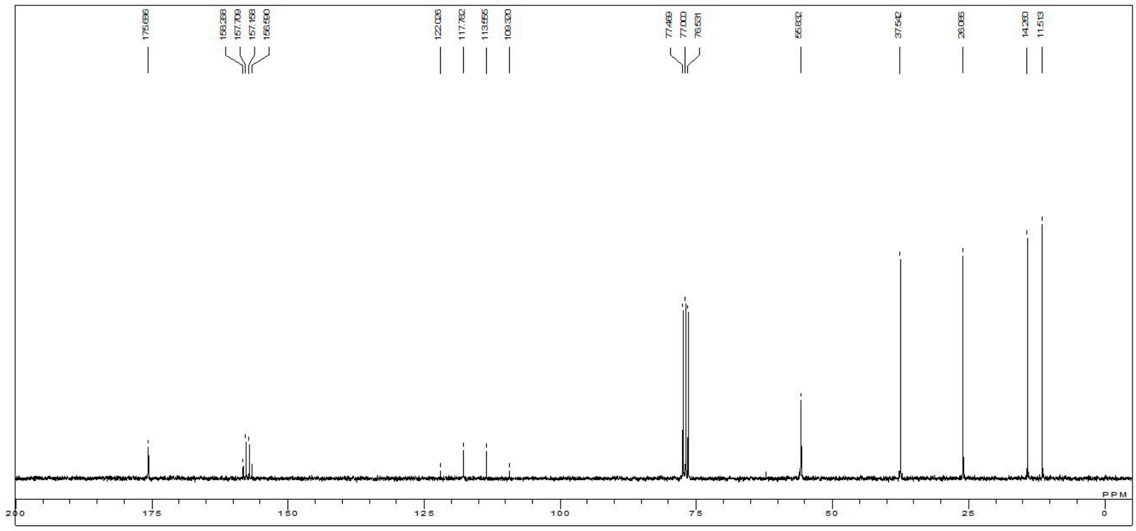

(2*S*,3*R*)-2,5-Dioxopyrrolidin-1-yl 3-methyl-2-(2,2,2-trifluoroacetamido)pentanoate (TFA-L-*allo*-Ile-OSu, L-2b)

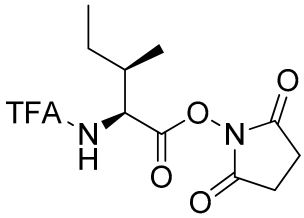

^{1}H NMR (270 MHz, $CDCl_3$)

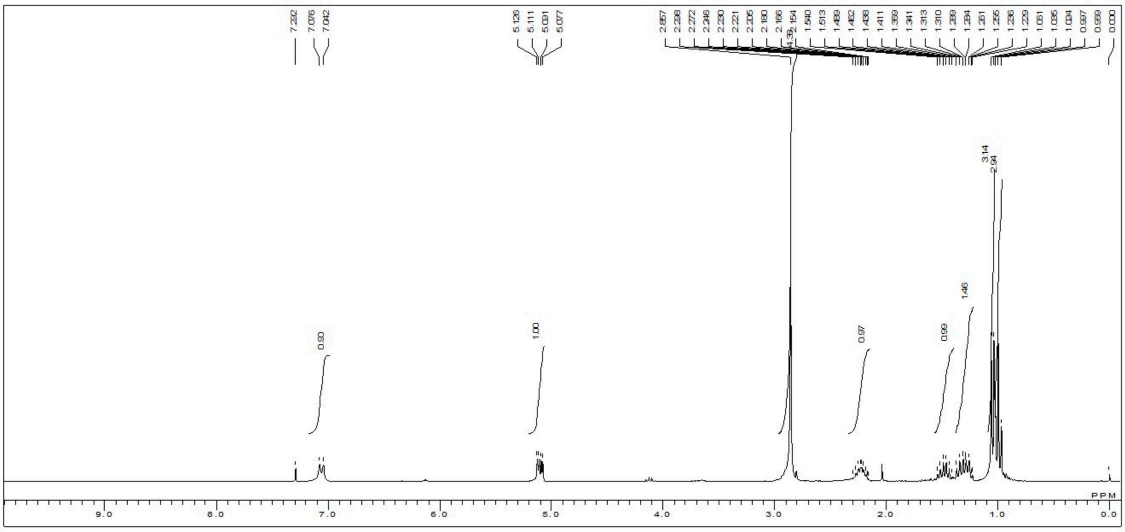

^{13}C NMR (67.5 MHz, $CDCl_3$)

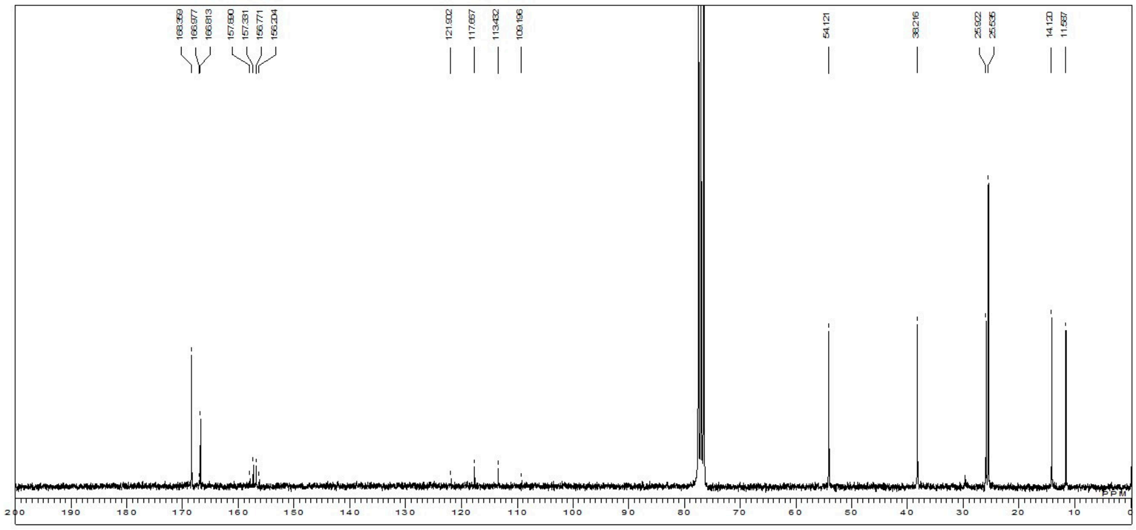

(2*R*,3*S*)-2,5-Dioxopyrrolidin-1-yl 3-methyl-2-(2,2,2-trifluoroacetamido)pentanoate (TFA-D-*allo*-Ile-OSu, D-2b)

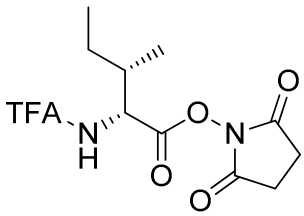

^1H NMR (270 MHz, $CDCl_3$)

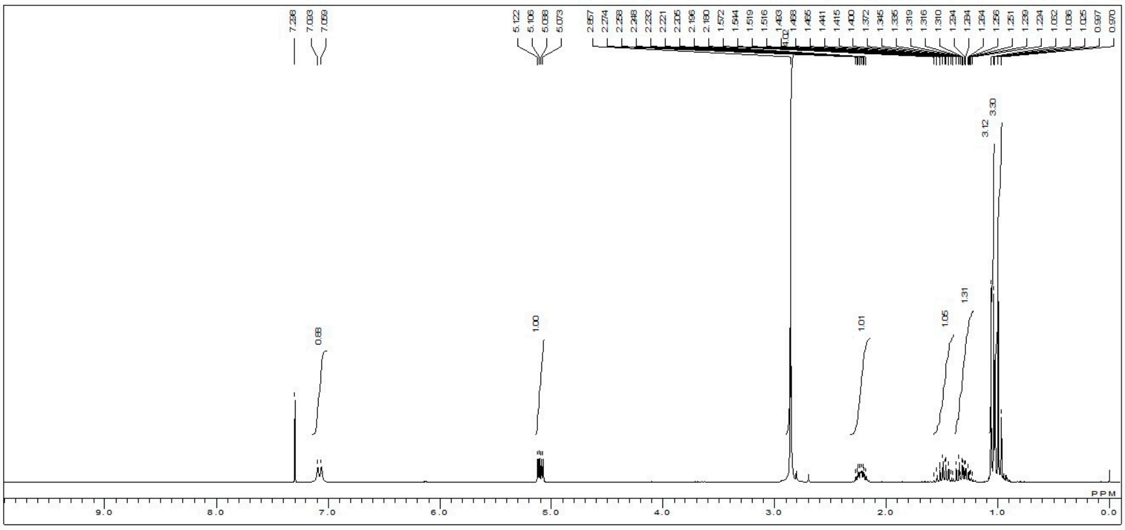

^{13}C NMR (67.5 MHz, $CDCl_3$)

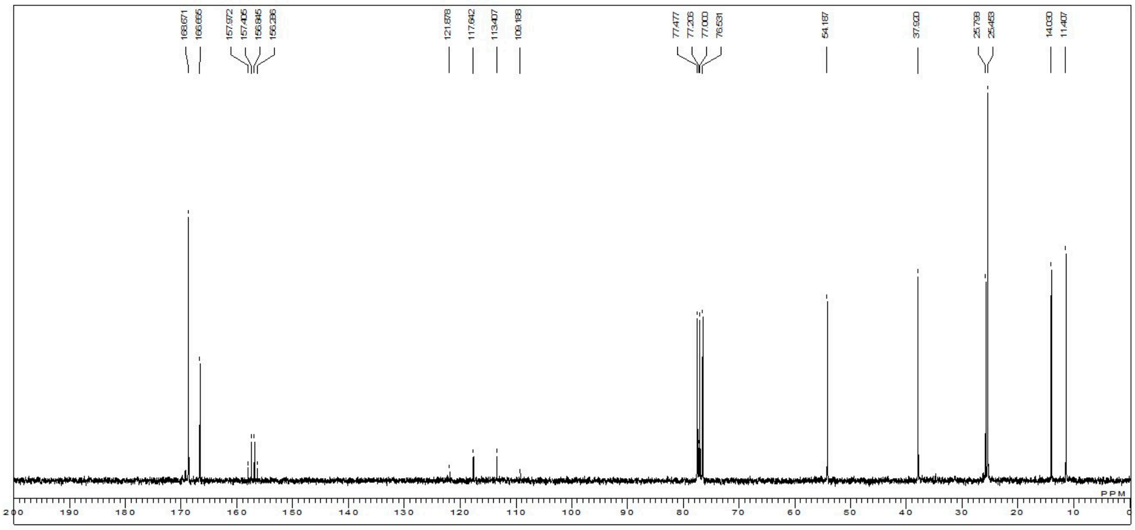

2,2,2-Trifluoro-*N*-((2*S*,3*R*)-3-methyl-1-oxo-1-phenylpentan-2-yl)acetamide (TFA-L-*allo*-Ile-Ph, L-2c)

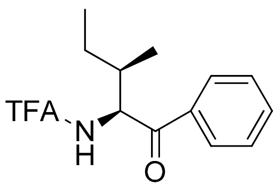

^{1}H NMR (270 MHz, CDCl$_3$)

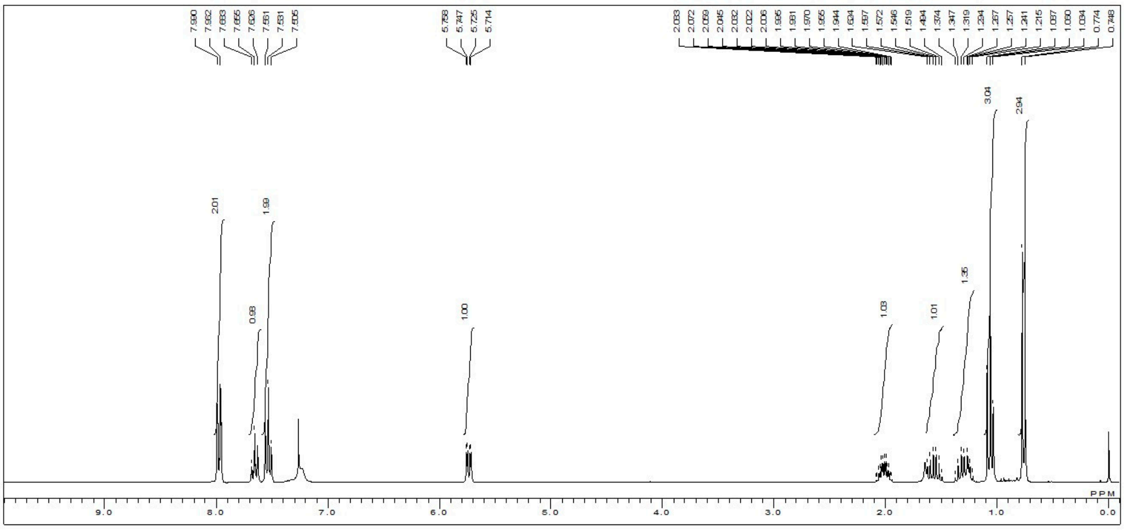

^{13}C NMR (67.5 MHz, CDCl$_3$)

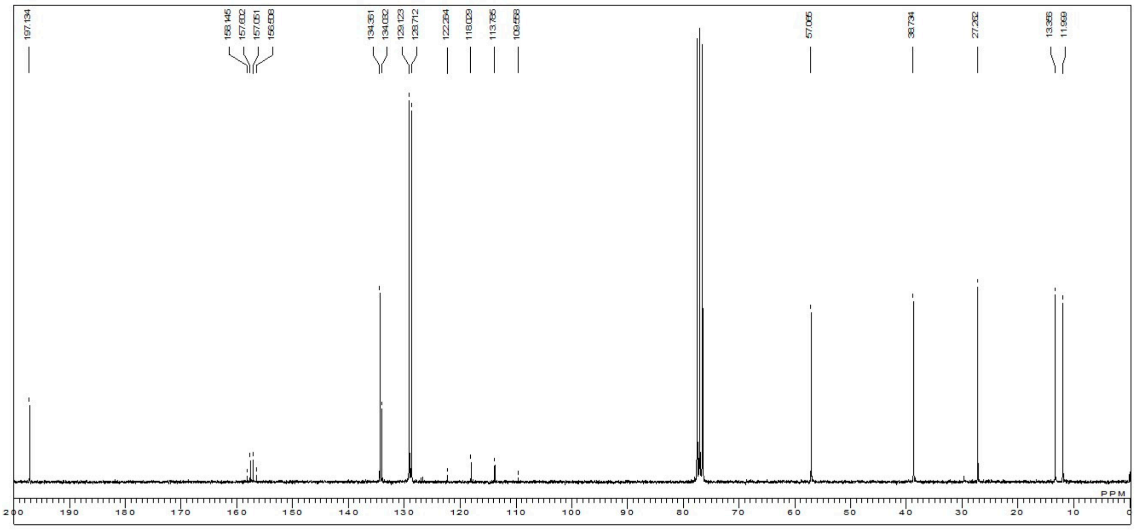

2,2,2-Trifluoro-*N*-((2*R*,3*S*)-3-methyl-1-oxo-1-phenylpentan-2-yl)acetamide (TFA-D-*allo*-Ile-Ph, D-2c)

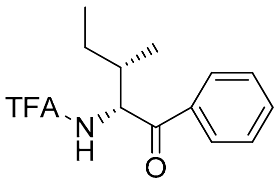

^{1}H NMR (270 MHz, $CDCl_3$)

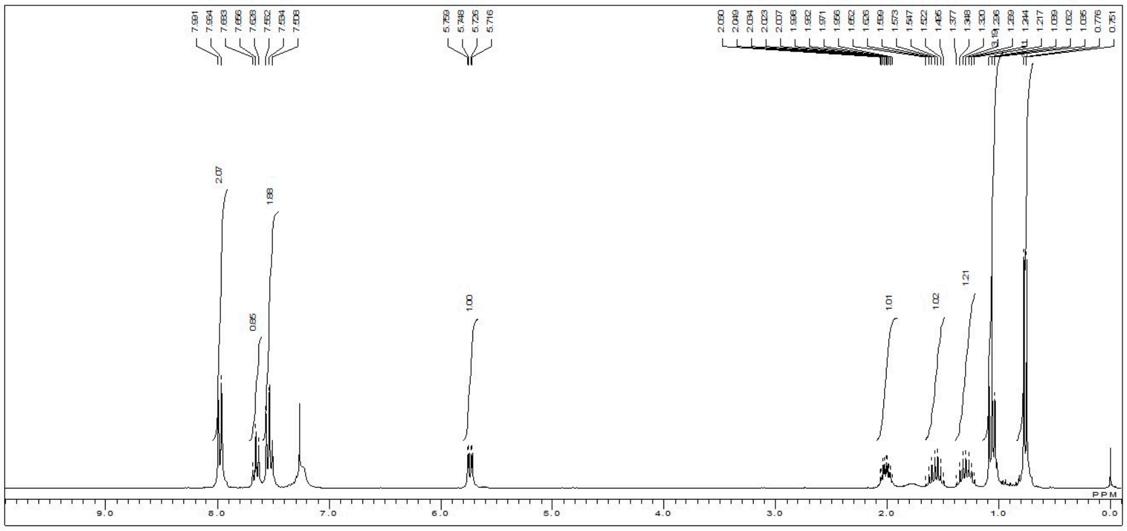

^{13}C NMR (67.5 MHz, $CDCl_3$)

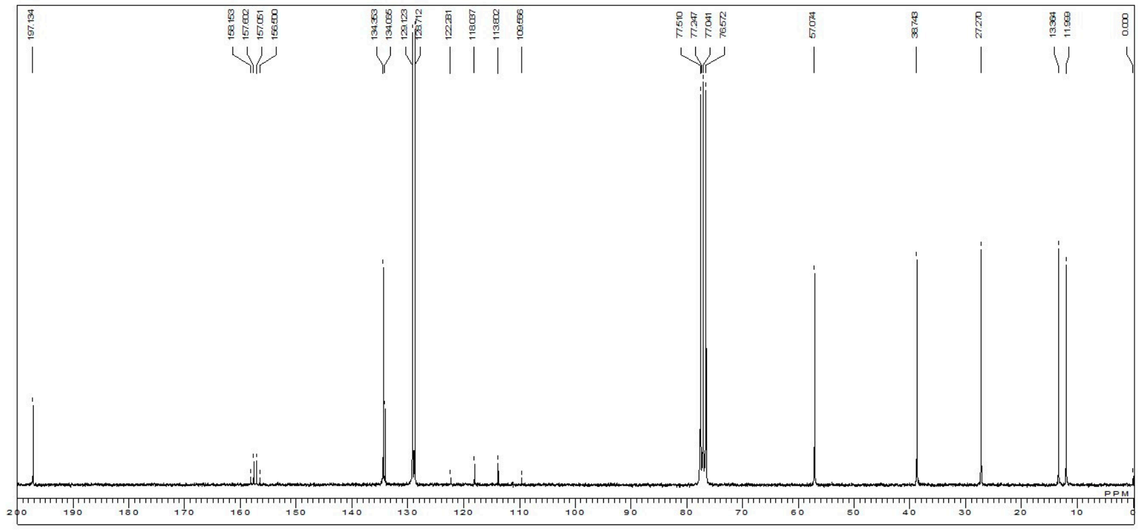

2-(2,2,2-Trifluoroacetamido)acetic acid (TFA-Gly, 3a)

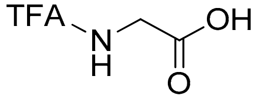

^{1}H-NMR (270 MHz, CD_3OD)

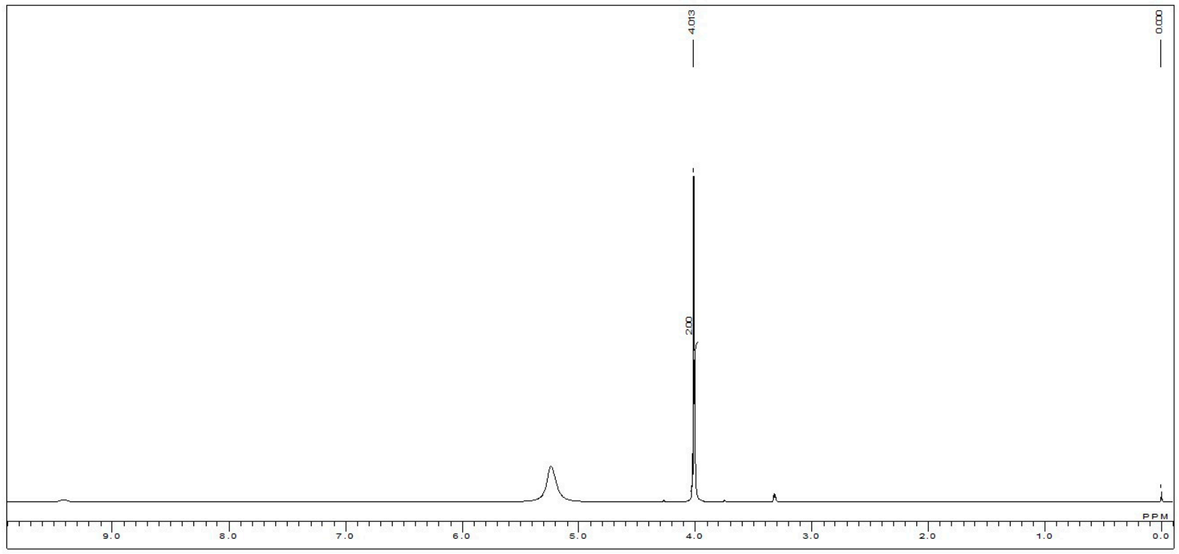

^{13}C NMR (67.5 MHz, CD_3OD)

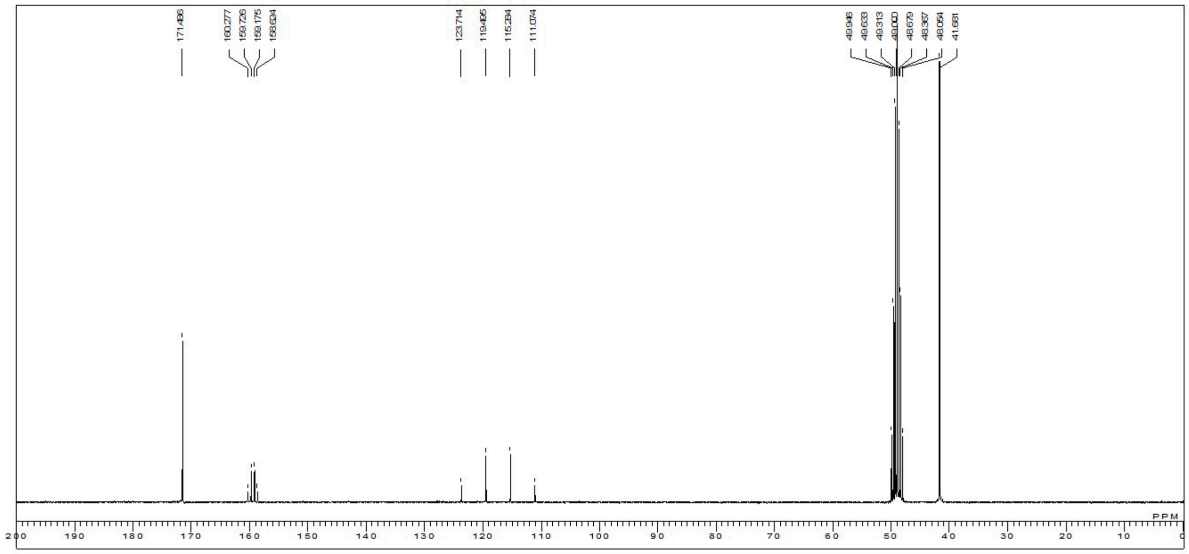

2,5-Dioxocyclopentyl 2-(2,2,2-trifluoroacetamido)acetate (TFA-Gly-OSu, 3b)

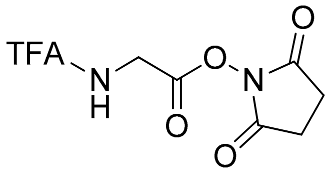

^{1}H-NMR (270 MHz, CD_3OD)

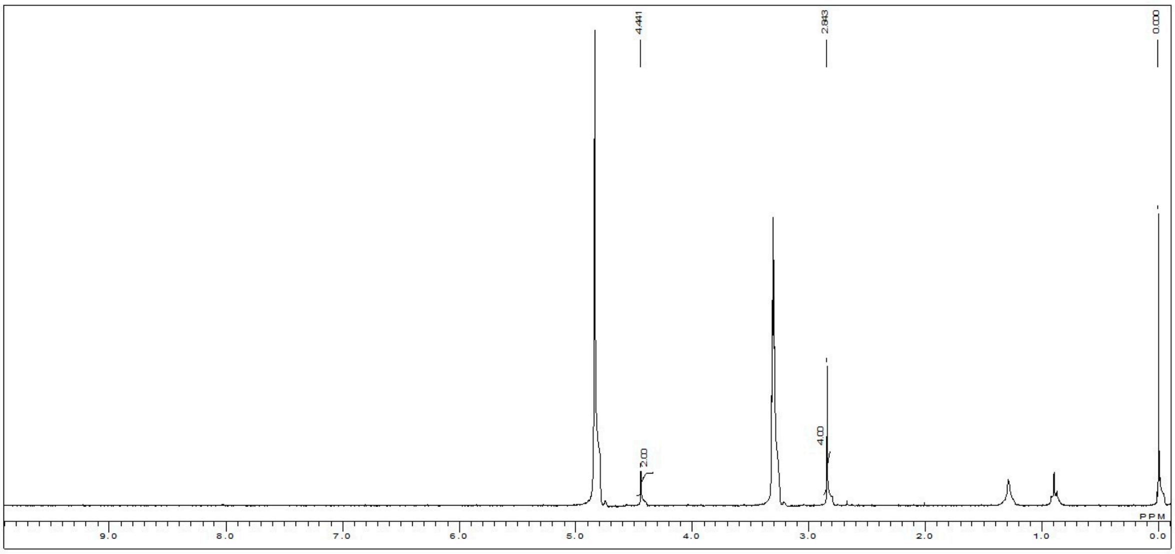

^{13}C NMR (67.5 MHz, ACETONE-D_6)

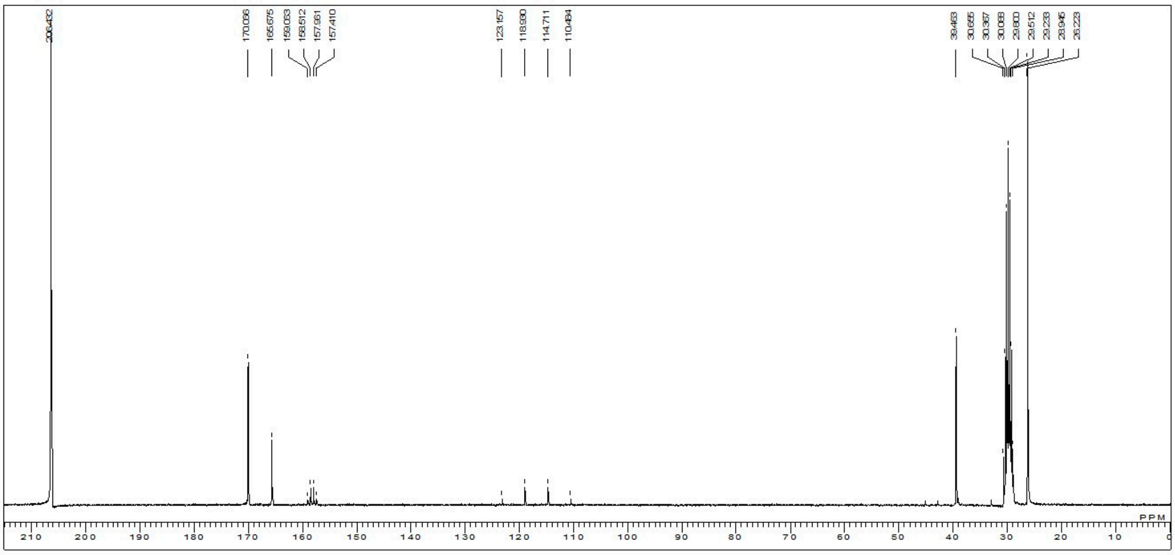

2,2,2-Trifluoro-*N*-(2-oxo-2-phenylethyl)acetamide (TFA-Gly-Ph, 3c)

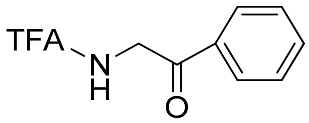

^{1}H-NMR (270 MHz, $CHCl_3$)

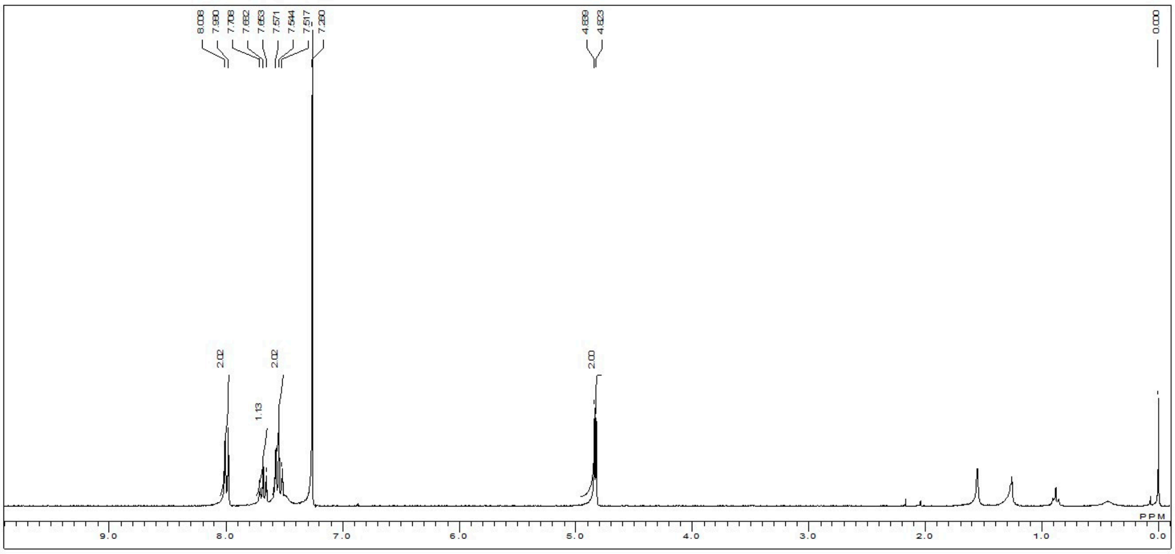

^{13}C NMR (67.5 MHz, $CHCl_3$)

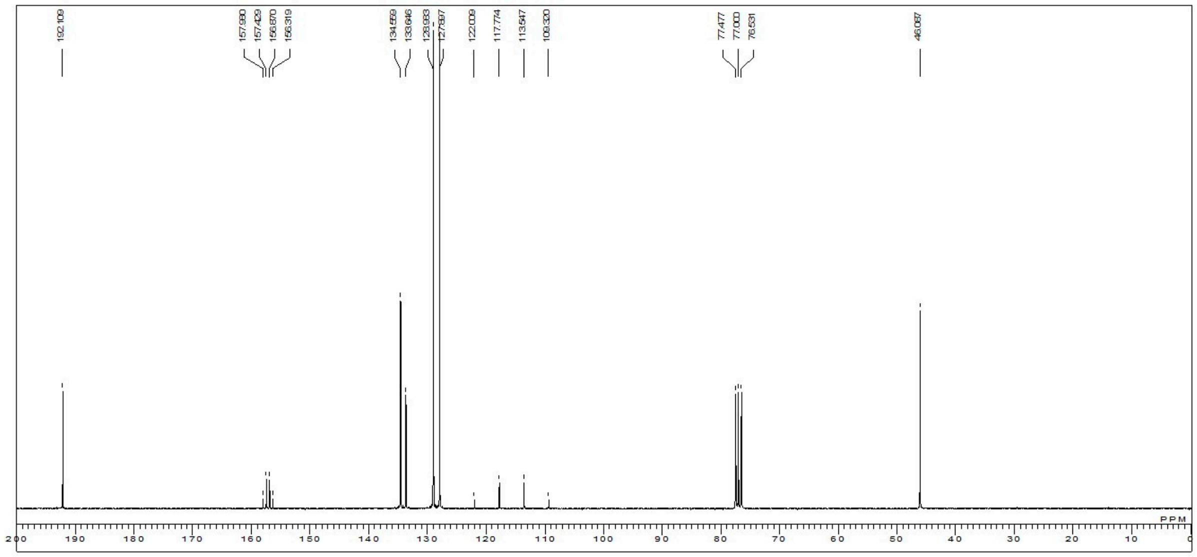

(*S*)-2-(2,2,2-Trifluoroacetamido)propanoic acid (TFA-L-Ala, L-4a)

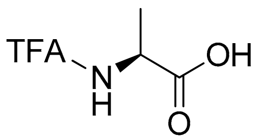

^{1}H NMR (270 MHz, $CDCl_3$)

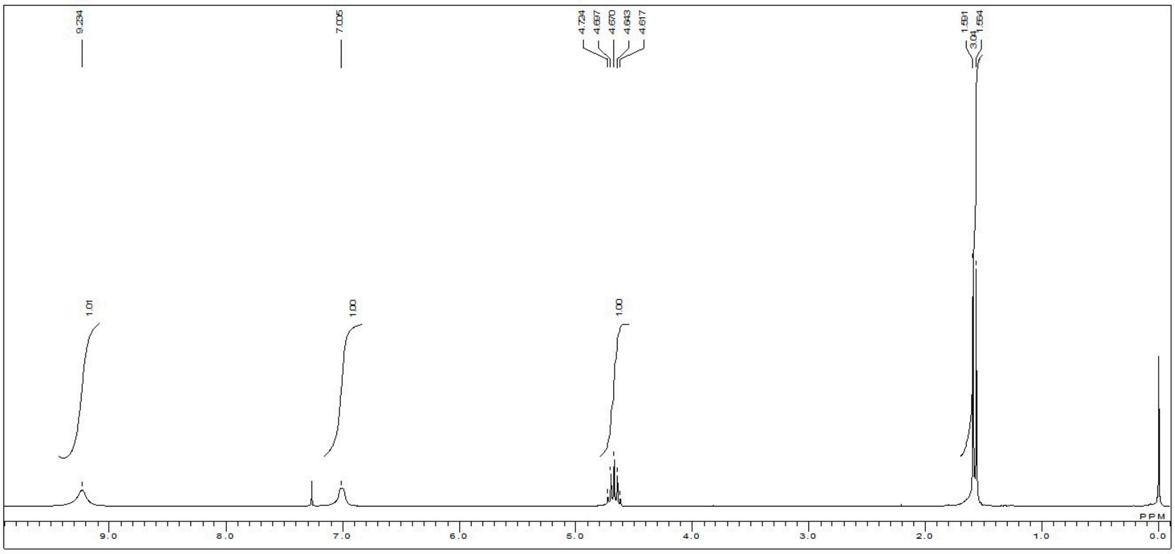

^{13}C NMR (67.5 MHz, $CDCl_3$)

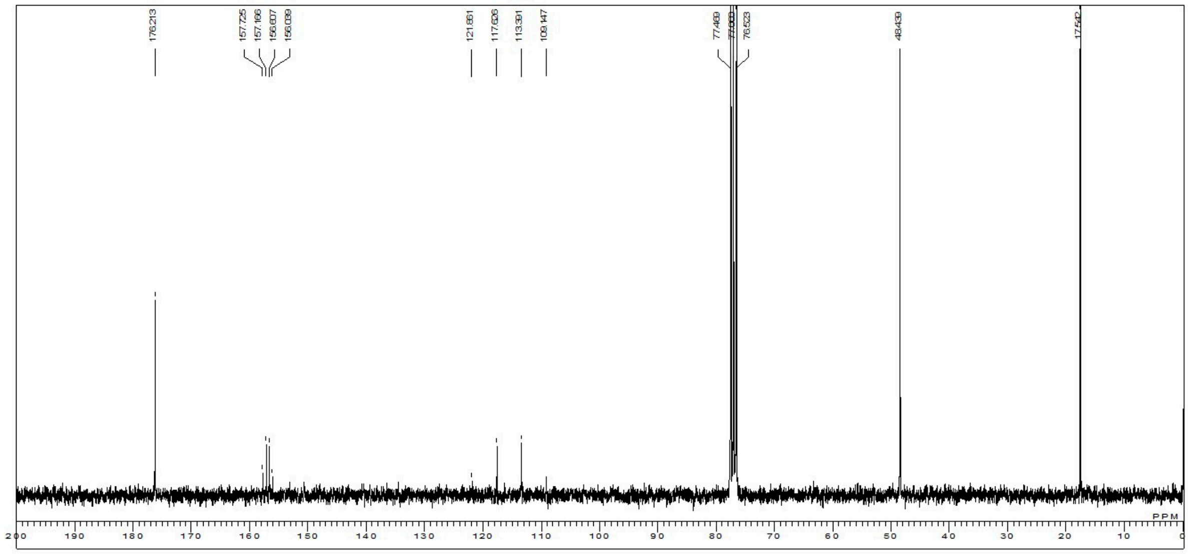

(*R*)-2-(2,2,2-Trifluoroacetamido)propanoic acid (TFA-D-Ala, D-4a)

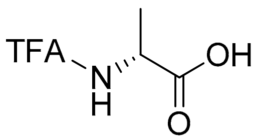

^{1}H NMR (270 MHz, $CDCl_3$)

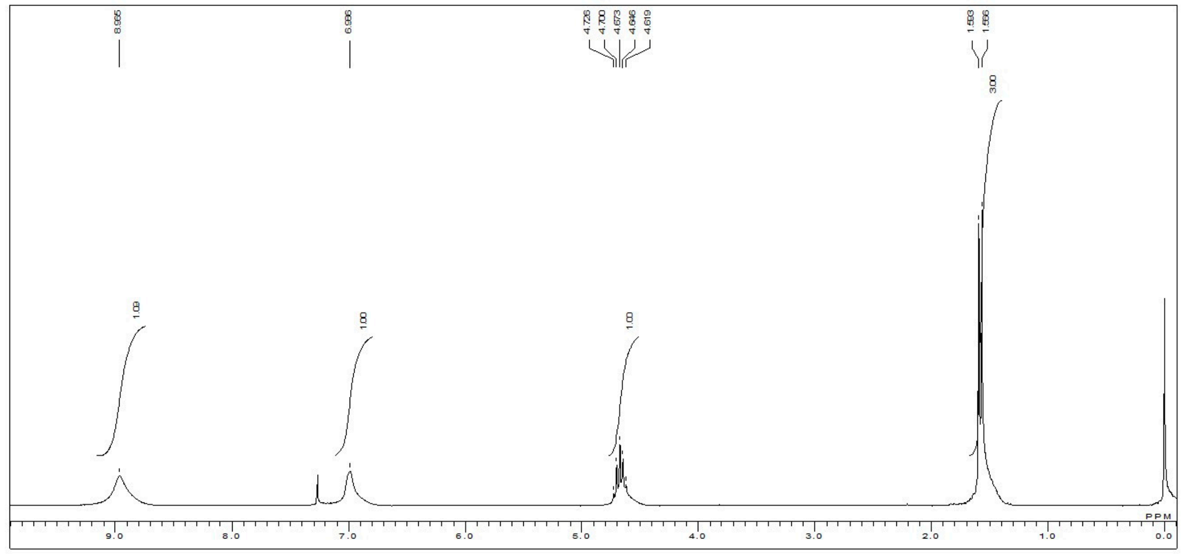

^{13}C NMR (67.5 MHz, $CDCl_3$)

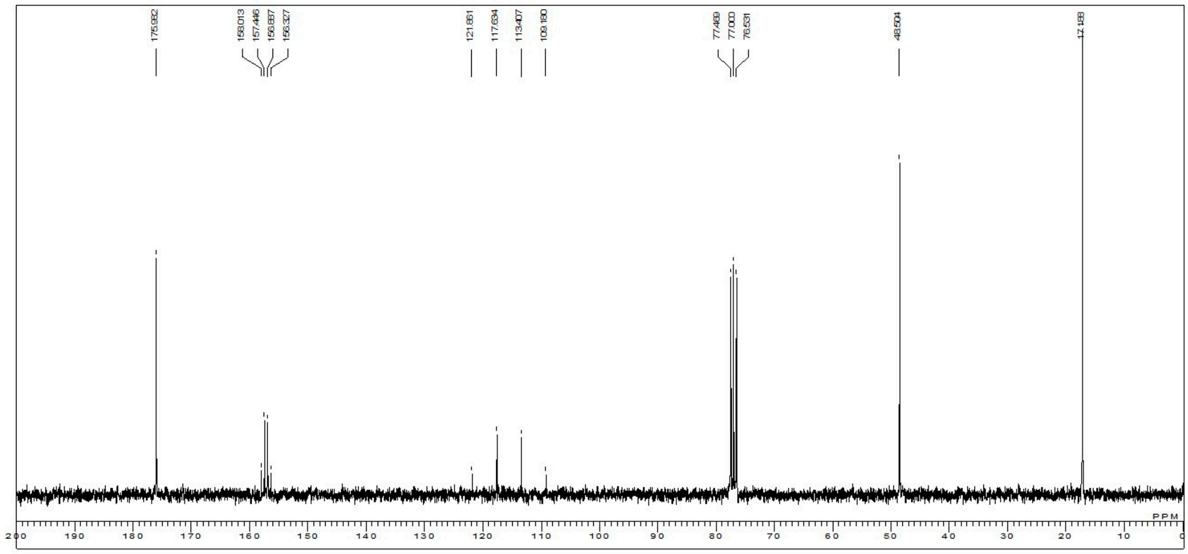

(*S*)-2,5-Dioxopyrrolidin-1-yl 2-(2,2,2-trifluoroacetamido)propanoate (TFA-L-Ala-OSu, L-4b)

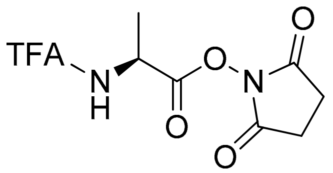

^{1}H NMR (270 MHz, $CDCl_3$)

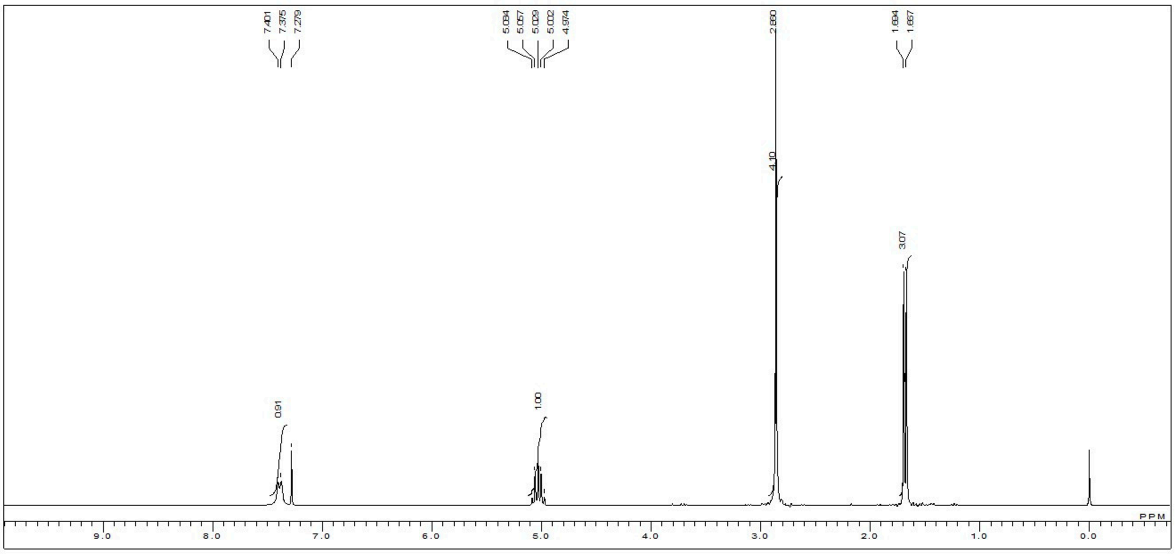

^{13}C NMR (67.5 MHz, $CDCl_3$)

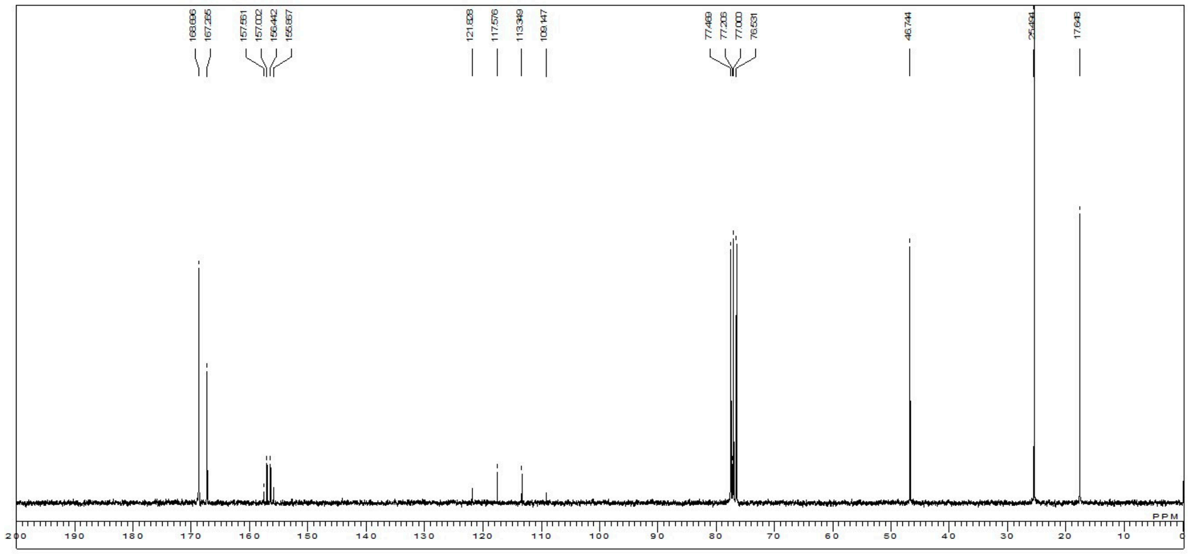

(*R*)-2,5-Dioxopyrrolidin-1-yl 2-(2,2,2-trifluoroacetamido)propanoate (TFA-D-Ala-OSu, D-4b)

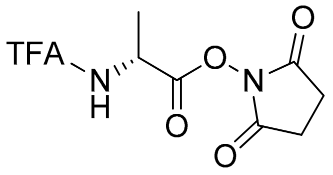

^{1}H NMR (270 MHz, $CDCl_3$)

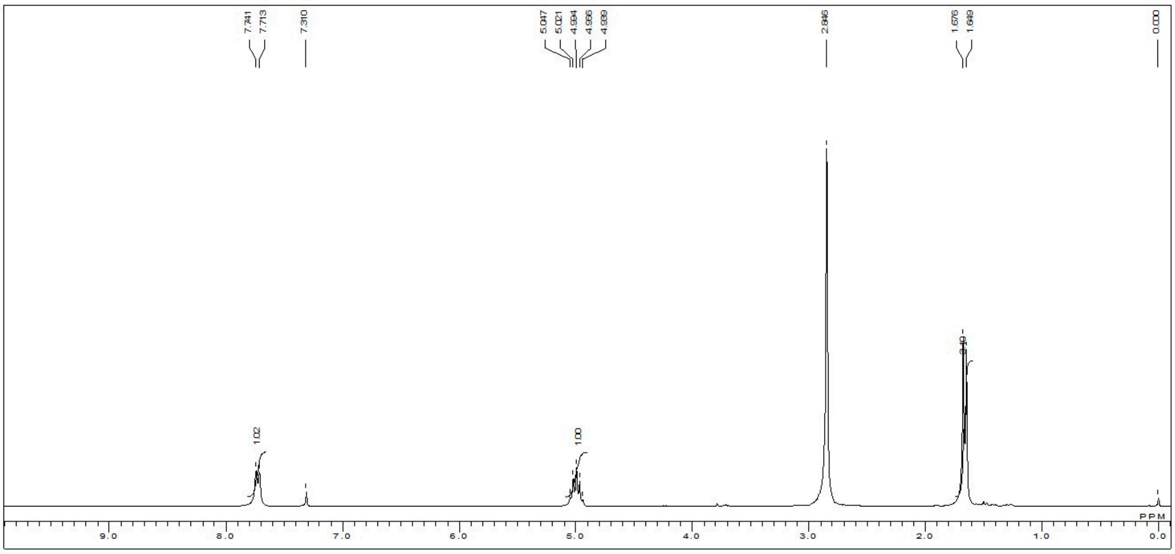

^{13}C NMR (67.5 MHz, $CDCl_3$)

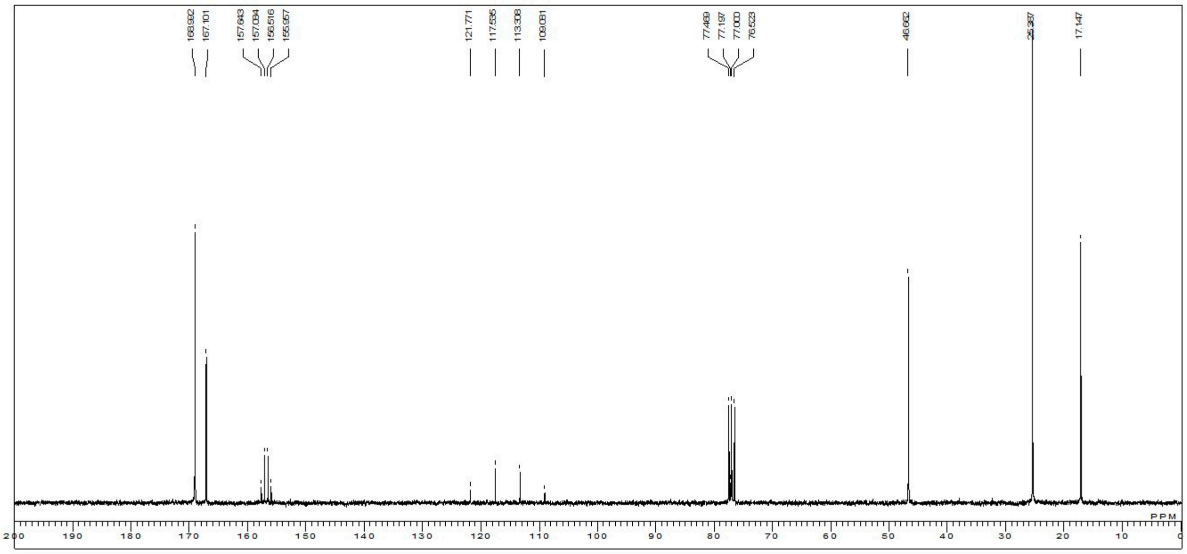

(*S*)-2,2,2-trifluoro-*N*-(1-oxo-1-phenylpropan-2-yl)acetamide (TFA-L-Ala-Ph, L-4c)

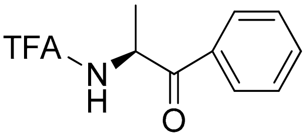

^{1}H NMR (270 MHz, $CDCl_3$)

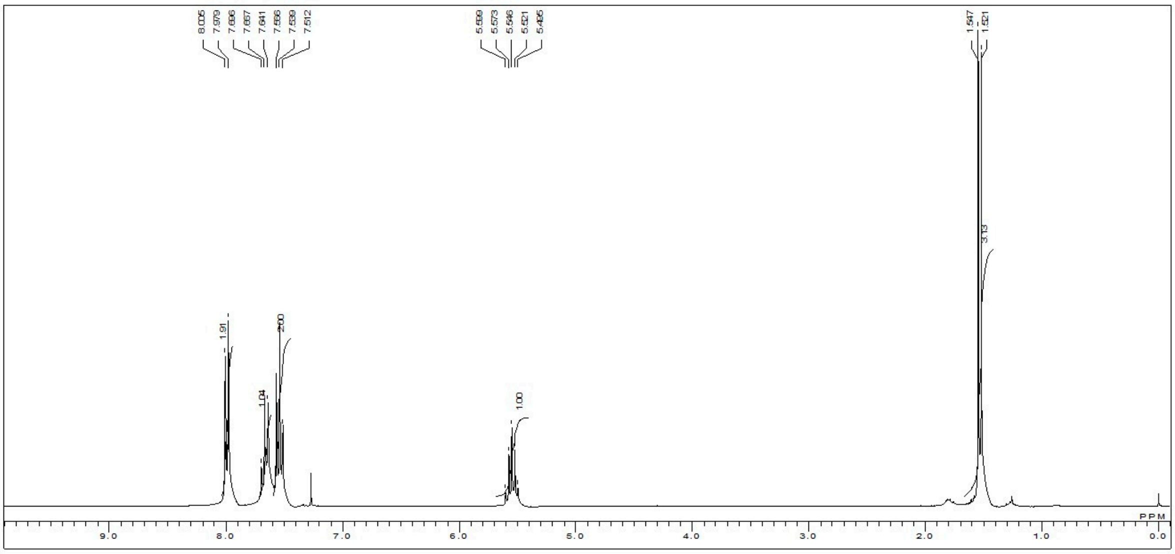

^{13}C NMR (67.5 MHz, $CDCl_3$)

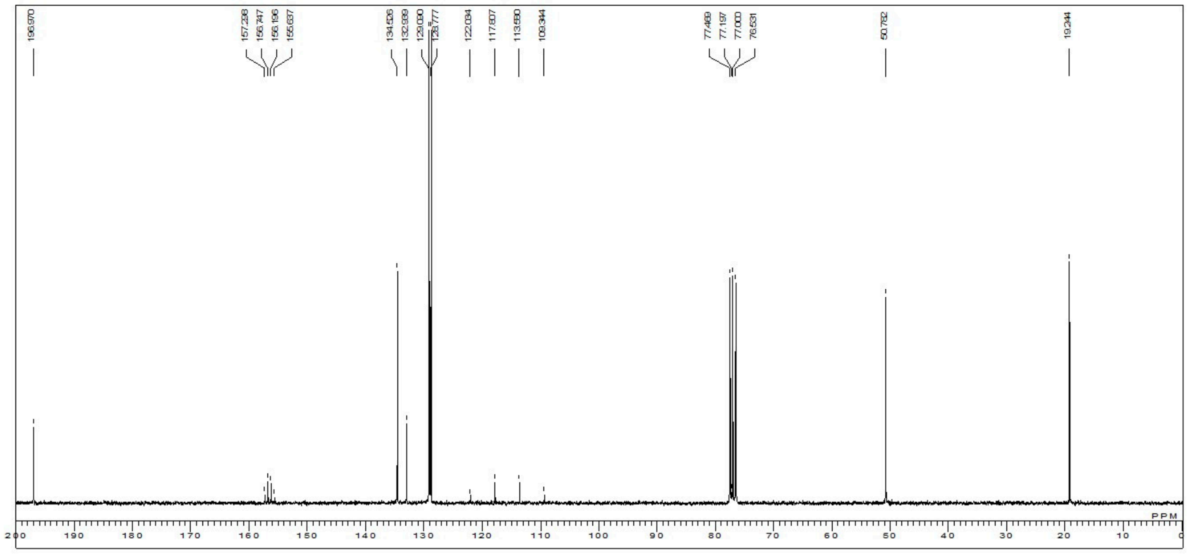

(*R*)-2,2,2-Trifluoro-*N*-(1-oxo-1-phenylpropan-2-yl)acetamide (TFA-D-Ala-Ph, D-4c)

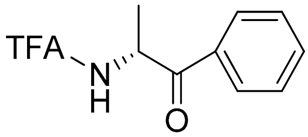

^{1}H NMR (270 MHz, $CDCl_3$)

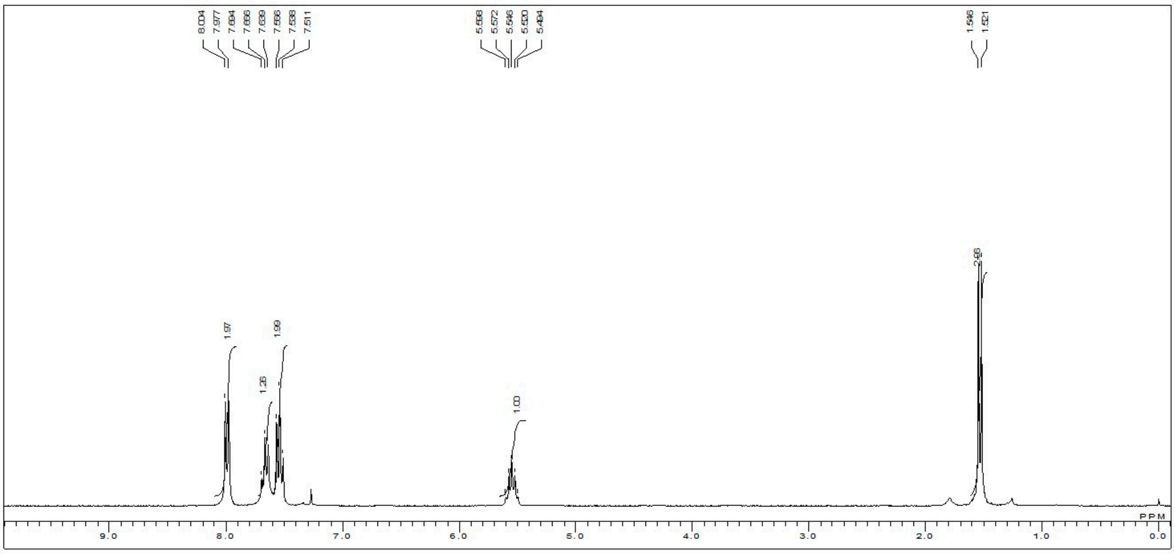

^{13}C NMR (67.5 MHz, $CDCl_3$)

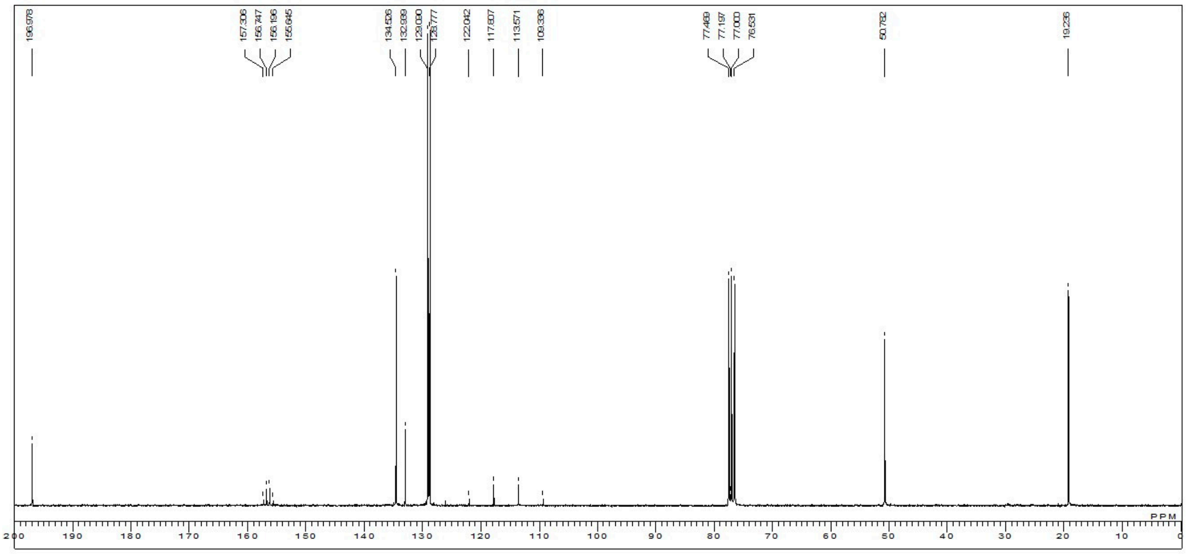

(*S*)-3-Methyl-2-(2,2,2-trifluoroacetamido)butanoic acid (TFA-L-Val, L-5a)

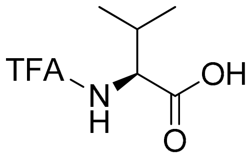

^{1}H-NMR (270 MHz, CD_3Cl_3)

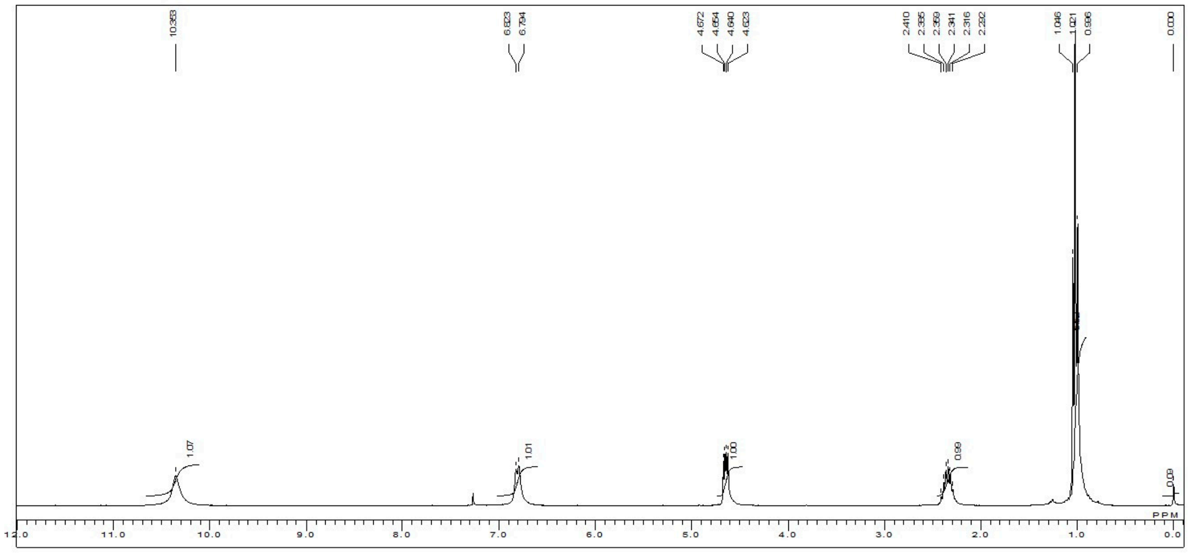

^{13}C-NMR (67.5 MHz, $CDCl_3$)

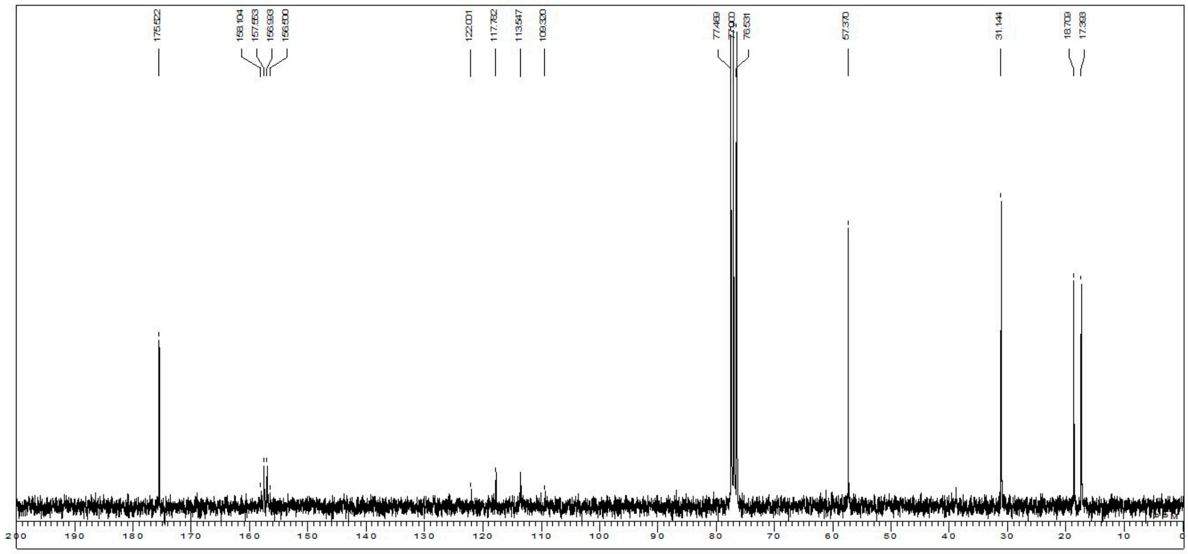

(*R*)-3-Methyl-2-(2,2,2-trifluoroacetamido)butanoic acid (TFA-D-Val, D-5a)

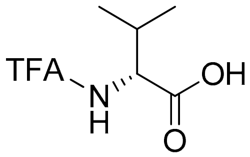

^{1}H-NMR (270 MHz, CD_3Cl_3)

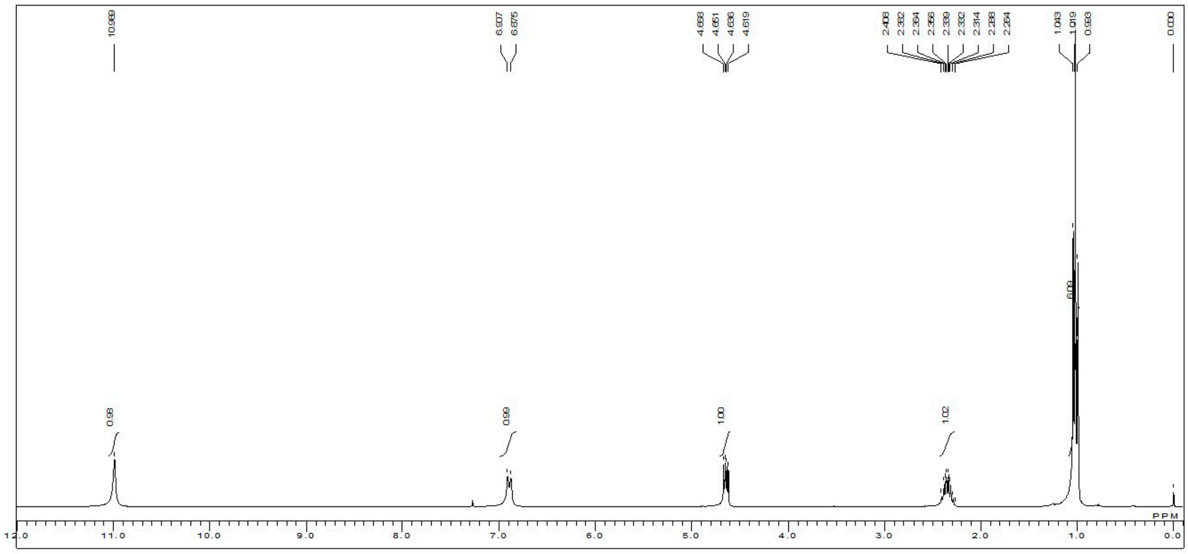

^{13}C-NMR (67.5 MHz, $CDCl_3$)

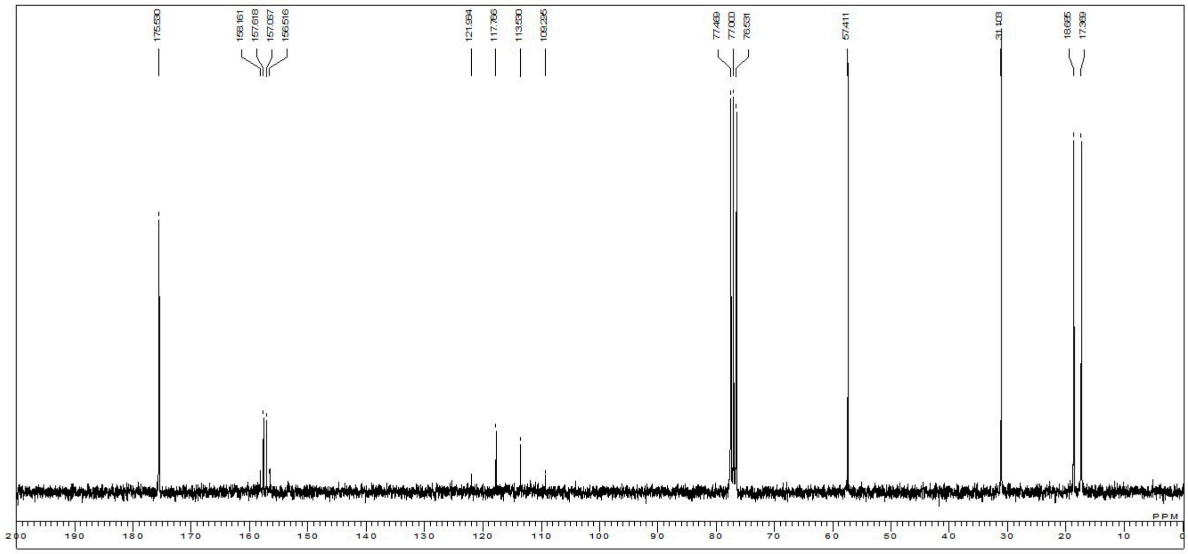

(*S*)-2,5-Dioxopyrrolidin-1-yl 3-methyl-2-(2,2,2-trifluoroacetamido)butanoate (TFA-L-Val-OSu, L-5b)

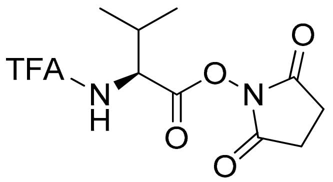

^{1}H-NMR (270 MHz, CD_3Cl_3)

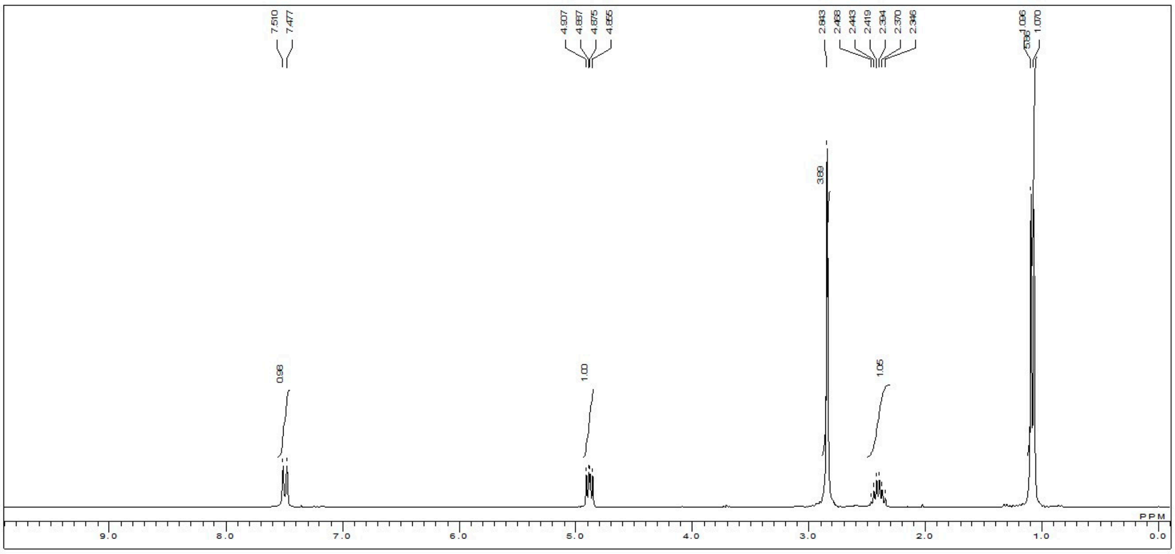

^{13}C-NMR (67.5 MHz, $CDCl_3$)

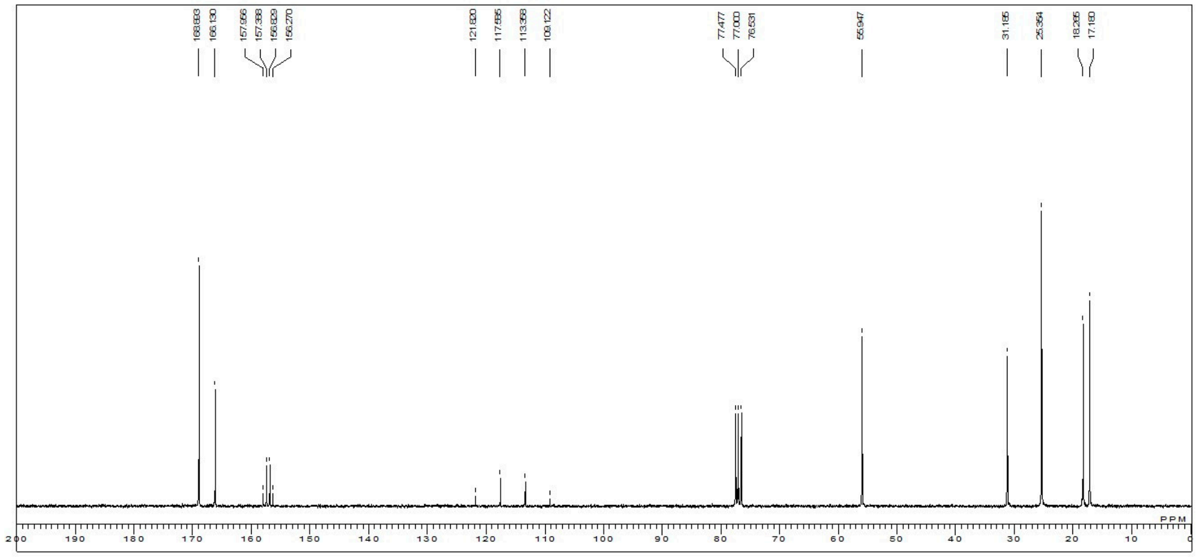

(*R*)-2,5-Dioxopyrrolidin-1-yl 3-methyl-2-(2,2,2-trifluoroacetamido)butanoate (TFA-D-Val-OSu, D-5b)

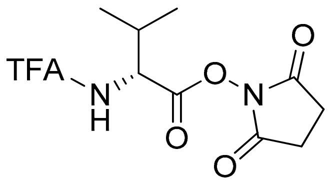

^{1}H-NMR (270 MHz, CD_3Cl_3)

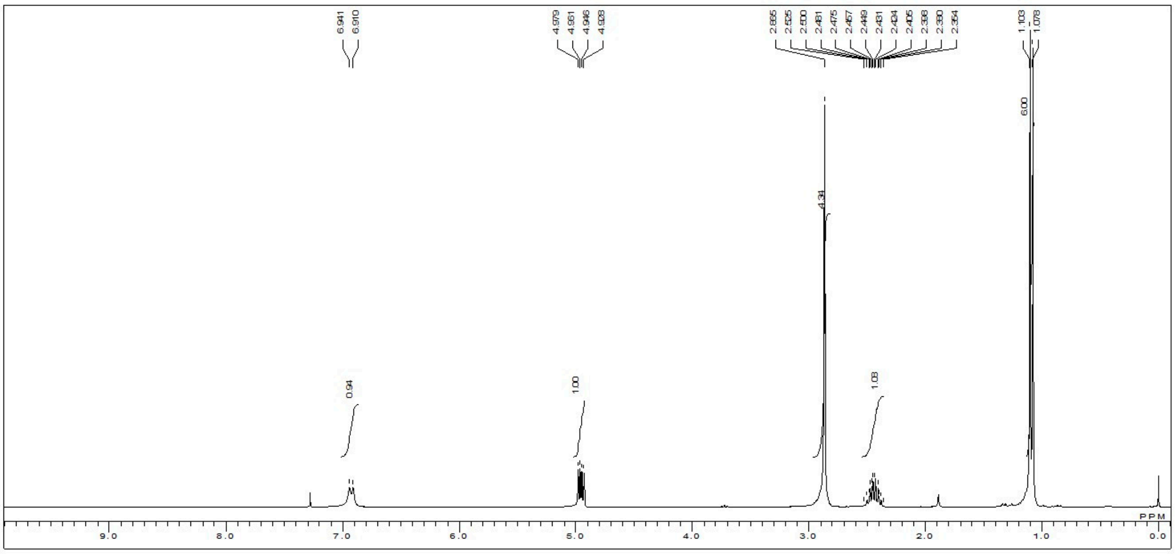

^{13}C-NMR (67.5 MHz, $CDCl_3$)

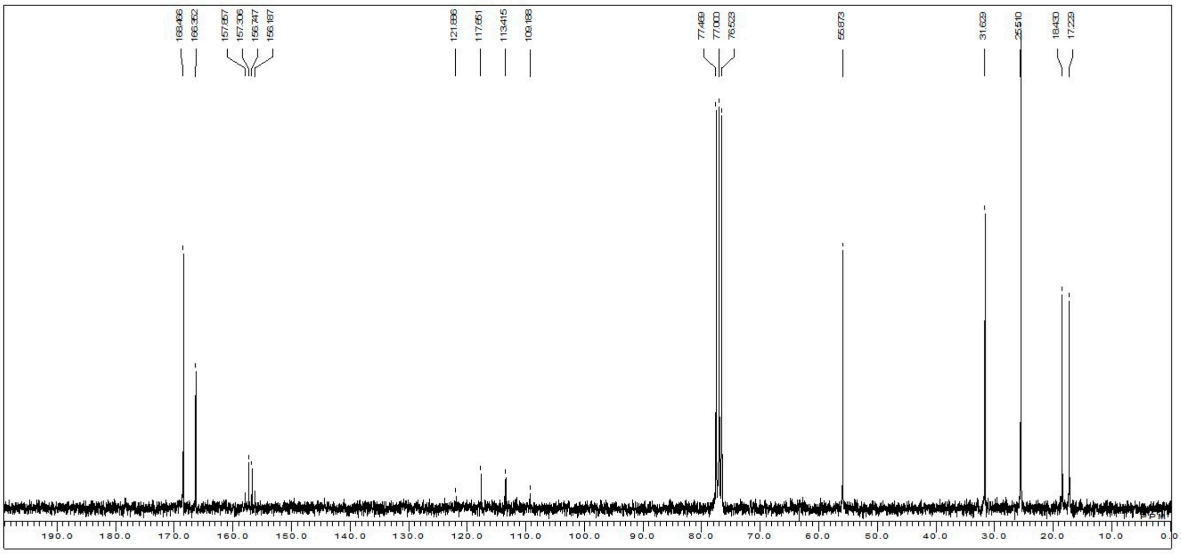

S)-2,2,2-Trifluoro-*N*-(3-methyl-1-oxo-1-phenylbutan-2-yl)acetamide (TFA-L-Val-Ph, L-5c)

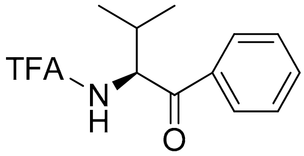

^{1}H-NMR (270 MHz, $CDCl_3$)

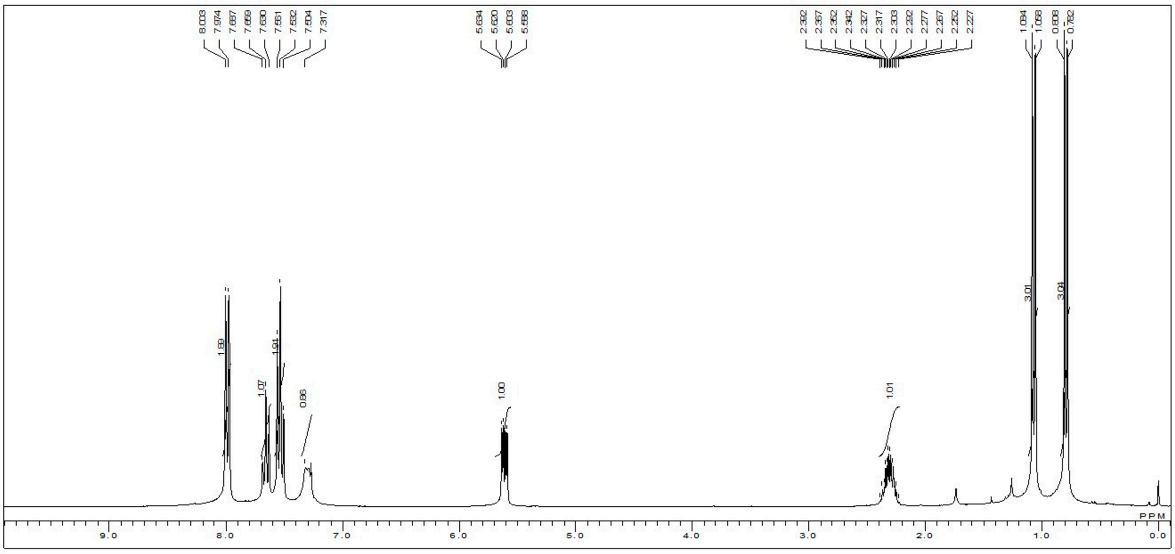

^{13}C-NMR (67.5 MHz, $CDCl_3$)

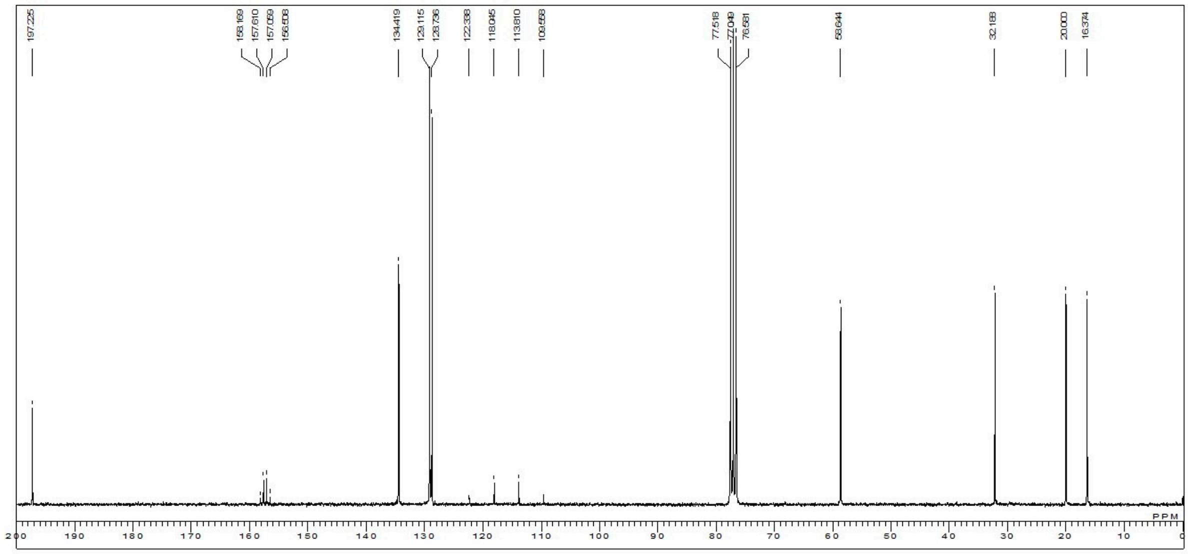

(*R*)-2,2,2-Trifluoro-*N*-(3-methyl-1-oxo-1-phenylbutan-2-yl)acetamide (TFA-D-Val-Ph, D-5c)

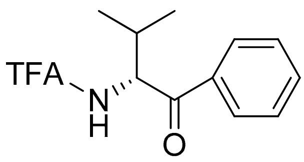

^{1}H-NMR (270 MHz, $CDCl_3$)

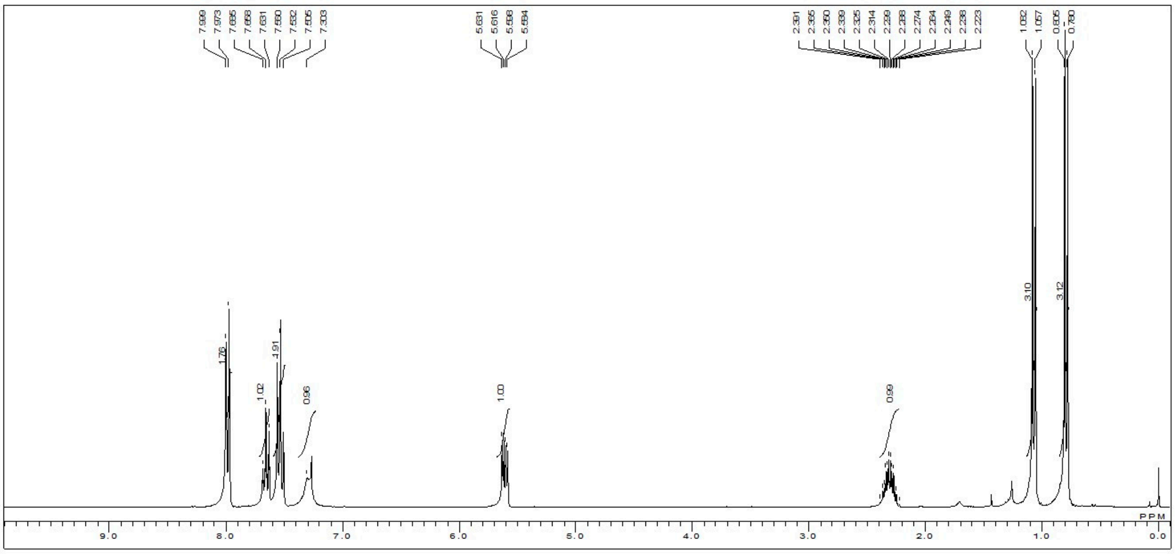

^{13}C-NMR (67.5 MHz, $CDCl_3$)

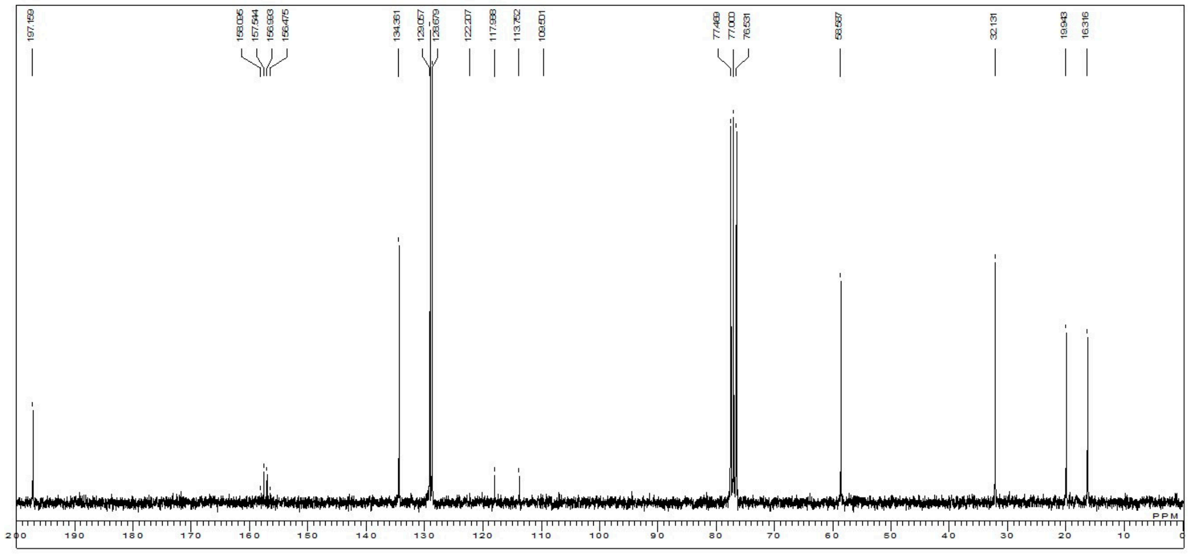

(*S*)-4-methyl-2-(2,2,2-trifluoroacetamido)pentanoic acid (TFA-L-Leu, L-6a)

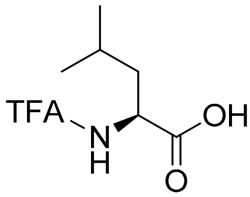

^{1}H-NMR (270 MHz, CD_3Cl_3)

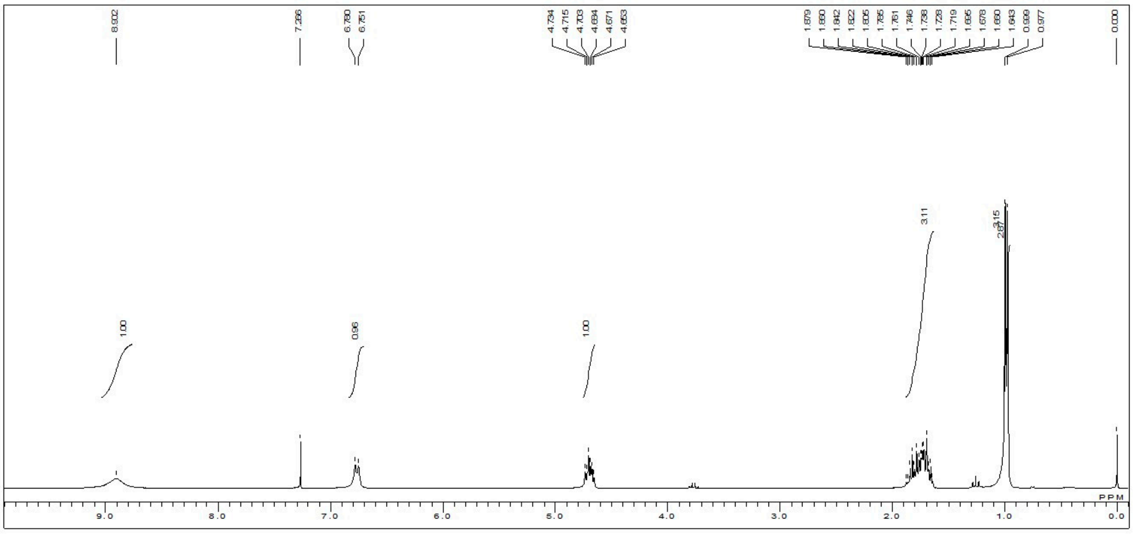

^{13}C-NMR (67.5 MHz, $CDCl_3$)

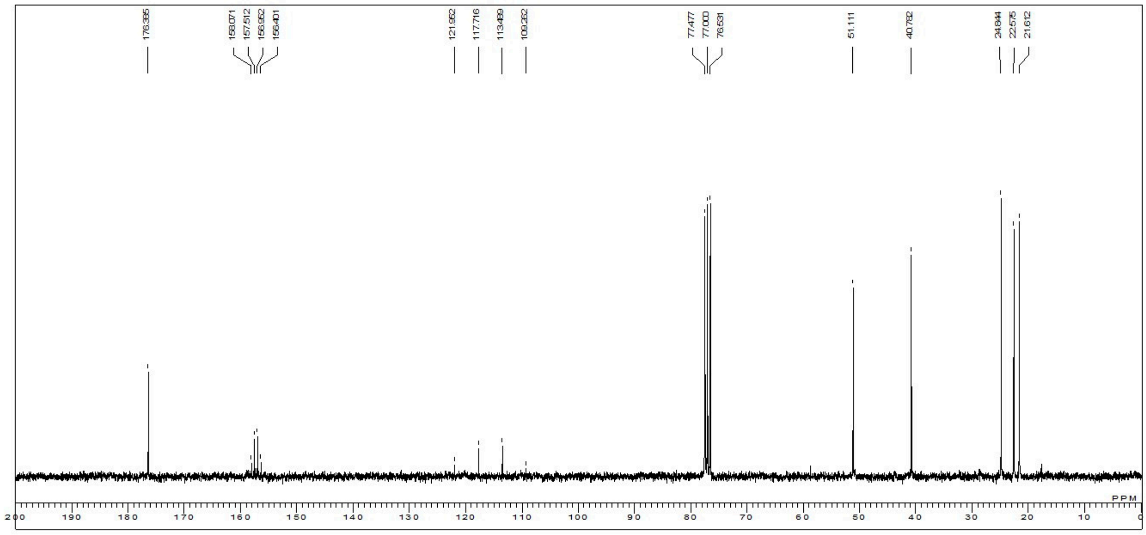

(*R*)-4-Methyl-2-(2,2,2-trifluoroacetamido)pentanoic acid (TFA-D-Leu, D-6a)

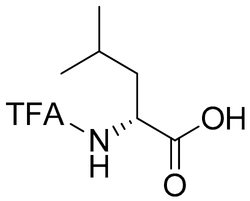

^{1}H-NMR (270 MHz, CD3Cl3)

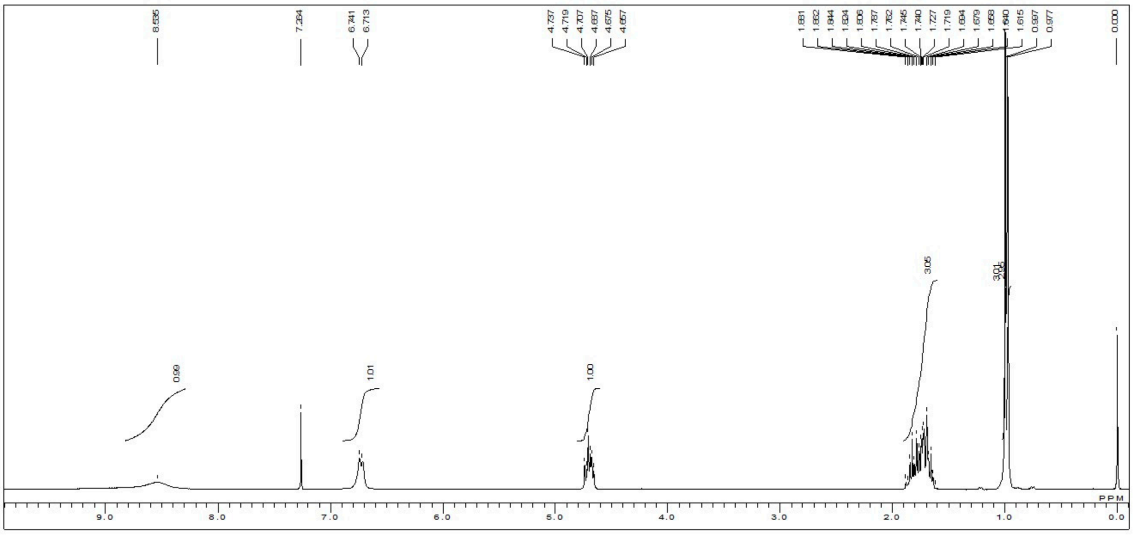

^{13}C-NMR (67.5 MHz, CDCl3)

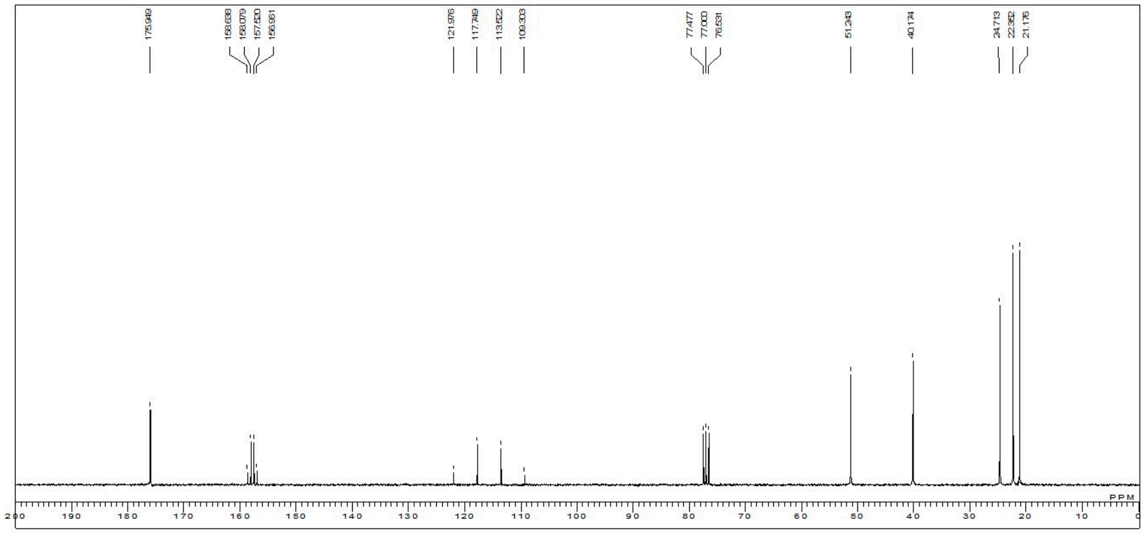

(*S*)-2,5-Dioxopyrrolidin-1-yl 4-methyl-2-(2,2,2-trifluoroacetamido)pentanoate (TFA-L-Leu-OSu, L-6b)

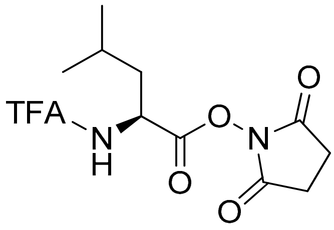

^{1}H-NMR (270 MHz, CD3Cl3)

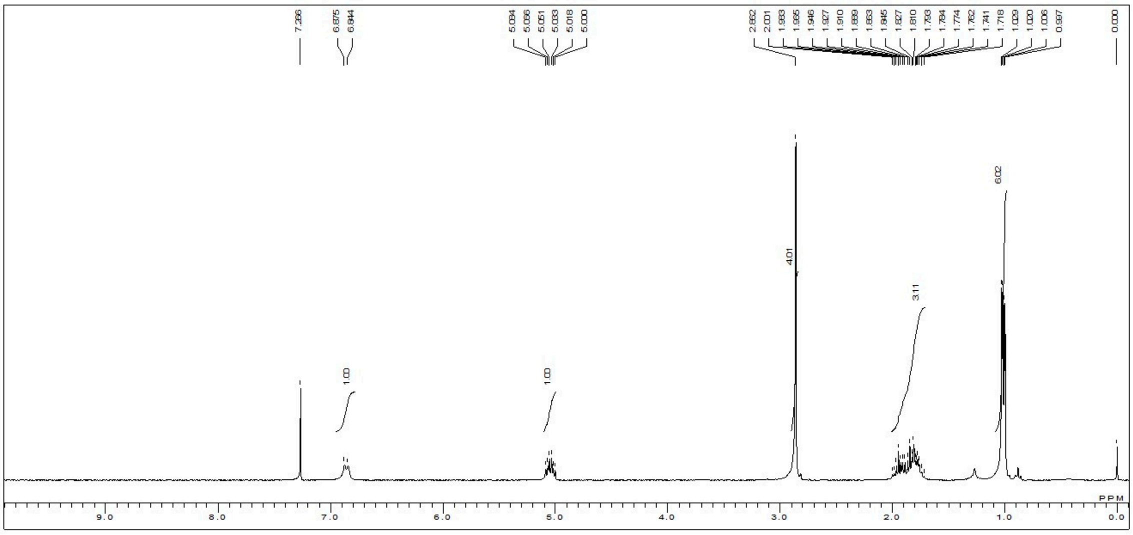

^{13}C-NMR (67.5 MHz, CDCl3)

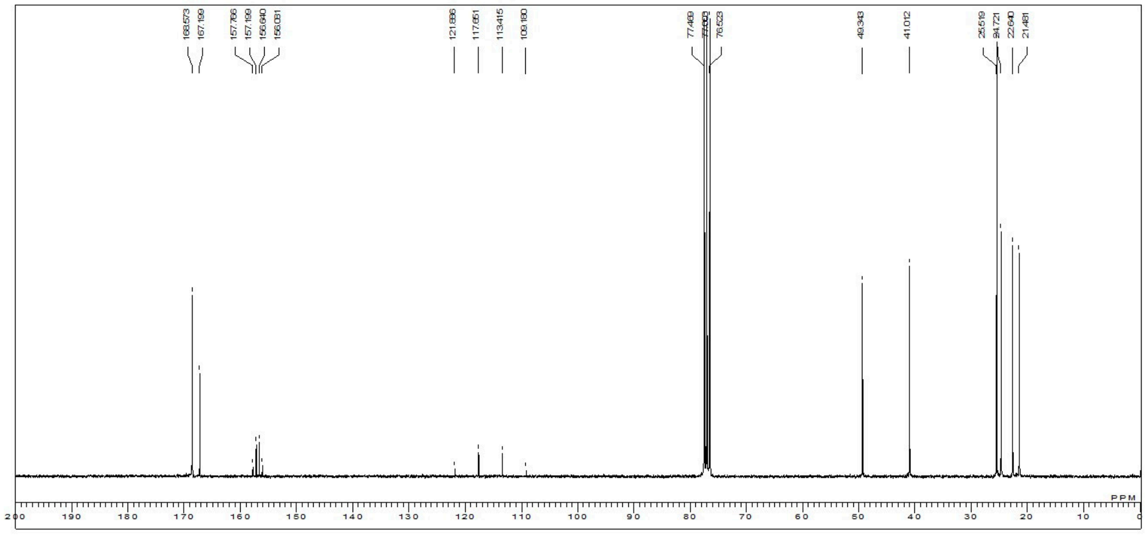

(*R*)-2,5-Dioxopyrrolidin-1-yl 4-methyl-2-(2,2,2-trifluoroacetamido)pentanoate (TFA-D-Leu-OSu, D-6b)

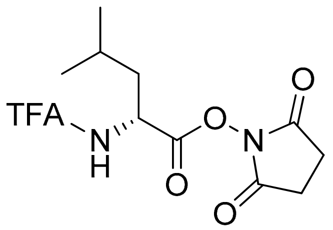

^{1}H-NMR (270 MHz, CD_3Cl_3)

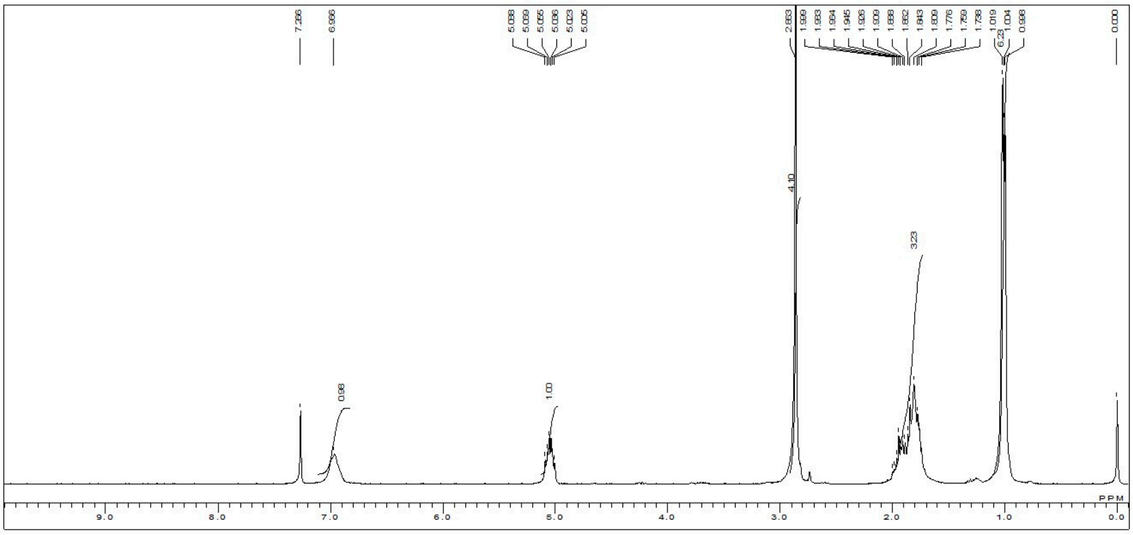

^{13}C-NMR (67.5 MHz, $CDCl_3$)

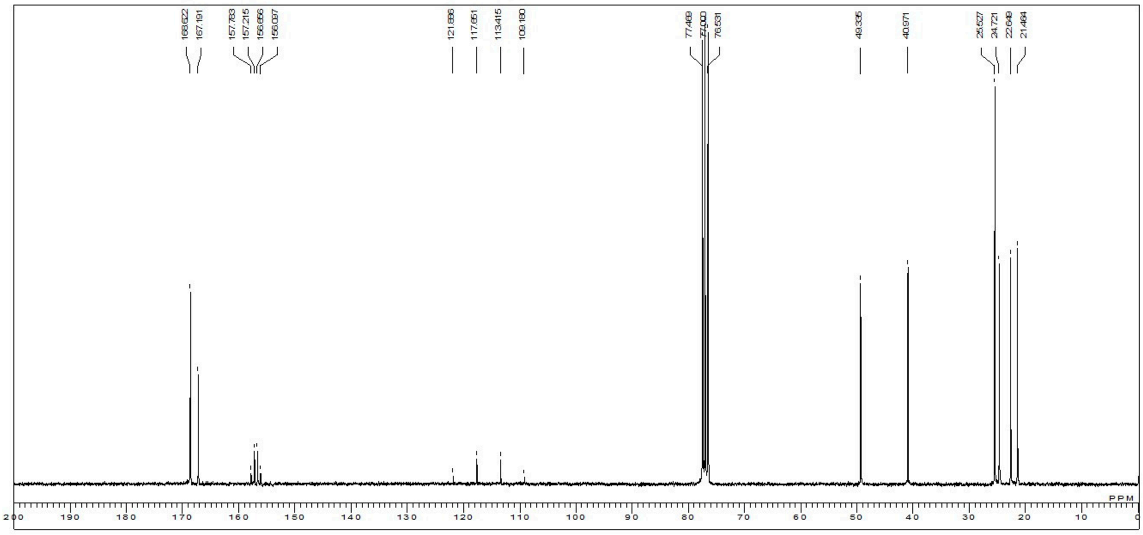

(*S*)-2,2,2-Trifluoro-*N*-(4-methyl-1-oxo-1-phenylpentan-2-yl)acetamide (TFA-L-Leu-Ph, L-6c)

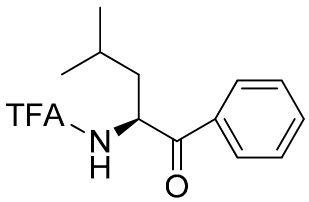

^{1}H-NMR (270 MHz, CDCl$_3$)

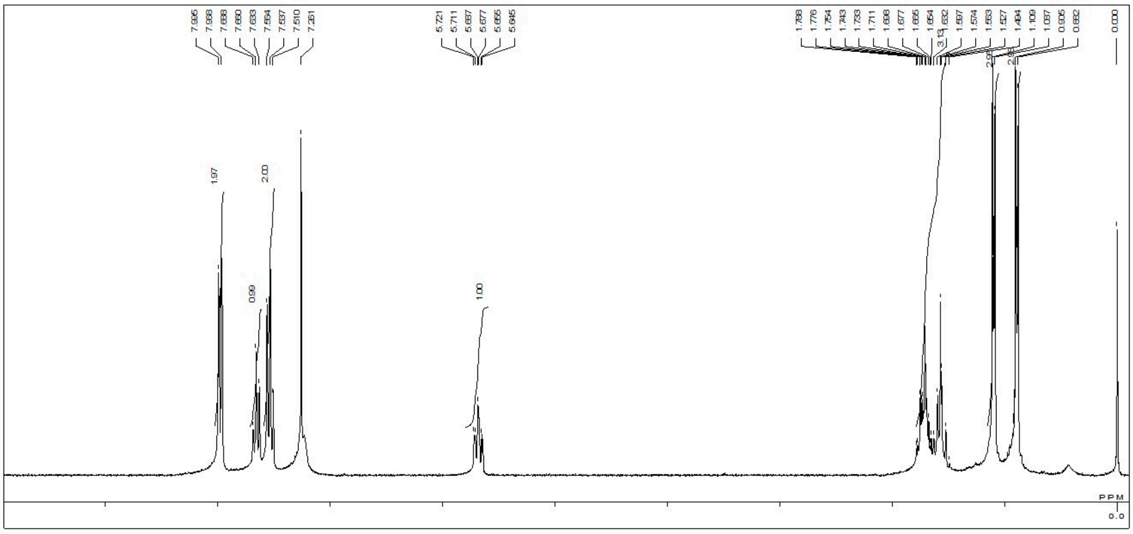

^{13}C-NMR (67.5 MHz, CDCl$_3$)

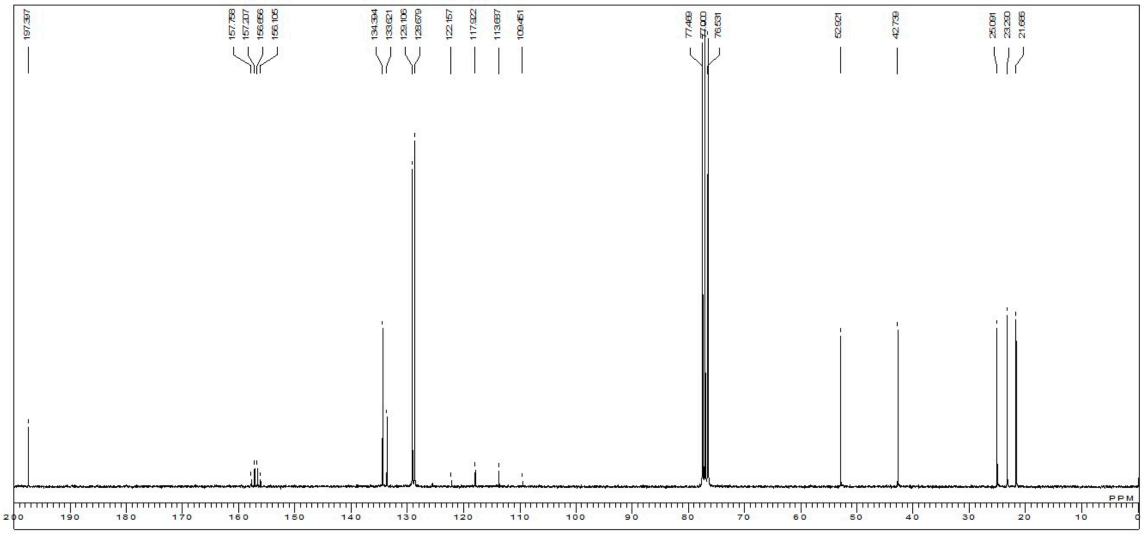

(*R*)-2,2,2-Trifluoro-*N*-(4-methyl-1-oxo-1-phenylpentan-2-yl)acetamide (TFA-D-Leu-Ph, D-6c)

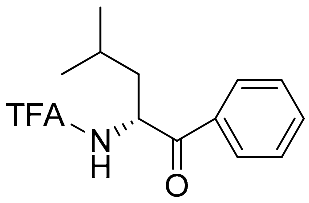

^{1}H-NMR (270 MHz, $CDCl_3$)

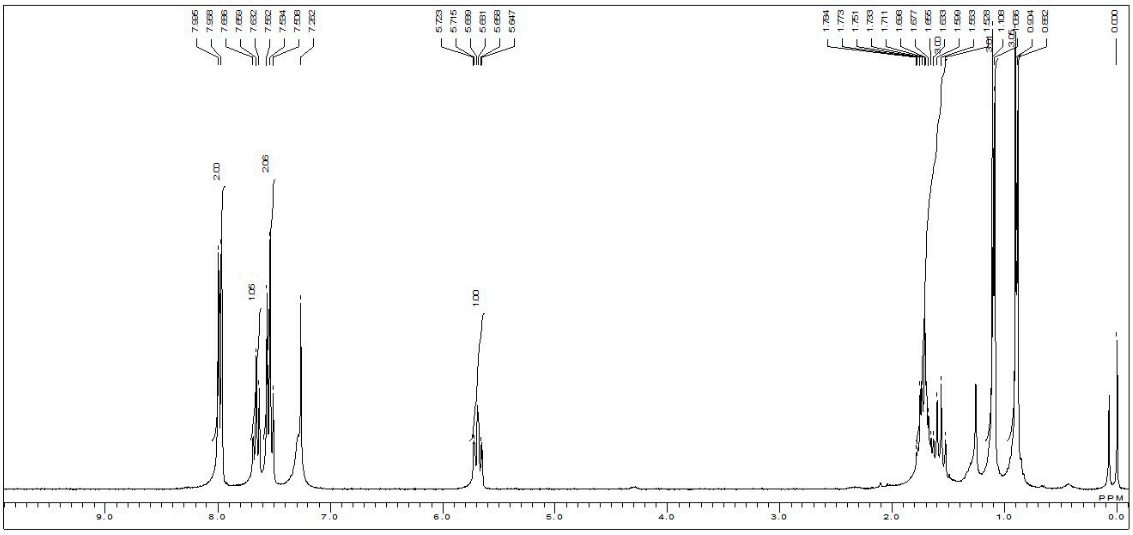

^{13}C-NMR (67.5 MHz, $CDCl_3$)

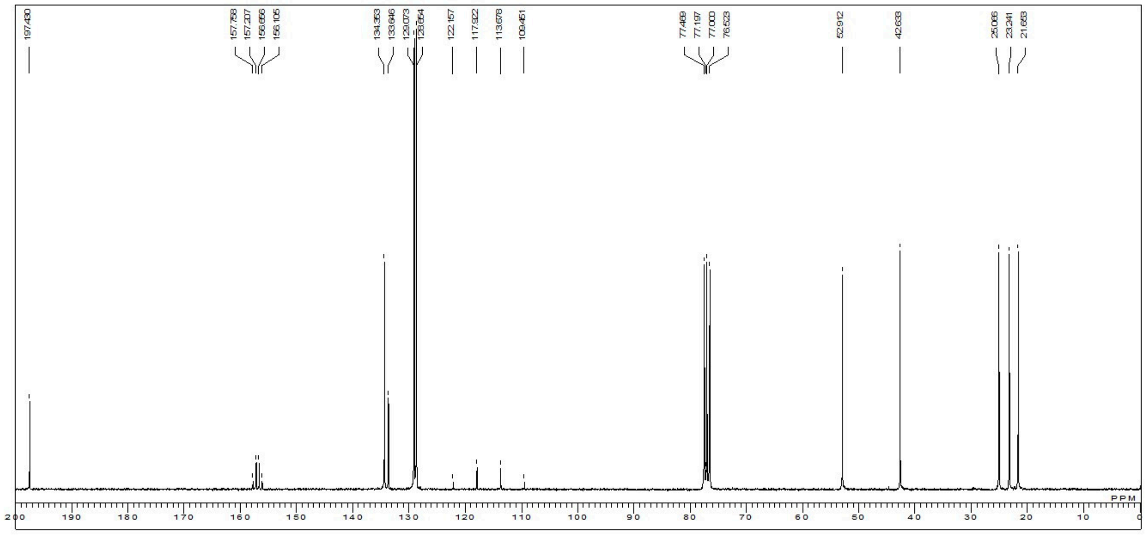

(*S*)-2-(2,2,2-Trifluoroacetamido)pentanoic acid (TFA-L-Nva, L-7a)

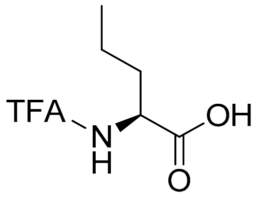

^{1}H-NMR (270 MHz, $CDCl_3$)

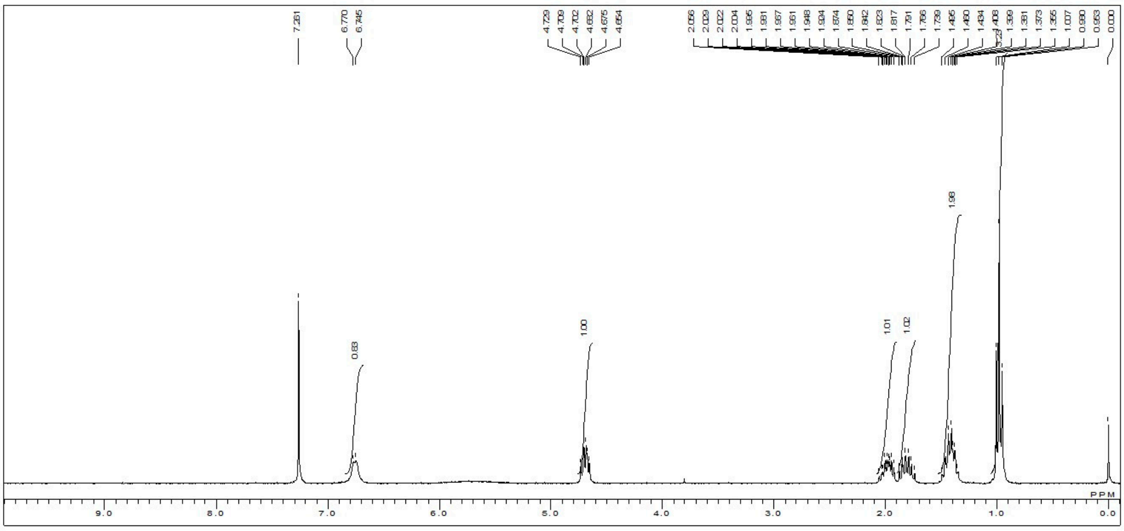

^{13}C-NMR (67.5 MHz, $CDCl_3$)

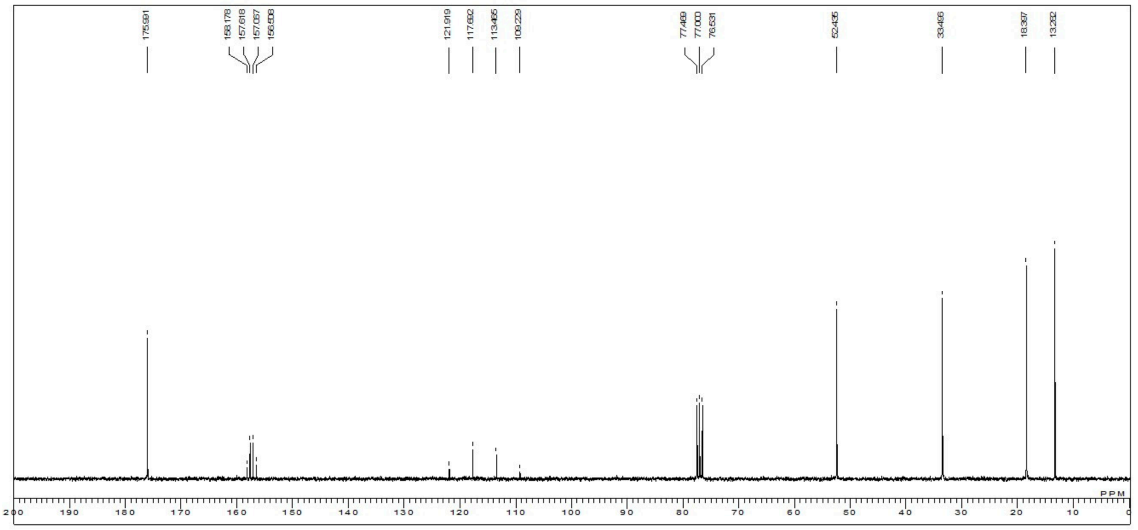

(*R*)-2-(2,2,2-Trifluoroacetamido)pentanoic acid (TFA-D-Nva, D-7a)

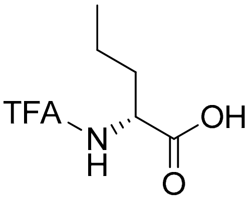

^{1}H-NMR (270 MHz, $CDCl_3$)

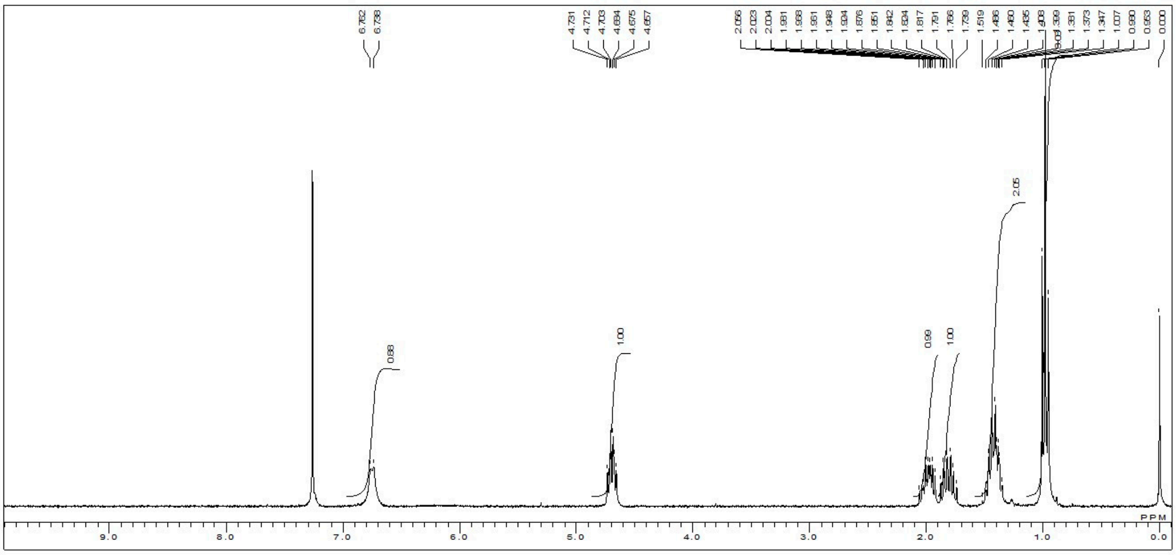

^{13}C-NMR (67.5 MHz, $CDCl_3$)

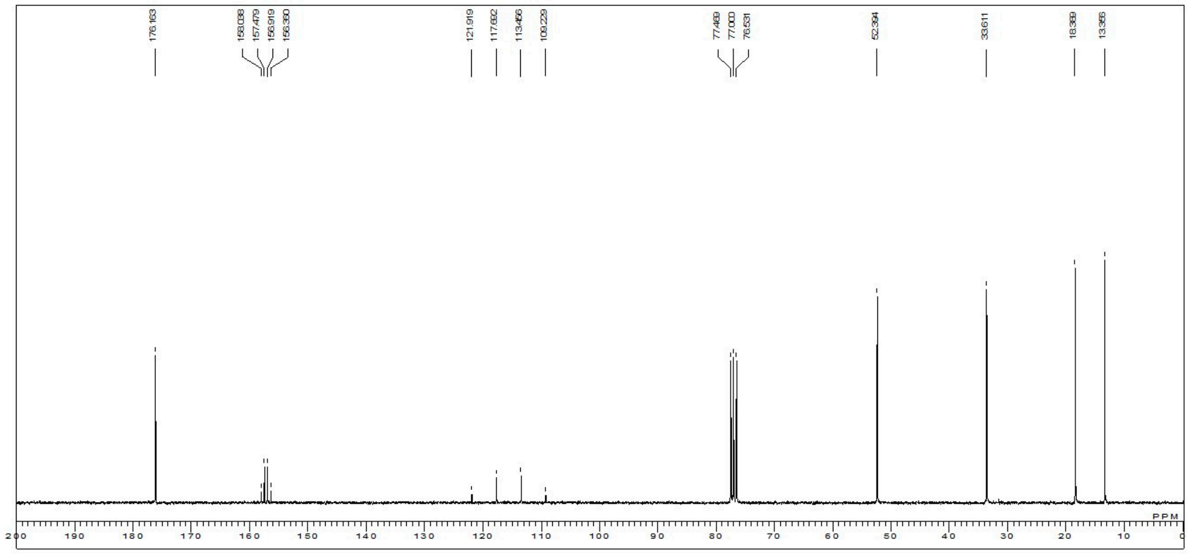

(*S*)-2,5-Dioxopyrrolidin-1-yl 2-(2,2,2-trifluoroacetamido)pentanoate (TFA-L-Nva-OSu, L-7b)

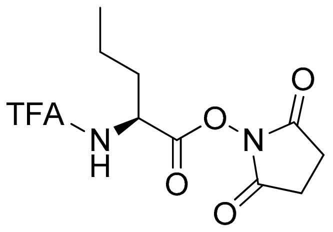

^{1}H-NMR (270 MHz, $CDCl_3$)

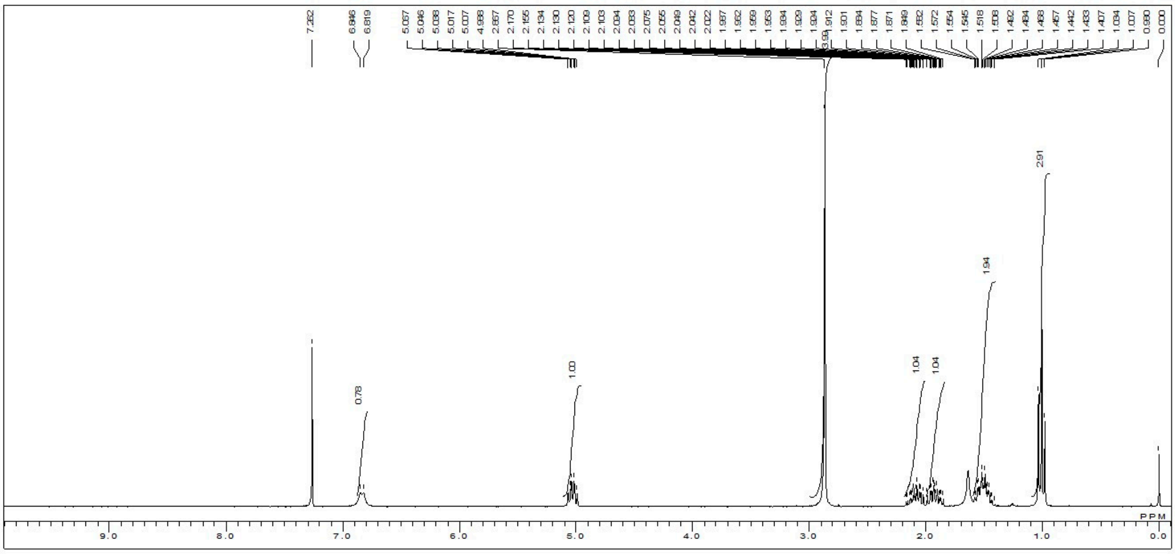

^{13}C-NMR (67.5 MHz, $CDCl_3$)

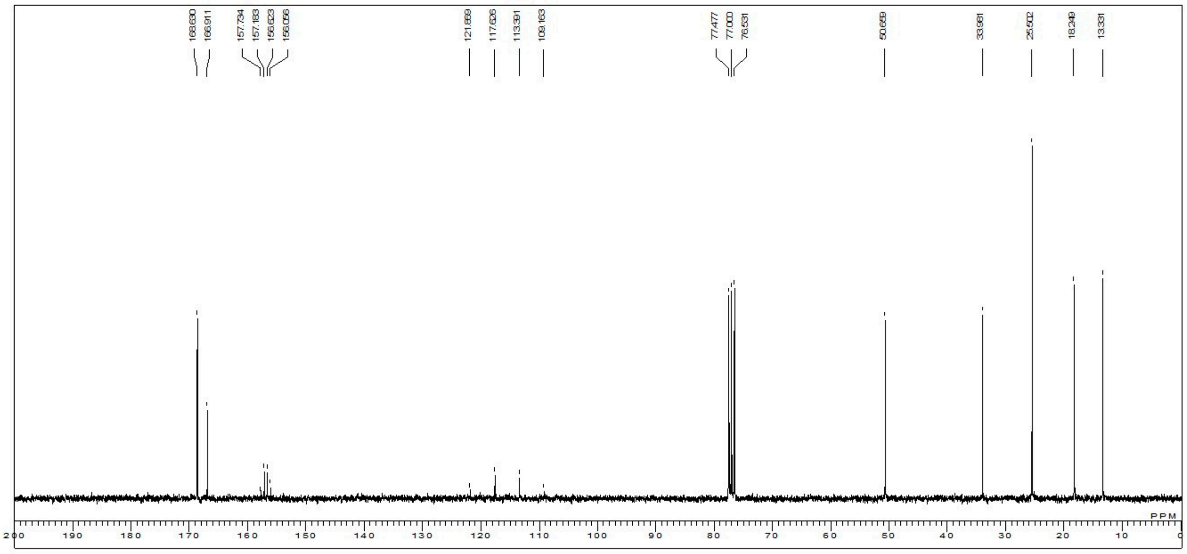

(*R*)-2,5-Dioxopyrrolidin-1-yl 2-(2,2,2-trifluoroacetamido)pentanoate (TFA-D-Nva-OSu, D-7b)

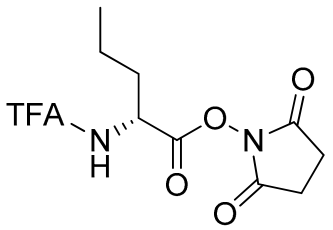

^{1}H-NMR (270 MHz, $CDCl_3$)

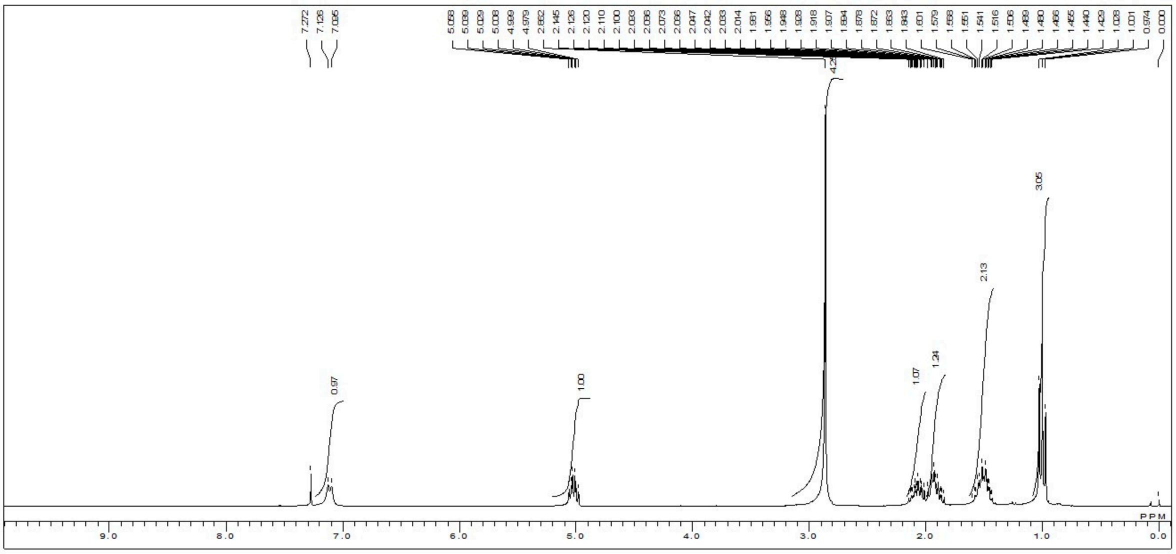

^{13}C-NMR (67.5 MHz, $CDCl_3$)

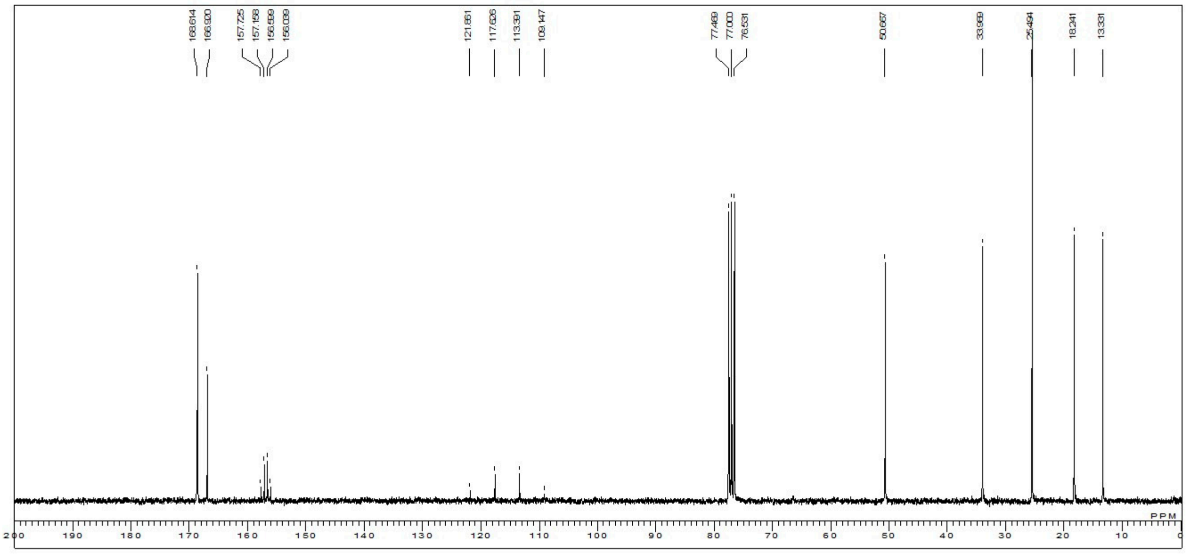

(*S*)-2,2,2-Trifluoro-*N*-(1-oxo-1-phenylpentan-2-yl)acetamide (TFA-L-Nva-Ph, L-7c)

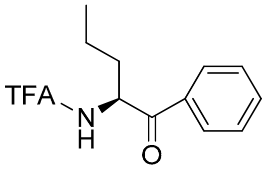

^{1}H-NMR (270 MHz, $CDCl_3$)

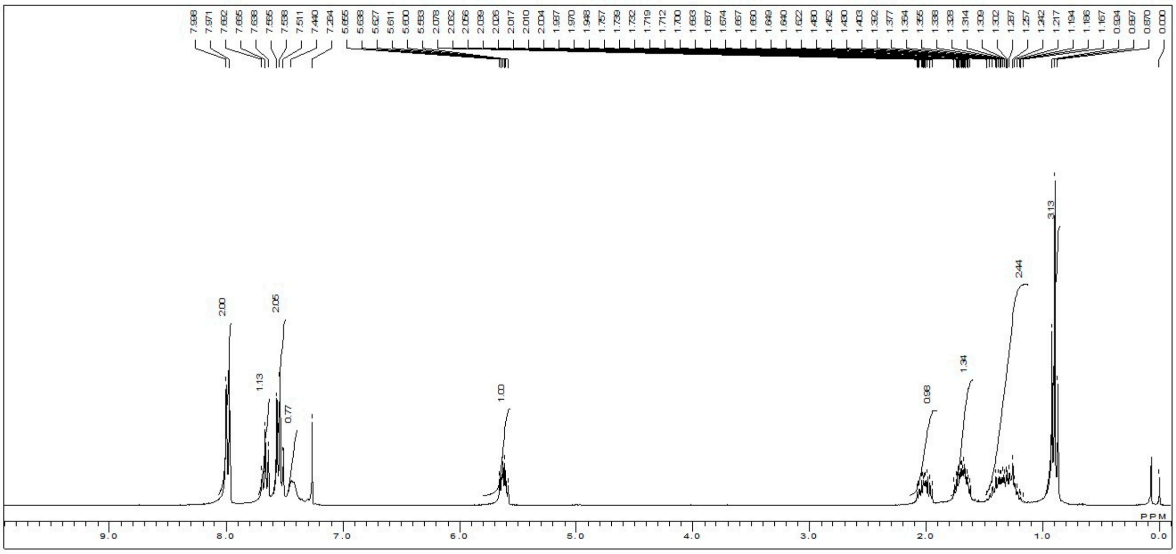

^{13}C-NMR (67.5 MHz, $CDCl_3$)

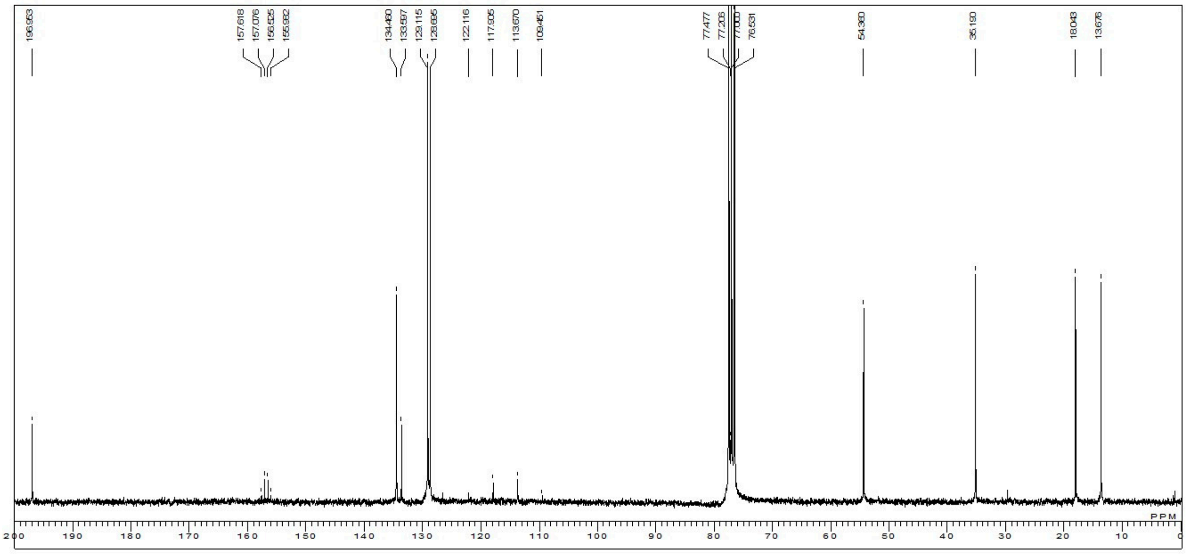

(*R*)-2,2,2-Trifluoro-*N*-(1-oxo-1-phenylpentan-2-yl)acetamide (TFA-D-Nva-Ph, D-7c)

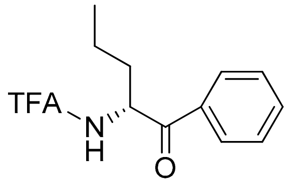

^{1}H-NMR (270 MHz, $CDCl_3$)

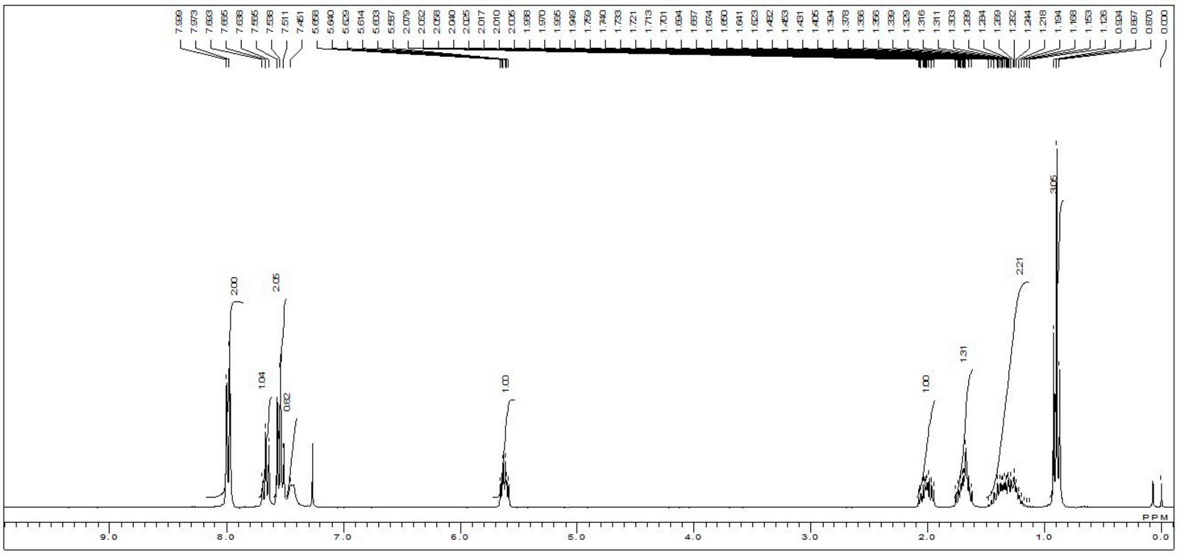

^{13}C-NMR (67.5 MHz, $CDCl_3$)

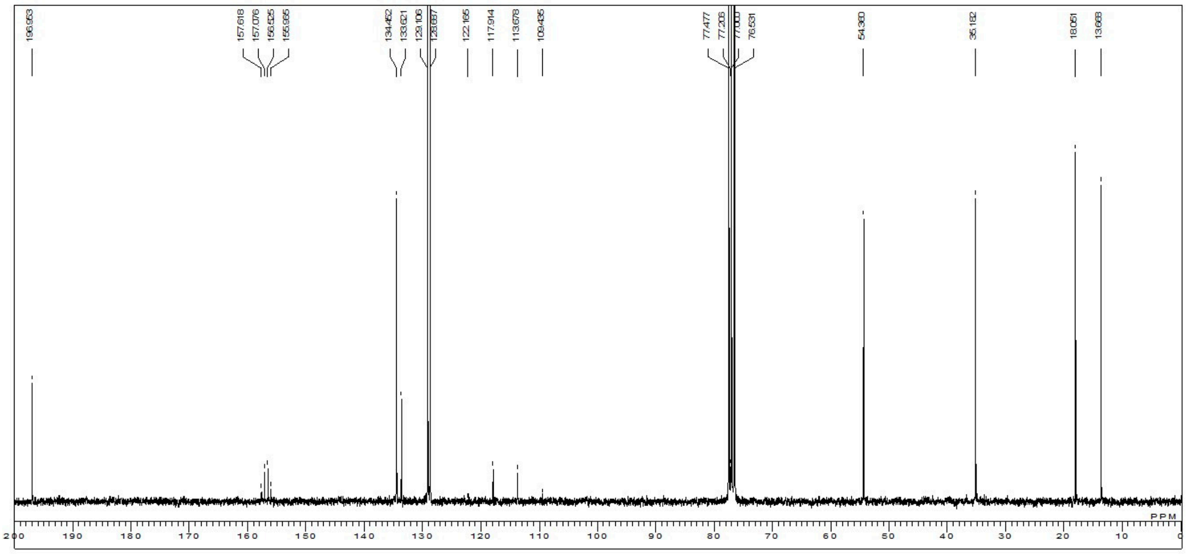

(*S*)-2-(2,2,2-Trifluoroacetamido)hexanoic acid (TFA-L-Nle, L-8a)

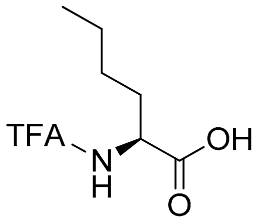

^{1}H-NMR (270 MHz, $CDCl_3$)

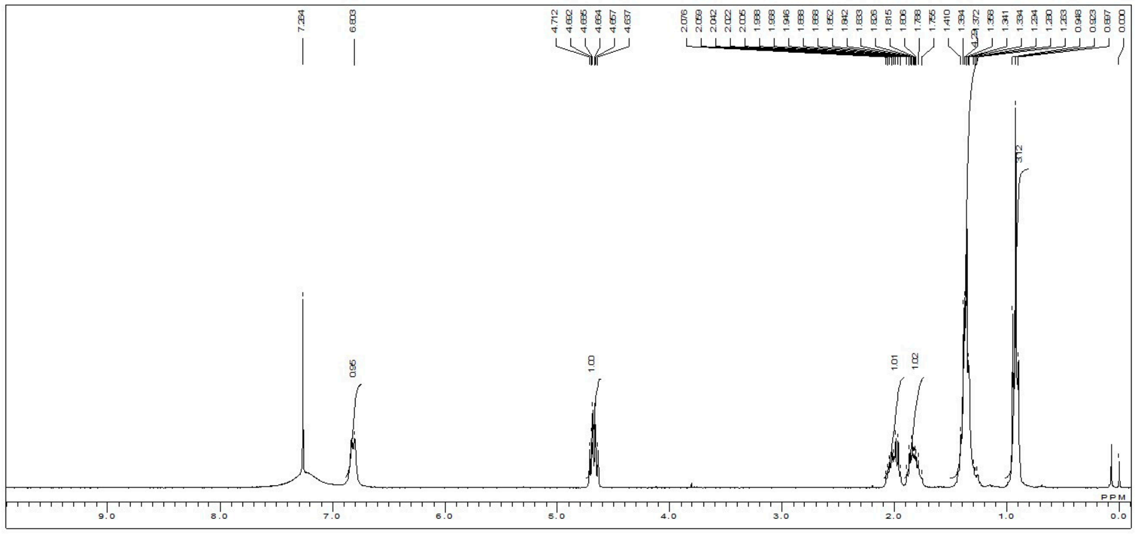

^{13}C NMR (67.5 MHz, $CDCl_3$)

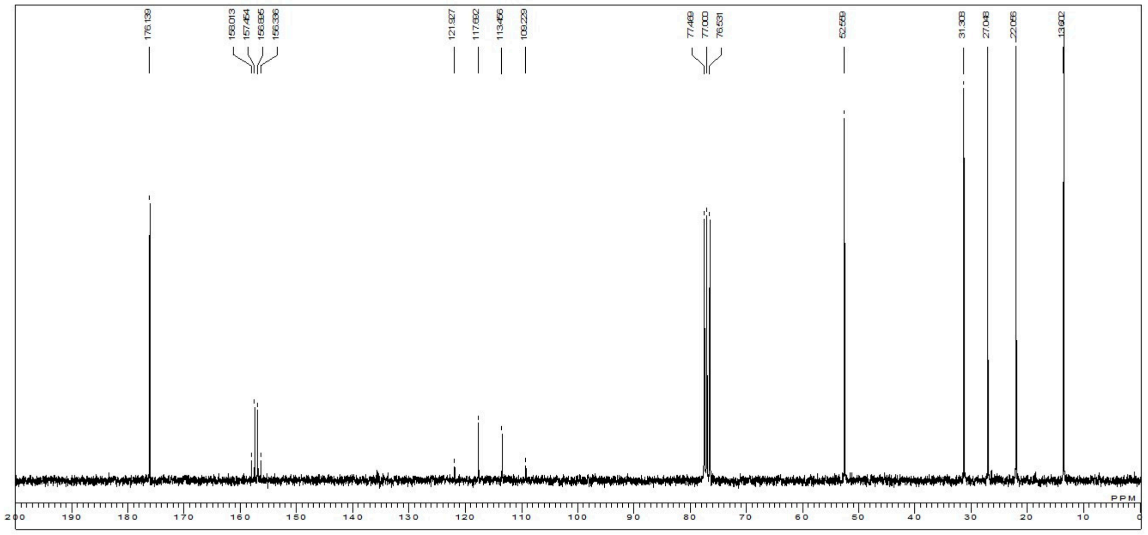

(*R*)-2-(2,2,2-Trifluoroacetamido)hexanoic acid (TFA-D-Nle, D-8a)

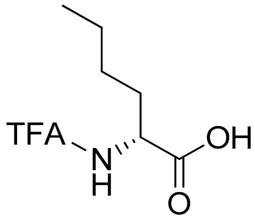

^{1}H-NMR (270 MHz, $CDCl_3$)

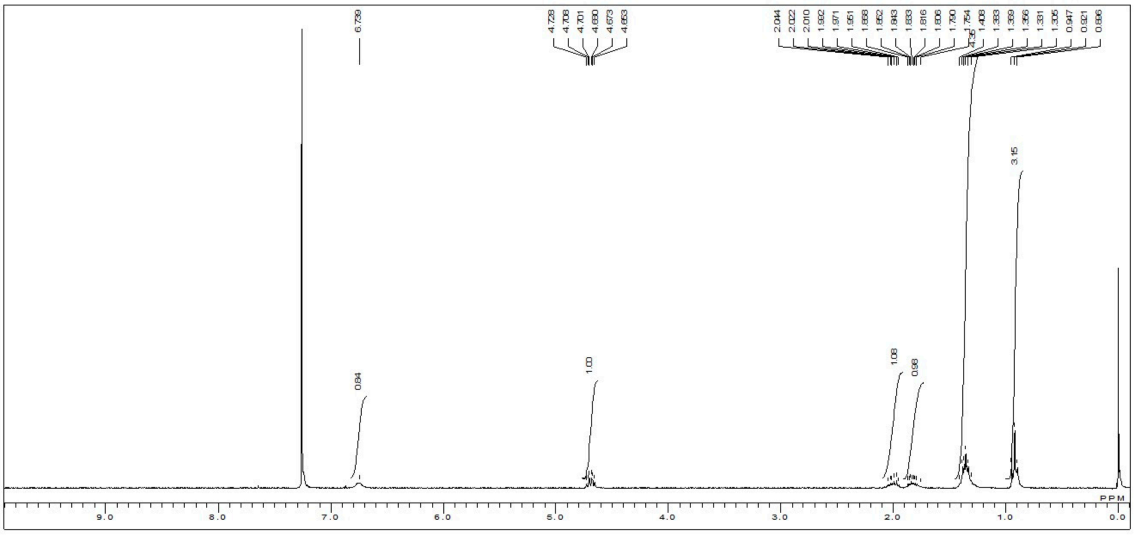

^{13}C NMR (67.5 MHz, $CDCl_3$)

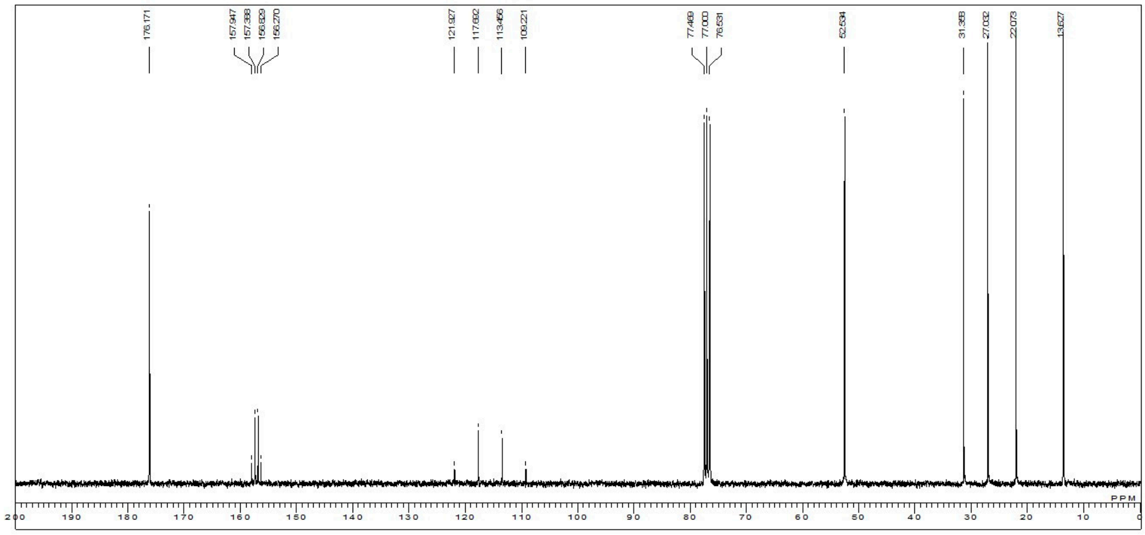

(*S*)-2,5-Dioxopyrrolidin-1-yl 2-(2,2,2-trifluoroacetamido)hexanoate (TFA-L-Nle-OSu, L-8b)

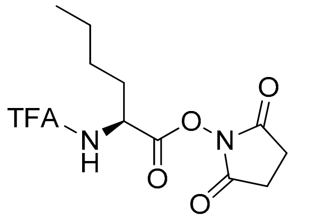

^{1}H-NMR (270 MHz, $CDCl_3$)

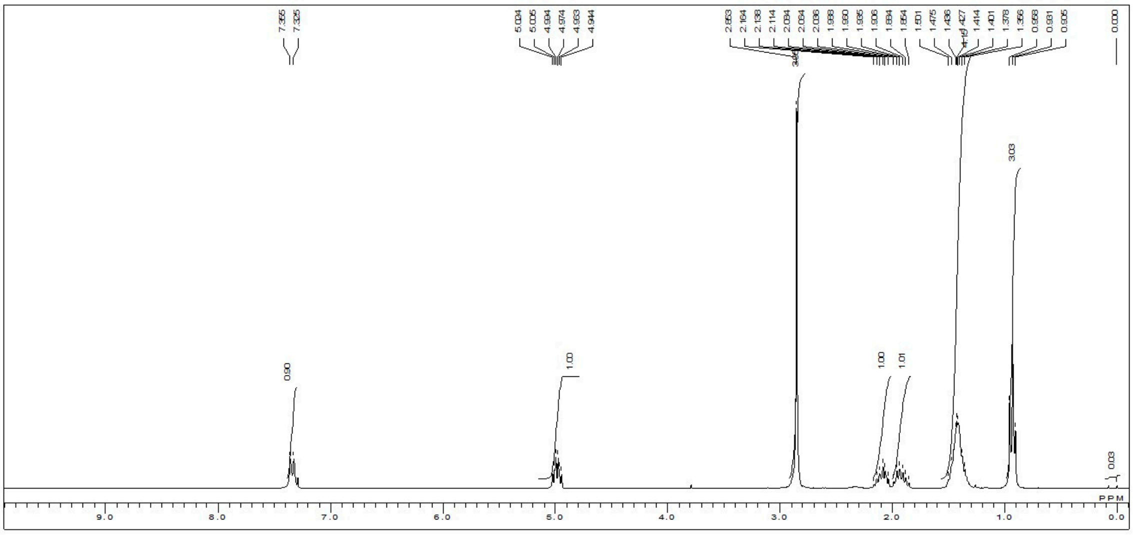

^{13}C NMR (67.5 MHz, $CDCl_3$)

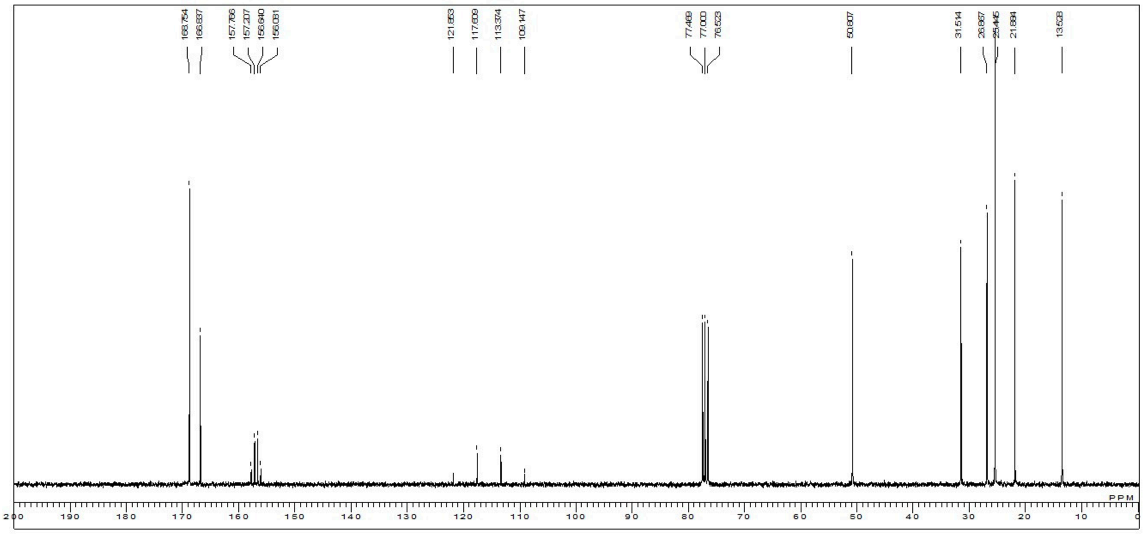

(R)-2,5-Dioxopyrrolidin-1-yl 2-(2,2,2-trifluoroacetamido)hexanoate (TFA-D-Nle-OSu, D-8b)

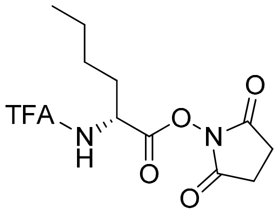

^{1}H-NMR (270 MHz, $CDCl_3$)

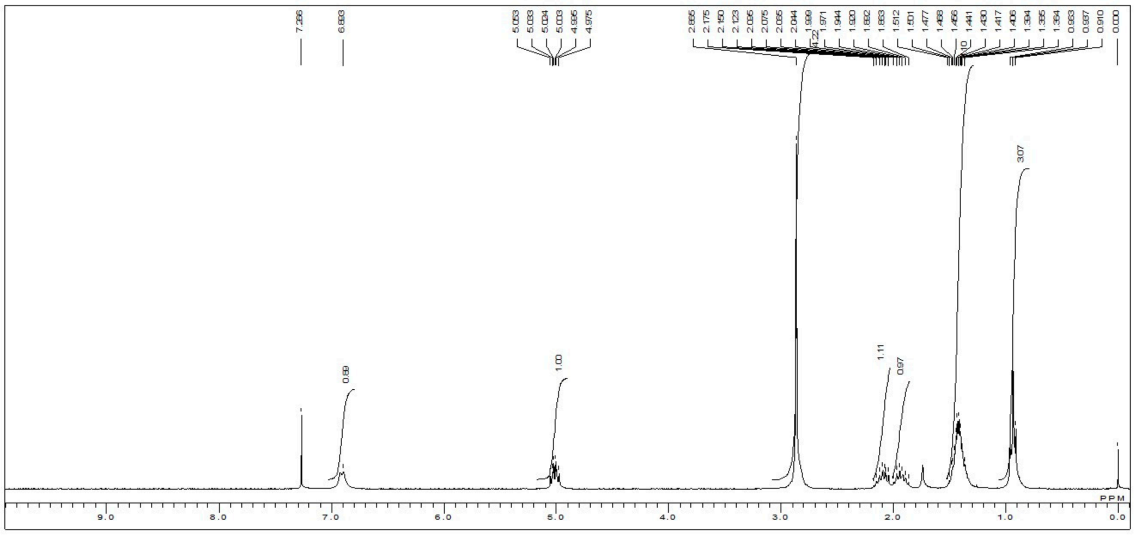

^{13}C-NMR (67.5 MHz, $CDCl_3$)

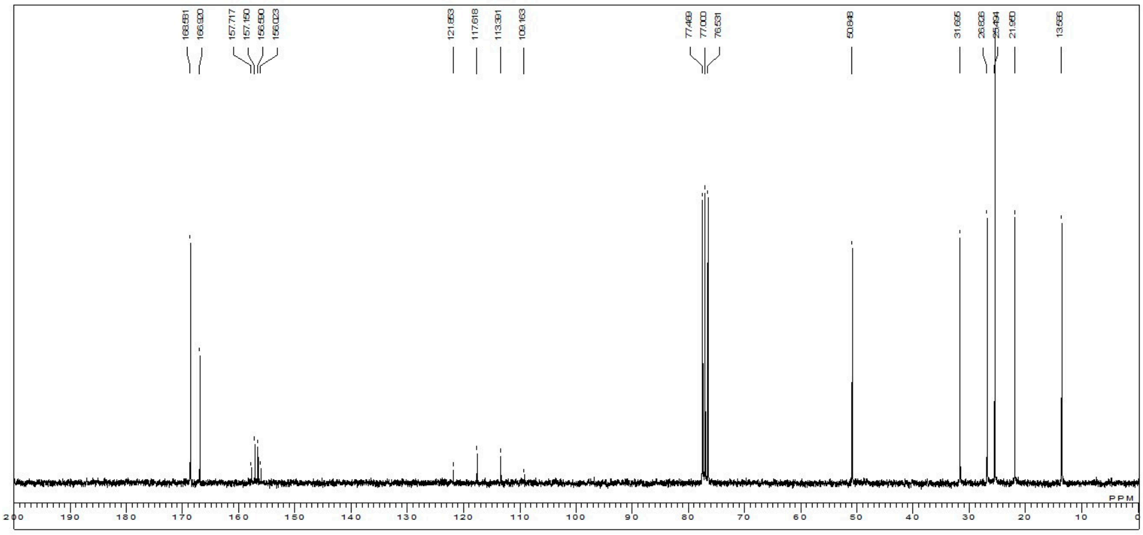

(*S*)-2,2,2-Trifluoro-*N*-(1-oxo-1-phenylhexan-2-yl)acetamide (TFA-L-Nle-Ph, L-8c)

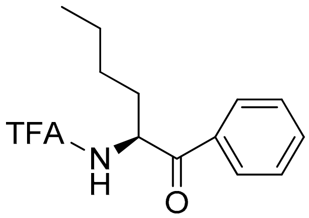

^{1}H-NMR (270 MHz, $CDCl_3$)

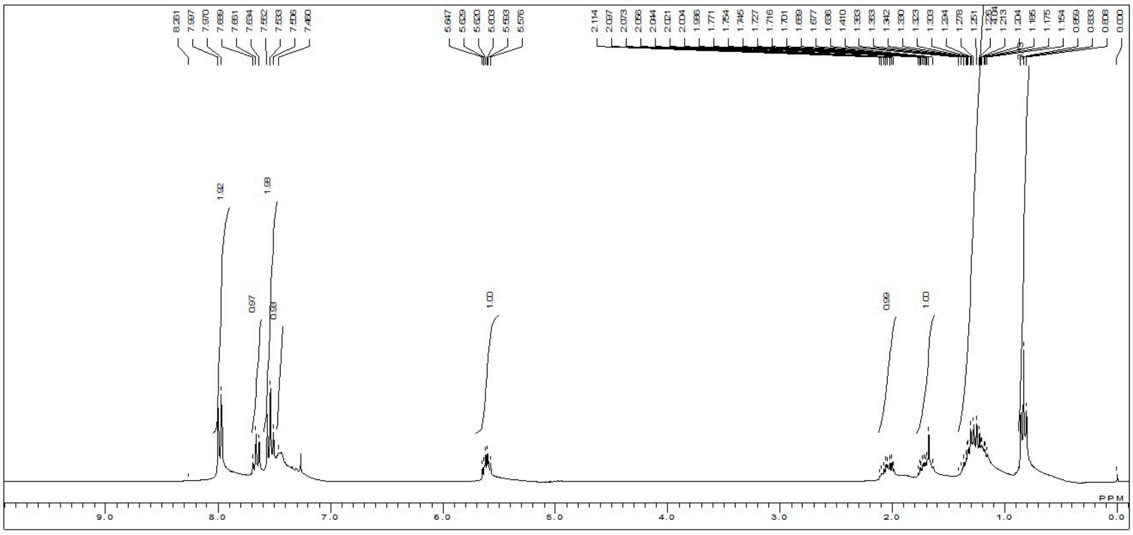

^{13}C-NMR (67.5 MHz, $CDCl_3$)

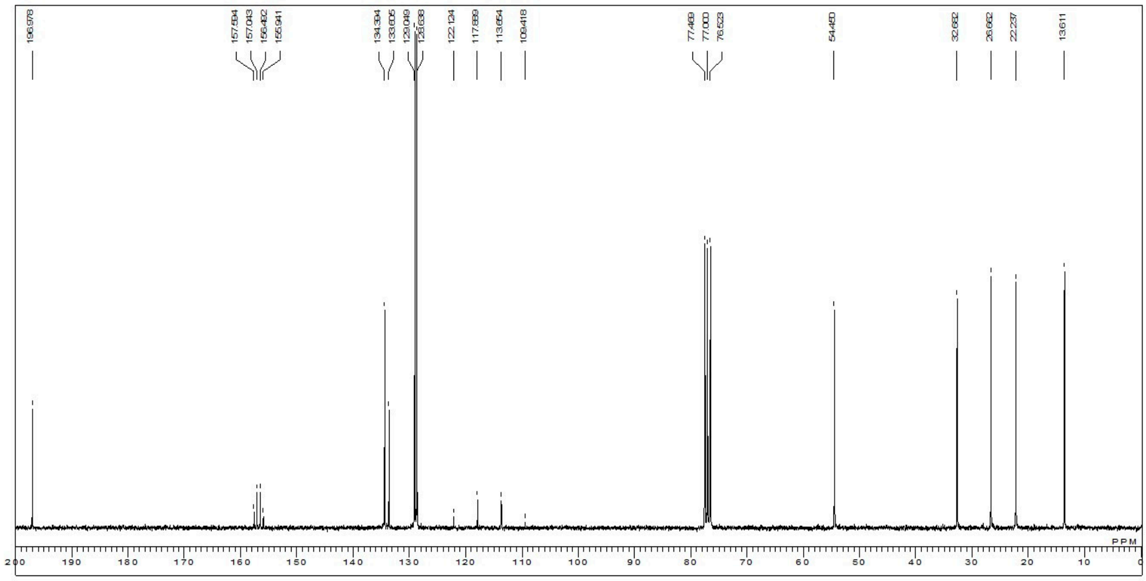

(*R*)-2,2,2-Trifluoro-*N*-(1-oxo-1-phenylhexan-2-yl)acetamide (TFA-D-Nle-Ph, D-8c)

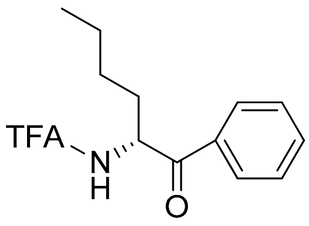

^{1}H-NMR (270 MHz, CDCl$_3$)

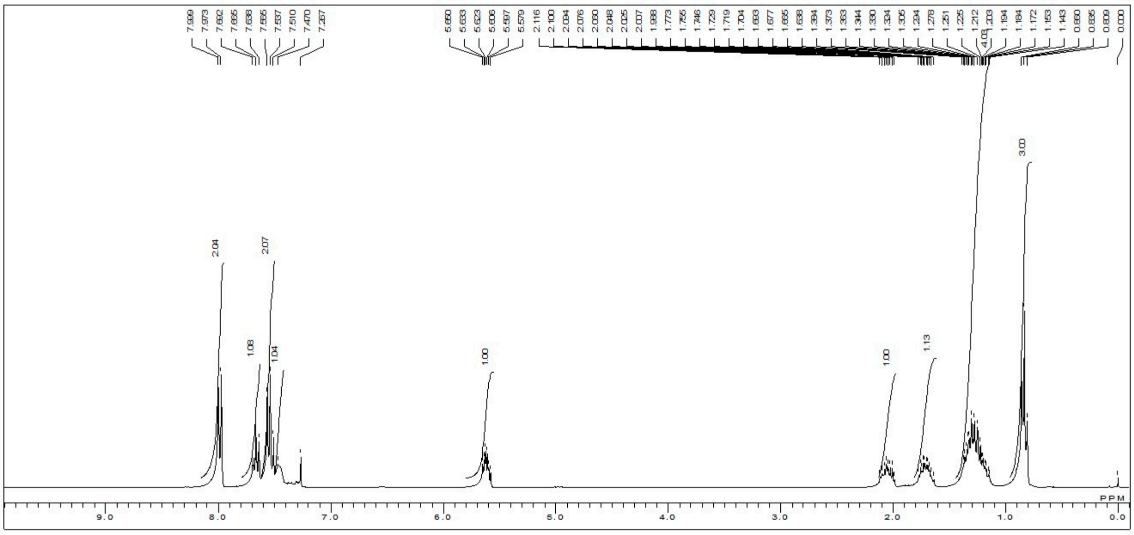

^{13}C-NMR (67.5 MHz, CDCl$_3$)

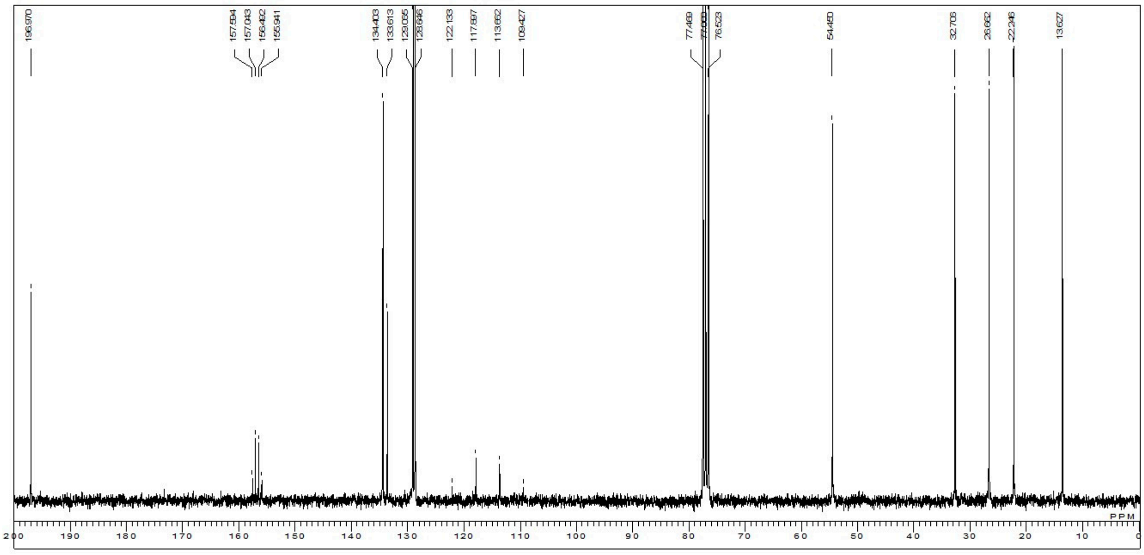

Supplement: Supplementary file 1 [file molecules-22-01748-s001.pdf]
